# Developing an Internationally-Applicable Service Specification for Continence Care: Systematic Review, Evidence Synthesis and Expert Consensus

**DOI:** 10.1371/journal.pone.0104129

**Published:** 2014-08-14

**Authors:** Adrian S. Wagg, Diane K. Newman, Kai Leichsenring, Paul van Houten

**Affiliations:** 1 University of Alberta, Department of Medicine, Edmonton, AB, Canada; 2 University of Pennsylvania, Division of Urology, Philadelphia, Pennsylvania, United States of America; 3 European Centre for Social Welfare Policy and Research, Vienna, Austria; 4 Zonnhuisgroep Amstelland, Elderly Medicine, Amstelveen, the Netherlands; University of Glasgow, United Kingdom

## Abstract

**Background:**

Global demographic trends suggest that the incidence of both urinary and faecal incontinence will rise in the coming years, bringing significant health and economic implications for both patients and payers. There is limited organisational evidence to guide payers and providers about service configuration which will deliver efficient guideline-compliant, high-quality patient care.

**Objectives:**

To create, using evidence from a systematic review, qualitative data and expert consensus an internationally applicable service specification for continence care.

**Method:**

Evidence was obtained from a systematic and grey literature review of published randomised controlled trials and quasi-experimental studies reporting efficacy of continence service design at the level of the community dwelling patient with either bladder or bowel incontinence, governmental reports and policy frameworks supplemented by data from 47 semi-structured interviews with clinicians, patients, patient-representatives and policy experts from four geographies broadly representative of different healthcare systems.

**Results:**

A number of themes related to current and potential future organisation of continence care were identified from the data. A modular service specification with eight core components was created including case detection, initial assessment and treatment, case co-ordination, caregiver support, community-based support, specialist assessment and treatment, use of containment products, and use of technology. Within this framework important key recommendations are: ensure robust referral pathways, shift assessment for case coordination to nurses specializing in continence care, promote self-management and technology, use comprehensive assessment tools and service performance targets based on outcome and operational measures.

**Conclusions:**

This study has defined practice gaps in the provision of continence services and described eight core components of a service specification for incontinence that commissioners and payers of health and social care could consider using to provide high-quality continence care. A shift towards a community-delivered, nurse-led model appears to be associated with clinical and cost-effective care for people with bladder and bowel incontinence.

## Introduction

Global demographic and clinical trends suggest that the incidence of both urinary and faecal incontinence will rise sharply in the coming years with significant health and economic implications for both patients and payers [1]. In parallel, the need to constrain health and social care spending has forced payers to reconsider the overall structure and extent of provision in health and social care systems, and move towards more coordinated or integrated service delivery in order to bridge the health/social care divide [2]. At the organisational level, there is limited evidence to guide payers and providers about how continence care might best be configured to deliver efficient, guideline-compliant, high-quality patient care at either the same, or lower, overall cost [3].

### Impact

The prevalence of urinary incontinence (UI) in community-dwelling middle-aged and older women is estimated to be between 30% and 60% depending on definition and the population in which the study occurred [4], [5], [6], [7], [8], while the pooled overall prevalence rates of UI in community-dwelling men range from 5% to 32% [1]. Faecal incontinence (FI) occurs at all ages and in both sexes, affecting 1% to 20% of adults, depending on the definition used by individual studies [1].

The impact of incontinence on quality of life is significant [9], and includes social embarrassment, reduced employment, work productivity and leisure opportunities, social exclusion, and a significant strain on the relationships between patients and their partners or informal caregivers [10]. The Canadian National Population Health Survey (1996/7) rated the effect of UI behind only Alzheimer’s and stroke in people aged 12 and older, and highest in younger patients (those aged 45 years old and under) [11]. A more recent study in Taiwanese women has confirmed the greater impact of incontinence on quality of life compared with other major chronic conditions [12].

There is also a significant impact on the caregiver; incontinence is one of the most common reasons for a person moving to long-term institutional care [1], [13]. In studies, caregivers found dealing with the following factors particularly difficult: the care-giving burden of managing urine leakage and/or taking the patient to the toilet regularly [14], psychological strain due to the full-time nature of the role [15], restriction in leisure opportunities and social interactions [16], changes in the nature of their relationship e.g. daughter/son or wife/husband to intimate nursing role [14], and feelings of guilt causing the caregiver to ‘hang on’ to responsibility for caring past the point where they are truly managing the care load [17].

Incontinence presents a significant health and economic burden comparable with major global diseases such as arthritis and pneumonia [18]. There are direct costs related to health and social care – these may be covered by the public payer/health insurer, out of pocket payments by the patient or caregiver, or a mixture of the two [19], [20]. There are also indirect costs such as the opportunity costs of caregivers and patients, e.g. removal from the workforce [20]. The health economic impact of incontinence is projected to increase substantially in developed countries as populations age [18].

### Current problems with service delivery

Current continence care service delivery does not adequately address this health and social care burden [21]. This is particularly true for case finding and provision of initial treatments, perhaps as a result of the relative lack of continence-related content in higher education training across the major healthcare professional disciplines [22], [23].

Current evidence suggests that routinely trying conservative treatments before surgical interventions is likely to be the most cost-effective treatment strategy [24], [25], [26]. Community-delivered continence services do appear to be cost effective [27].

Low levels of integration of continence services result in duplication of provision, concentration of services in specialist centres and relatively low provision of community-based care [21], [28]. Additionally, provision may be dictated by imbalances in the level of reimbursement versus the true cost of providing treatment. For example, in the United States, fee-for-service reimbursement has incentivised specialist provision of non-specialist activities across many common medical conditions [29]. In the case of continence care, this results in a bias towards surgical intervention over more conservative treatment strategies [30], [31], as well as overuse of expensive and often unnecessary investigations such as urodynamic testing [32].

In many countries, but particularly low income ones, incontinence is not usually a priority with only basic levels of practice performed by community care providers, if at all [33]. Many of the problems in continence care in these countries are related to the immaturity of the wider healthcare system [3]. Addressing continence care needs while these countries develop their healthcare infrastructure will likely require innovative solutions to make the most of limited resources.

Given the economic impact of incontinence and the lack of comprehensive services for continence care globally, this study aimed to provide an evidence-based specification for the procurement and organisation of continence care.

## Method

A multiprofessional expert panel from a wide range of disciplines and geographies was convened to define an ‘optimal’ service specification for continence care for community-dwelling adults based on best available evidence. Community-dwelling adults were selected because providing high-quality care to this group may delay the transfer of older people to institutional care, and incontinence is often one of the main reasons older people move to a residential facility. The panel aimed to design a specification able to take account of local variations in practice, resource and culture.

The NICE accredited guidelines [34] on service specification design were followed; the method comprised three phases: evidence gathering; synthesis of evidence and drafting of the service specification; and validation of the specification.

### Evidence gathering

Recognising the paucity of data in this area, evidence was obtained from a variety of sources. The grey literature was also searched to include governmental and professional society reports and guidelines and relevant policy documents. Data were supplemented by qualitative findings from semi-structured interviews with clinicians, patients, patient-representatives and policy experts around the world.

#### Systematic Literature Review

A systematic literature search of online databases for articles relating to the organisation of continence care services included: Scopus, MEDLINE/PubMed, EMBASE, and the Cochrane Library from 2003 to April 2013 using the search terms: *continence*, *incontinence*, *care*, *service*, *services*, *specification*, *review*, *design*, *delivery*, *program*, *programme*, *expert standard*, *guidelines*, *good practice*, *models of excellence*. We retrieved randomised trials, quasi-experimental studies and other reports describing the effectiveness of continence service delivery on patient outcomes compared to either usual care or no intervention at any length of follow up. Further articles were retrieved through citation-tracking of original articles and systematic reviews, as well as through investigation of the grey literature.

Non-English language references were excluded unless there was sufficient explanatory text in English. Articles had to be relevant to continence care provided for community-dwelling adults. Articles based on children or on individuals in long-term care/institutionalised settings, and those focusing on primary prevention were excluded.

#### Semi-structured interviews

Forty-seven interviews were conducted with representatives from a variety of backgrounds based in different geographies. Interviewees included: payers, policy experts and influencers, academic researchers with an interest in incontinence, patients, patient group representatives, caregivers, physiotherapists, nurses, urologists, gynaecologists and primary care practitioners. Potential interviewees were identified from the literature search and approached to take part in the study.

**Figure 1:**
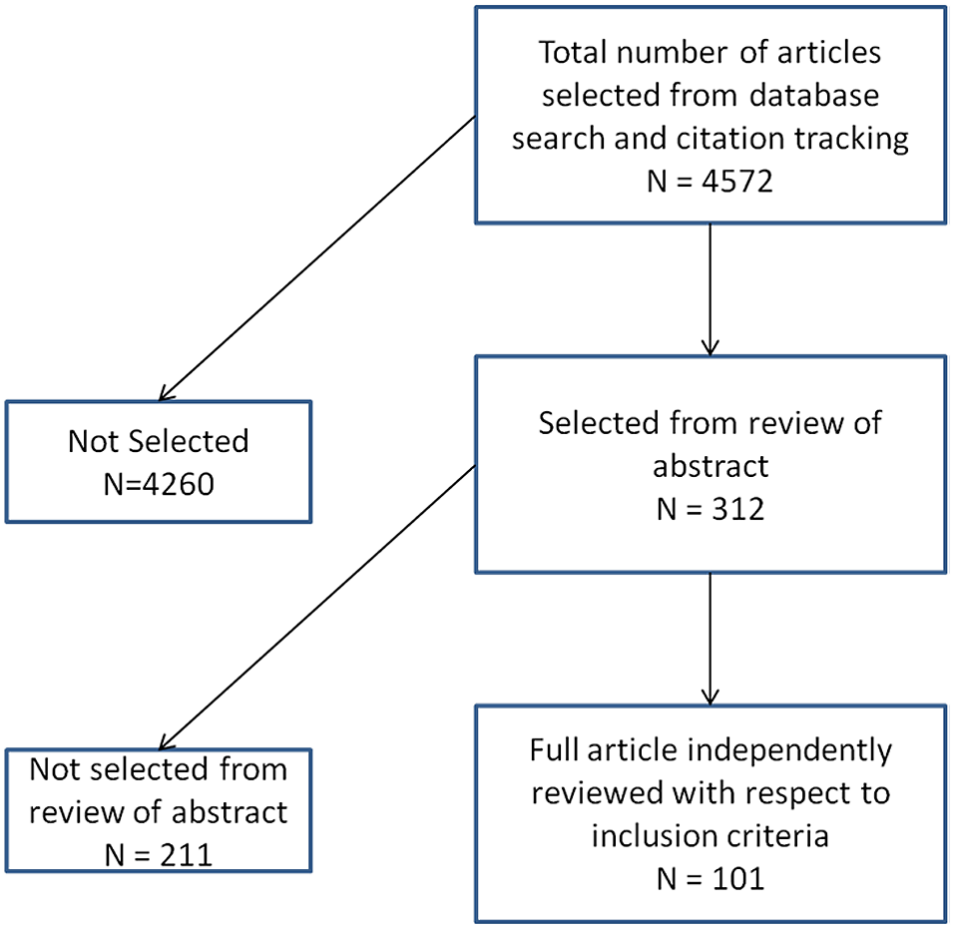
Interviewees by country.

For the interviews there was a focus on four particular countries (Figure 1): United Kingdom, Netherlands, United States and India. These four countries represent a spectrum of different healthcare/funding systems: socialised (UK), social insurance (Netherland), out-of-pocket (India) and varied (US).

**Figure 2:**
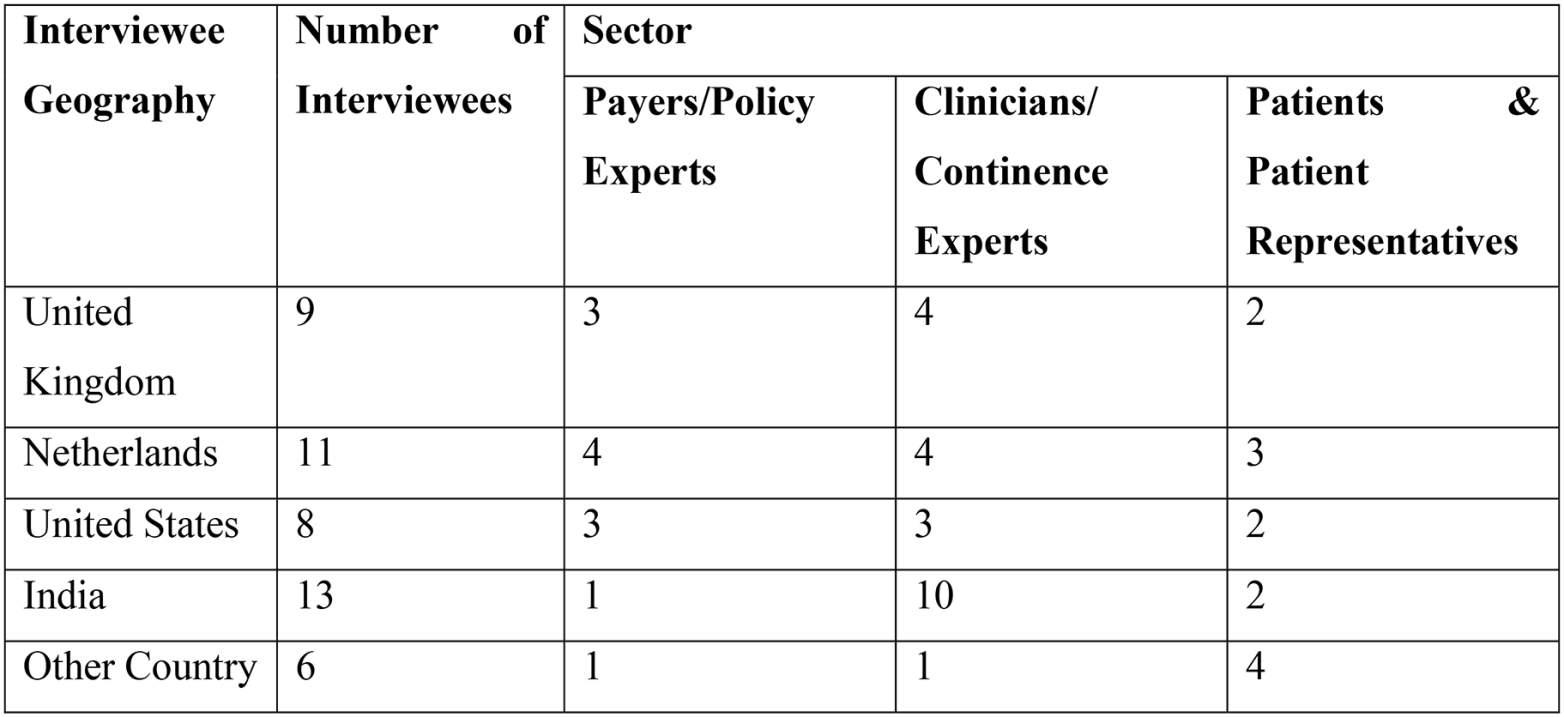
Interview subject areas and questions.

Interview questions (Figure 2) covered five main subject areas: the policy context, public awareness of incontinence, current service provision in continence care, care outcomes, and patient groups and access.

### Synthesis of evidence and drafting of service specification

**Figure 3:**
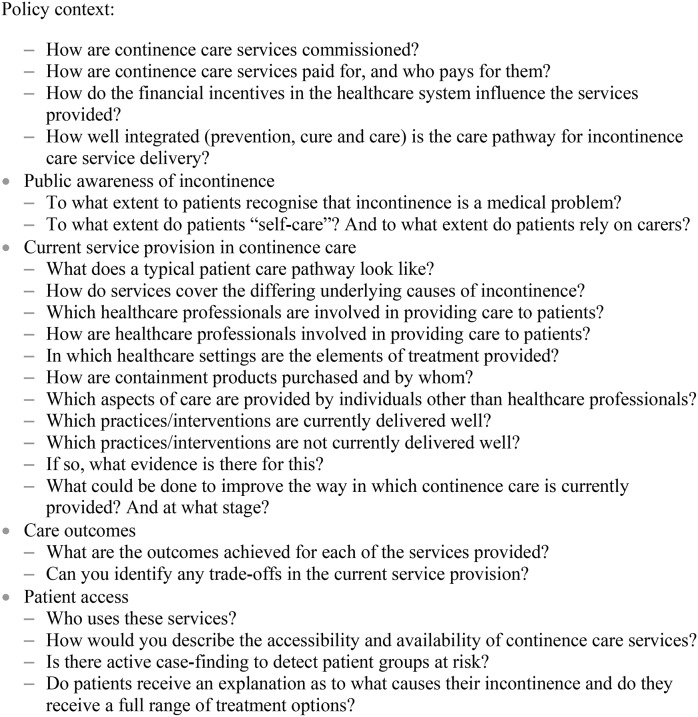
Underlying design principles in the development of a care service specification for incontinence.

Authors agreed on the principles to which they would adhere when considering the collected evidence and drafting the service specification (Figure 3).

The articles were read and interview findings noted. Key themes were identified and explicitly incorporated into discussions amongst the panel. Consensus method was used to establish the key recommendations of the service specification. Where there was disagreement regarding the evidence or the rationale for/against a particular point a majority opinion was required for resolution. There were no instances where recourse to formal voting was required. The four members of the panel, along with the research team, met twice face to face and fortnightly by teleconference throughout the conduct of the study. There were five formal evidence synthesis meetings during the conduct of the study, all conducted by teleconference.

### Validation of service specification

**Figure 4:**
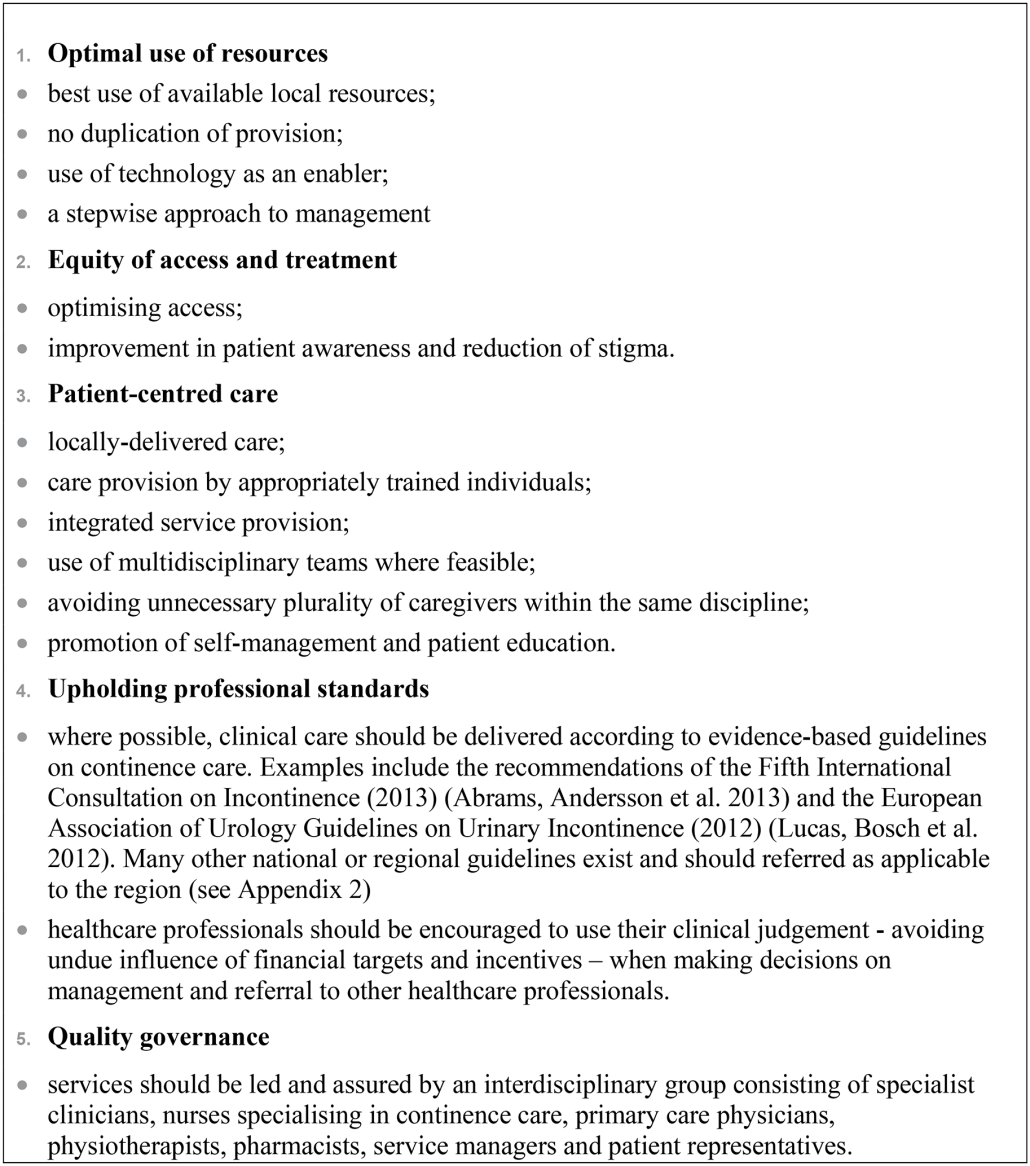
Patient profiles.

In order to confirm the broad applicability of the service specification in various countries, the draft service specification was sent back out to a number of experts for review. In particular, interviewees were asked to comment on the appropriateness of the service specification for their particular geography, but also with respect to four patient profiles which the EP developed (Figure 4). This is because each profile of patient has its own specific health and social care considerations, including specific clinical guidelines. Feedback was noted and discussed by the EP. Consensus method was again used to decide whether there was sufficient evidence to change the service specification.

### Ethical approval

The conduct of this study including consent procedure was approved by the Human Research Ethics Board of the University of Alberta. Given that the interviews were conducted over the telephone covering differing countries and time zones, informed verbal consent from interview participants was gained prior to the interview and affirmed in writing by the interviewer.

## Results

### Search results

**Figure 5:**
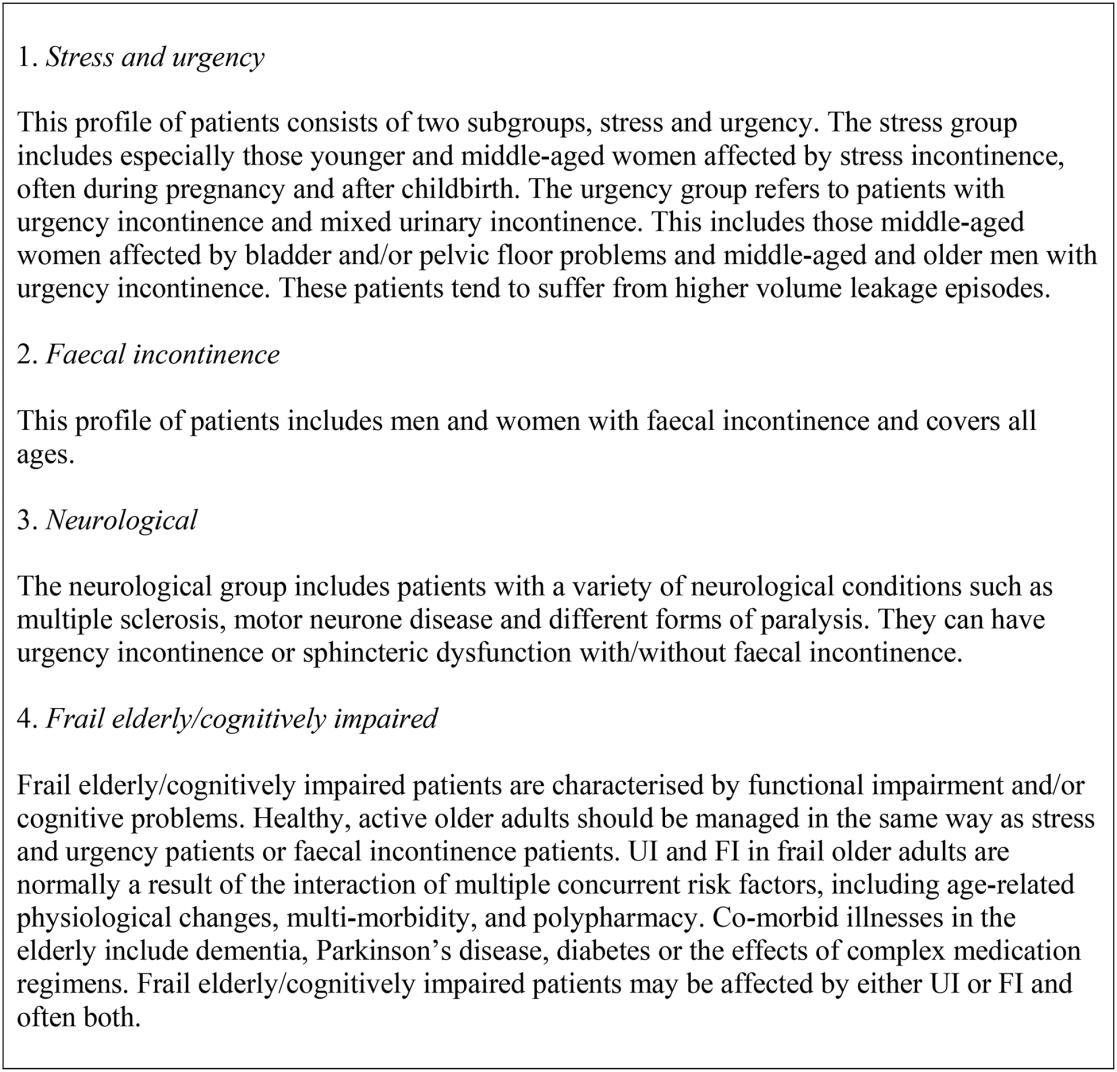
Results of search for articles relating to the organisation of continence care services.

From the combined searches, 4752 studies were returned, of which 312 were selected following review of the abstract. Of these, 101 articles met the inclusion criteria (Figure 5).

### Key themes arising from literature review and moderated panel discussions

The EP considered the results of the literature review and the interview findings as an integrated body of evidence. Discussions concentrated on considering all of the results together regardless of the original source. The resulting themes from the activities are outlined below.

#### Background

**There are wide variations in continence care service delivery across the world:** Factors affecting the delivery of a continence care service were: population demographics and patient characteristics; cultural differences in healthcare seeking and disease recognition; geographical healthcare access; financial healthcare access; maturity and development of existing continence care provision; extent to which services are integrated; economic and regulatory levers available to influence healthcare provision; application of technology in the delivery of care.

#### Continence care is not currently a priority for health system administrators; the recent trend is for a reduction in available resources

Every interviewee agreed that continence care was not a priority for their healthcare system; there was broad agreement that this was a common finding in other countries. Most respondents agreed that if there was a trend in healthcare spending on continence care it was decreasing.

#### Impact

**Incontinence has a significant impact on the lives of both people with incontinence and their caregivers, with serious implications for local health economies in terms of provision of health and social care:** Interviewees and panel members noted the significant impact that incontinence has on the lives of patients and caregivers. Available evidence from the literature confirmed that, compared with other common chronic illnesses, incontinence has a major impact on quality of life.

#### Scope and quality of current services

**Where there is evidence, the best-performing healthcare services are locally-derived and led by motivated individuals with specific interests and skills:** The interviews highlighted isolated examples of good practice in the delivery of continence care. However, the prevailing view of both interviewees and published evidence was that these examples were exceptions, and mostly built on the drive and motivation of interested professionals. It was difficult to differentiate whether the major driver of these successes was the training and background of the healthcare professional or the existence of a defined referral pathway for patients with incontinence.

#### Basic continence care, either in the identification of cases or in the adherence to the clinical evidence base, is often not delivered well by generalist healthcare professionals

Most studies that investigated the ability of family/general physicians and generalist nurses to either detect cases of incontinence or adhere to evidence-based clinical guidelines in the management of incontinence showed poor performance in both areas. A lack of awareness of the impact of the problem on patients, and a lack of knowledge/competence in the management of incontinence were cited as likely causes. Associated with these causes was the low priority placed on incontinence by family/general physicians due to a combination of a lack of incentives, a lack of training (often none received since medical college) and competing priorities on the time of (particularly family) physicians. While generalist nurses (mostly community nurses) were more likely to detect incontinence they had a tendency to rely on containment products rather than recommending first-line behavioural treatment or referral on to a physician/specialist for further management.

#### There is a lack of healthcare professionals trained in continence care, both in terms of generalists with basic training and specialists with more comprehensive training

General training for healthcare professionals commonly included little education on the management of incontinence. Amongst specialist physicians, such as gynaecologists and urologists, relatively few specialise in incontinence compared to the prevalence of the condition. A small, but increasingly significant cohort of nurses has been trained specifically in incontinence. There is a range in the level of qualifications and training amongst these specialised nurses or *nurse specialists.*

### Development of the Care Service Specification

Given the wide variety of healthcare systems and policy contexts, the EP recognised that there was no single model that would be appropriate to all healthcare systems. In order to create an internationally-applicable service specification, a modular approach, aligned to the agreed design principles, was taken which allowed sensitivity to variables in different healthcare localities and the ability to “pick and choose” service elements.

**Figure 6:**
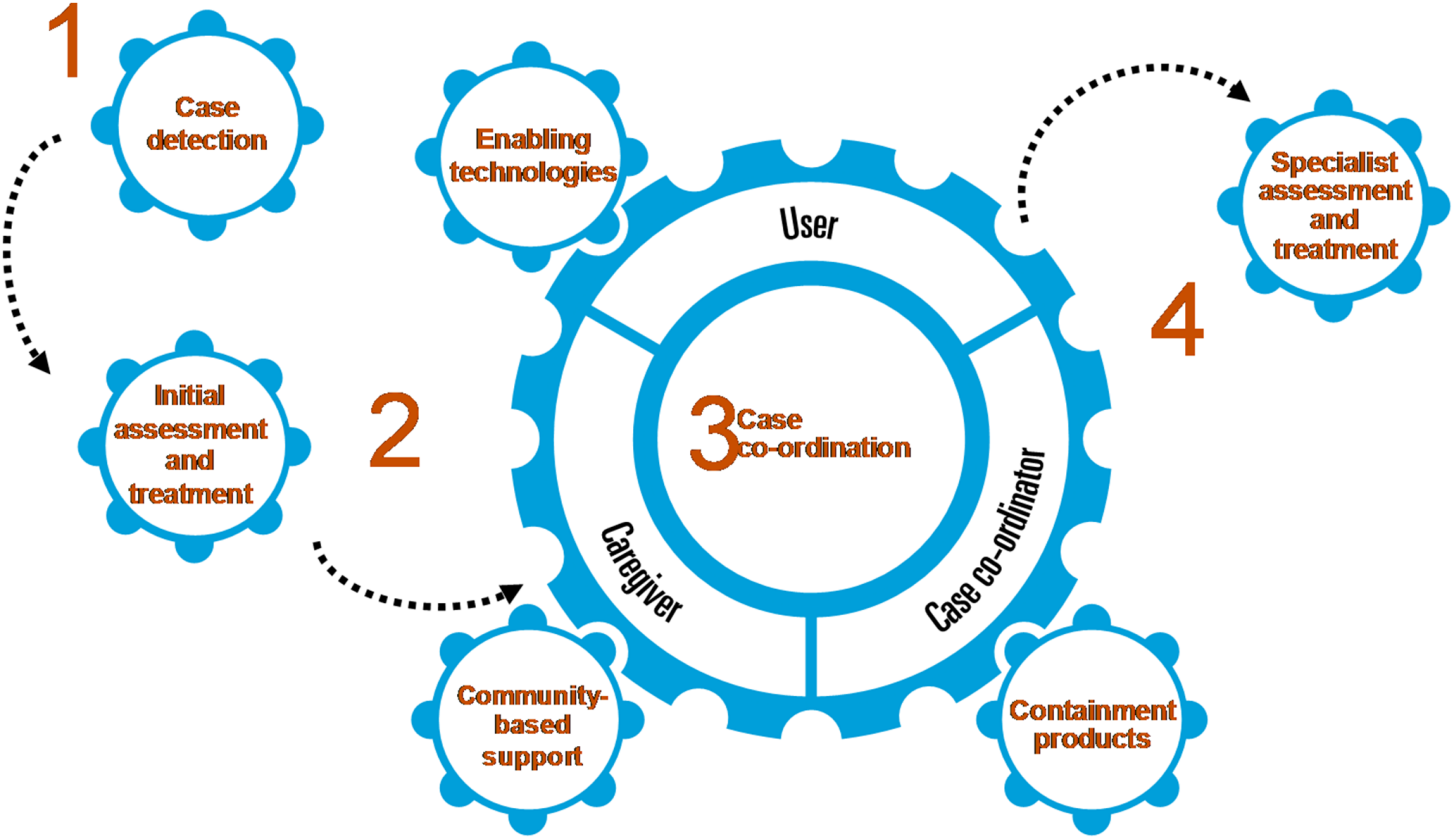
Components of a continence care service.

The modular service specification delineated essential components of a comprehensive continence care service. These were: 1) case detection, 2) initial assessment and treatment, 3) case co-ordination, 4) caregiver support, 5) community-based support, 6) specialist assessment and treatment, 7) use of containment products, and 8) use of technology (Figure 6).

Key recommendations were:

- Ensure ease of access by the establishment of robust referral pathways from detection of incontinence through to appropriate assessment and treatment;
- Shift the responsibility of basic continence care away from primary care physicians to continence nurse specialists in primary care, where available;
- Where continence nurse specialists are unavailable, train existing healthcare professionals such as primary care-based nurse practitioners, community nurses, physician’s assistants, or, in developing countries, local community healthcare workers, to provide evidence-based continence care;
- Where possible, use a case co-ordinator to ensure collaborative working, especially to help delay or prevent admission of patients to permanent care settings; given the general trend to more integrated clinical pathways, in particular concerning patients with multimorbidities, it is necessary to strike a balance between specialisation and holistic case management approaches;
- Promote use of self-management tools and techniques; provision of information on the use of containment products; use of enabling technologies; an emphasis on shared decision-making between healthcare provider and patient/caregiver; and educational campaigns on the nature of the illness and treatment strategies;
- Specialists should be well integrated with other parts of the care pathway. They play a key role in quality governance, training and the dissemination of best practice;
- Use a comprehensive assessment of user, product, and usage-related factors to assess the needs of patients and caregivers with regards to containment products. This process should be standardised, valid and easily reproducible. The final decision regarding choice of product should remain with the end-user: the patient and/or their informal or professional caregiver;
- The use of technology should be integral to the delivery of continence care. Technology should enable self-care and connect patients, caregivers and enable providers to monitor progress and troubleshoot problems;
- *For payers*: in order to provide the highest quality continence care, ensure care standards are incentivised. This can be achieved through stipulating the achievement of targets on certain outcome and operational measures, careful use of quality-related financial incentives, emphasis on clinical governance and optimal pricing that is most strongly correlated to the true cost of providing a service;
- Establish accredited programmes of training for 1) nurses wanting to become continence nurse specialists, and 2) other health or social care professionals such as social workers wishing to improve their competence in delivering continence care.

These recommendations formed the basis of a more comprehensive document setting out how best to deliver continence care, File S1 Optimum service specification complete.

## Discussion

This paper describes the development of a comprehensive service specification for improving continence care services around the world. Based on a systematic literature review and a range of expert interviews, current shortfalls in various health systems were identified to design potential improvements for different patient profiles (Figure 4) through an integrated approach to continence care. An eight component modular service specification was developed. Recognising that medical, social, and economic resources are often unequally available around the world, each of these components can be delivered by different people or through varying channels.

### Robust referral pathways

The route from detection of symptoms through to the receipt of evidence-based care is a critically important path. There are a number of ways in which people may gain access to a service and become patients.

In developed healthcare systems the first contact, if initiated by the patient, is likely to be with the primary care physician [35]. However, primary care physicians often have limited knowledge of established evidence-based clinical guidelines [36], [37], [38]. The implications of this shortfall for the delivery of conservative treatments are discussed later, but one consequence is the lack of awareness of services to which to refer in order to ensure delivery of quality evidence-based care for the patient. Providers of home care are not necessarily better equipped than primary care providers to manage a person with incontinence correctly [39], and similarly may resort to prescribing incontinence pads without adequate clinical assessment.

Healthcare professionals from a variety of disciplines will need to know how to help guide the patient to receive effective treatment. Improving *case detection* is not straightforward and will require robust pathways of referral from *case detection* through to appropriate assessment and treatment.

### The role of continence nurse specialists

*Initial assessment and treatment* may be optimally enacted by a dedicated local nurse-led continence service, which may be situated either in the community or could be co-located with a specialist clinic.

Nurses with appropriate training are capable of managing and treating incontinence more effectively than primary care physicians [27], [40], [41], [42]. They are also able to triage and independently manage a significant proportion of patients [43], [44], especially geriatric nurses who are managing other chronic diseases [45] There is evidence that patients appreciate the communication skills and comprehensive continence care provided by nurses [46], [47].

There is no conclusive evidence that the direct costs of providing a nurse-led service are more or less than a physician-led service [27], [41], [48], [49]. The cost-effectiveness of nurses versus physicians depends on a number of factors such as: the salary differential between nurses and doctors, the differences in productivity, and outcomes achieved.

The majority of evidence for the effectiveness of nurse specialists in continence care originates from the United Kingdom, the United States, the Netherlands, Canada, Australia and Sweden (urotherapist model) which limits the generalisability of the findings. We can only recommend this model in countries where nurses have the funding, support and training programmes required to substitute for physicians, otherwise initial assessment and treatment can be performed by trained local community health workers.

### The role of the case-coordinator

Self-reported ‘integrated’ continence services have been shown to provide higher-quality care [50] and have demonstrated positive outcomes in other chronic diseases. for example in patients with diabetes and depression [51]. A simple disease management approach for chronic obstructive pulmonary disease involving the use of a case manager significantly reduced emergency department visits and hospital admissions [52]. A case co-ordinator is able to provide a single point of contact that can co-ordinate the multiple agencies involved in providing continence care.

When the patient is suffering from a complex primary illness, it is important to avoid duplication of care [53]. For example, elderly patients will often have a complex array of conditions with associated health and social care needs that require a wider lens than that provided by a continence case co-ordinator. In these circumstances, the case co-ordinator will take a more facilitative and advisory role, filling in the gaps in care and care co-ordination where they exist. The role would then be one orientated more around providing input on appropriate health and social care interventions to other specialist nurses or acting as a gate-keeper/triage service to each of the available components of the continence service [43], [54].

### The role of specialists

According to international clinical guidelines, specialists in continence care should be focused on those with severe symptoms or who are unresponsive to conservative treatment strategies, complex patients and those with a clear indication for more invasive treatments [55]. For example, patients with neurological incontinence, those with FI and frail elderly incontinent patients each have their own distinctive profiles, health and social care needs.

While providers of *specialist assessment and treatment* should probably be separate from those providing *initial assessment and treatment* it is important that there is operational integration with other components of the service.

Specialists also have an important role in facilitating the sharing of best practice and training as well as in quality governance. The integration of an academic centre with a community-based service has proven to be challenging in the past with barriers to overcome regarding funding sources and existing healthcare infrastructure [56].

### Containment products

The use of containment products is an important element in the management of incontinence. Containment products should be provided during the treatment process or when cure is not possible through a strategy of ‘contained incontinence’.

Where provided, patients are often left wanting by prescriptions that are not sensitive to clinical need, and are insensitive to the needs of patients [57]. The international standard for the evaluation of containment products (ISO 15621∶2011) recommends consideration of a number of factors separated into three categories: user-related factors such as quality of life, independence, needs; usage-related factors such as care setting, needs of caregiver, ease of handling of product in relation to care setting, total costs; and product-related factors such as freedom from leakage, skin health, comfort.

Where continence care services also fund the provision of products, the introduction of a standardised assessment tool should reduce variation in provision. The needs of each patient should be reassessed periodically to ensure appropriate consumption of products, reducing the need for rationing of products.

In order for patients and formal and informal caregivers to make informed decisions regarding containment products, patients should be provided with information on the types of products available and factors to consider when making the choice [58]. Where available this should be supplemented with samples to test the best possible product or combinations to suit patients and their caregivers’ needs.

### The role of technology

Technology can potentially enhance current models of continence care. While some population groups are still disadvantaged in access and skills with which to use ICT applications, further development will depend on patients’ acceptance, cognitive abilities and affordability. There is potential for collaborative development of several areas which may enhance care delivery.

The use of telehealth services can connect healthcare professionals to patients with limited physical access to services e.g. those who live in rural areas. Potential uses include video consultations and remote monitoring. Telehealth could fill the gaps where resources and manpower are lacking. The use of mobile SMS text messaging for prevention, surveillance, self-management and compliance in developing countries is well established [59] and there is no obvious reason why such a medium could not work well in developed healthcare systems.

There are a number of uses and roles for the internet in the delivery of continence care. Internet-based treatments that include e-mail support and cognitive behavioural exercises have been shown to be effective. Mobile applications that aim to deliver the same treatments are currently under investigation [60]. Other tools and arrangements based on the internet include self-assessment tools [61], facilitated patient networks [62], and facilitated professional networks [63].

These tools also provide a role in continence promotion as patients and healthcare professionals communicate their experiences of receiving and providing care. An example of a professional network based around another chronic condition is the Netherlands’ *ParkinsonNet*, an ICT-enabled community-based network of physiotherapists that is able to provide higher quality, lower cost care compared with usual care [63]. Well-developed patient networks may be a valuable source of data on treatment effectiveness and side effects.

Lastly, advances in the sophistication of electronic medical records could potentially enhance case detection and evidence-based management and treatment [64].

### Continence education and training for healthcare professionals

Where there is a paucity of continence nurse specialists, it will be necessary to train existing professionals, especially those who currently see people with incontinence, and those who are well placed to deliver continence care. Healthcare professionals are capable of learning a variety of skills, often outside of their usual domain. For example, in the Netherlands, primary care-based nurse practitioners (called Praktijk Ondersteuners, or POHs) have taken on an increasing role in the management of UI in some regions [65]. In the United States, advanced practice providers (nurse practitioners, physician assistants) have acquired additional education and training in providing continence services [66]. In rural Bangladesh, research is currently being undertaken to investigate the role of village “paramedics” or other trained healthcare workers to carry out basic continence care (unpublished data).

Where there are no professionals effectively managing incontinence, we recommend a focus on training and educating primary care physicians, primary care-based nurse practitioners, village ‘paramedics’ or other primary care/community-based healthcare professionals to enhance competency to perform the role [36], [67], [68], [69].

For nurse specialists to play a more prominent role in continence care requires a substantial programme of training of existing nurses, even in systems where continence nurse specialists are plentiful. We recommend the establishment of a certificate in continence care nursing to provide assurance on quality of practice of new and existing continence nurse specialists, and nurses with a special interest [3]. Such a training programme will require a substantial investment on the part of those organisations responsible for professional training and will likely take several years to bring continence nurse numbers to the required level. A significant part of training will necessarily involve “on-the-job” experience, and this will require educational institutions to work effectively with provider organisations.

The most significant barrier to implementing this model may occur in healthcare systems where it is not usual for nurses to take on additional clinical responsibilities. In these systems it may be necessary to focus on educating and training other appropriate staff.

In almost all healthcare systems, there will need to be a short-term focus on the training of existing healthcare professionals including generalist and specialist physicians, specialist physiotherapists, generalist nurses and existing continence care specialists. Existing ‘nursing champions’ should also be empowered to help diffuse best practice within existing generalist nurses [22], [70].

### Implications for payers

A recurring theme through our interviews was the low priority of continence care in a time of restricted financial resources. For example, in a recent survey in the UK, many respondents cited the lack of incentives for providers of continence care services to expand treatment [57].

Payment incentives have been important in improving the quality of care in health care, for example through the use of the Quality and Outcomes Framework in the reimbursement of UK primary care physicians and the deployment of performance-related payments in the Geisinger Health System [71]. A lack of financial incentives for primary care physicians may be one of a number of factors behind the under-performance of primary care physicians to provide evidence-based continence care. In more market-based healthcare systems, reimbursement that is most closely aligned with the actual cost of providing care minimises adverse incentives that lead to market failures [72]. The ideal payment system would appear to be one which identifies and rewards ‘value creation’ in healthcare: the achievement of healthcare benefits (outcomes) per unit cost [73]. Payment incentives are, however, not a panacea, and must be changed in co-ordination with health-system financing, regulation, organisation of healthcare and behaviour [74].

#### Limitations of the study

It is challenging to create a service specification that is internationally applicable given the diverse nature of healthcare systems and the varying states of their finances, healthcare infrastructure, workforce capability and regulatory environment. The study addresses this difficulty by firstly conducting a systematic literature review including conventional datasets, the grey literature and expert opinion from four broadly representative healthcare systems and secondly by reducing the optimal continence service to its core components, giving a framework from which to organise continence services, regardless of resources. However, not all recommendations will be immediately relevant and will require the user’s discretion in tailoring them to specific country needs. To deliver a high-quality continence care service requires the optimisation of a number of components. Successful implementation will require sensitivity to the needs of variety of stakeholders including payers, providers, professionals, and patients and their caregivers.

There are three limitations to this study. First, in the literature review non-English references were excluded. It is therefore possible that important findings and insights which could have informed the development of the service specification were missed. Second, there is a paucity of literature about continence care in developing countries. We were able to mitigate this limitation to some extent by collecting primary research from experts, especially in India, but nevertheless the balance of evidence largely came from more developed countries; the inclusion of a clinician majority in developing countries may in itself had led to unavoidable bias Finally, the study did not consider the financial implications of the new service specifications. We plan to address this limitation in a follow-up study.

## Conclusions

This study, using robust methods, has 1) defined practice gaps in the provision of continence services and 2) described eight core components of a service specification for incontinence that commissioners and payers of health and social care could consider using to provide high-quality continence care. A shift away from physician-provided care towards a community-delivered, nurse-led model appears to be associated with clinical and cost-effective care for people with bladder and bowel incontinence. Where nurse specialists are not available, other healthcare workers can be trained to identify cases and provide initial assessment and management. In resource-poor areas, self-management and innovative uses of technology to support this may prove to reduce the burden of care. Evidence suggests that services should be integrated across primary and secondary care wherever possible to ensure a seamless transition to more complex care, should this be needed. Quality outcomes should, where possible, be monitored and incentives to achieve these put in place by health services managers.

## Supporting Information

Checklist S1 **PRISMA document.**
(PDF)

File S1 **Optimum service specification complete.**
(PDF)

## References

[1] Milsom I, Altman D, Cartwright R, Lapitan MC, Nelson R, et al. (2013) Epidemiology of Urinary Incontinence (UI) and Lower Urinary Tract Symptoms (LUTS), Pelvic Organ Prolapse (POP) and Anal Incontinence (AI). Incontinence 5th Edition: 15–108.

[2] All Party Parliamentary Group for Continence Care (2011) Cost-effective Commissioning For Continence Care APPG for Continence Care.

[3] Newman DK, Buckley B, Gordon D, Griebling TL, Petty LE, et al. (2013) Continence Promotion, Education & Primary Prevention. Incontinence 5th Edition.

[4] Lee YS, Lee KS, Jung JH, Han DH, Oh SJ, et al (2011) Prevalence of overactive bladder, urinary incontinence, and lower urinary tract symptoms: results of Korean EPIC study. World J Urol 29: 185–190. 19898824 10.1007/s00345-009-0490-1

[5] Ebbesen MH, Hunskaar S, Rortveit G, Hannestad YS (2013) Prevalence, incidence and remission of urinary incontinence in women: longitudinal data from the Norwegian HUNT study (EPINCONT). BMC Urol 13: 27. 23721491 10.1186/1471-2490-13-27 PMC3674916

[6] Stewart WF, Van Rooyen JB, Cundiff GW, Abrams P, Herzog AR, et al (2003) Prevalence and burden of overactive bladder in the United States. World J Urol 20: 327–336. 12811491 10.1007/s00345-002-0301-4

[7] Coyne KS, Sexton CC, Thompson CL, Milsom I, Irwin D, et al (2009) The prevalence of lower urinary tract symptoms (LUTS) in the USA, the UK and Sweden: results from the Epidemiology of LUTS (EpiLUTS) study. BJU Int 104: 352–360. 19281467 10.1111/j.1464-410X.2009.08427.x

[8] Tennstedt SL, Link CL, Steers WD, McKinlay JB (2008) Prevalence of and risk factors for urine leakage in a racially and ethnically diverse population of adults: the Boston Area Community Health (BACH) Survey. Am J Epidemiol 167: 390–399. 18182376 10.1093/aje/kwm356

[9] NICE (2007) Faecal incontinence: the management of faecal incontinence in adults. NICE clinical guideline 49.

[10] Grimby A, Milsom I, Molander U, Wiklund I, Ekelund P (1993) The influence of urinary incontinence on the quality of life of elderly women. Age Ageing 22: 82–89. 8470564 10.1093/ageing/22.2.82

[11] Schultz SE, Kopec JA (2003) Impact of chronic conditions. Health Rep 14: 41–53. 14608795

[12] Horng SS, Huang N, Wu SI, Fang YT, Chou YJ, et al (2013) The epidemiology of urinary incontinence and it’s influence on quality of life in Taiwanese middle-aged women. Neurourol Urodyn 32: 371–376. 22972439 10.1002/nau.22302

[13] Thomas P, Ingrand P, Lalloue F, Hazif-Thomas C, Billon R, et al (2004) Reasons of informal caregivers for institutionalizing dementia patients previously living at home: the Pixel study. Int J Geriatr Psychiatry 19: 127–135. 14758578 10.1002/gps.1039

[14] van der Veen R, Mak S, Bodnarova B, Selestiakova K, Hanson E, et al (2011) Quality of life of carers managing incontinence in Europe. http://www.eurocarers.org/userfiles/file/research/Europe_Carers_managing_incontinence_2011.pdf (accessed 6.7.2014).

[15] Appleby I, Whitlam G, Wakefield N (2013) Incontinence in Australia. Australian Institute of Health and Welfare.

[16] Noimark D, Steventon N, Wagg A (2009) A qualitative study of the impact of caring for a person with urinary incontinence (Abstract) 39th Annual Meeting of the International Continence Society San Francisco, USA 29 September – 3 October, 2009. Neurourology and Urodynamics 28: 567–935.

[17] Gotoh M, Matsukawa Y, Yoshikawa Y, Funahashi Y, Kato M, et al (2009) Impact of urinary incontinence on the psychological burden of family caregivers. Neurourology and Urodynamics 28: 492–496. 19090589 10.1002/nau.20675

[18] Tarricone R (2010) Economics of Incontinence; Global Forum for Incontinence Prague.

[19] Wilson L, Brown JS, Shin GP, Luc KO, Subak LL (2001) Annual direct cost of urinary incontinence. Obstet Gynecol 98: 398–406. 11530119 10.1016/s0029-7844(01)01464-8

[20] Moore K, Wagner TH, Subak LL, De Wachter S, Dudding T, et al. (2013) Economics of Urinary and Faecal Incontinence, and Prolapse 2012. Incontinence 5th Edition. 1831–1862.

[21] Royal College of Physicians (2012) National audit of continence care (NACC). Available: http://www.hqip.org.uk/assets/NCAPOP-Library/NCAPOP-2012-13/Continence-pilot-audit-evaluation-report-published-Aug-2012.pdf. Accessed 2014 Jun 7.

[22] Paterson J (2006) Consultation, Consensus and Commitment to Guidelines for Inclusion of Continence into Undergraduate Nursing and Midwifery Curricula. Submitted to The Commonwealth Department of Health and Ageing. Available: http://www.researchgate.net/publication/29465616_Consultation_Consensus_and_Commitment_to_Guidelines_for_Inclusion_of_Continence_into_Undergraduate_Nursing_and_Midwifery_Curricula. Accessed 2014 Jun 7.

[23] McClurg D, Cheater FM, Eustice S, Burke J, Jamieson K, et al (2013) A multi-professional UK wide survey of undergraduate continence education. Neurourol Urodyn 32: 224–229. 22847225 10.1002/nau.22284

[24] Imamura M, Abrams P, Bain C, Buckley B, Cardozo L, et al. (2010) Systematic review and economic modelling of the effectiveness and cost-effectiveness of non-surgical treatments for women with stress urinary incontinence. Health Technol Assess 14: 1–188, iii–iv. 10.3310/hta14400 20738930

[25] Ho MT, Eastwood A, Kuteesa W, Short A, Moore K (2012) Incontinence after childbearing: Long-term analysis of direct costs of conservative and surgical therapy. The Australian and New Zealand Continence Journal 18.

[26] National Collaborating Centre for Women’s and Children’s Health (2006) Urinary incontinence: the management of urinary incontinence in women (Full Guideline). London (UK): Royal College of Obstetricians and Gynaecologists (RCOG).

[27] Williams KS, Assassa RP, Cooper NJ, Turner DA, Shaw C, et al (2005) Clinical and cost-effectiveness of a new nurse-led continence service: a randomised controlled trial. Br J Gen Pract 55: 696–703. 16176737 PMC1464065

[28] Wagg A, Mian S, Lowe D, Potter J, Pearson M (2005) National audit of continence care for older people: results of a pilot study*. J Eval Clin Pract 11: 525–532. 16364105 10.1111/j.1365-2753.2005.00570.x

[29] Gawande A (2009) The Cost Conundrum. What a Texas town can teach us about health care. The New Yorker. Available: http://www.newyorker.com/reporting/2009/06/01/090601fa_fact_gawande. Accessed 2014 Jun 7.

[30] Ginsburg PB (2012) Fee-for-service will remain a feature of major payment reforms, requiring more changes in Medicare physician payment. Health Aff (Millwood) 31: 1977–1983. 22949446 10.1377/hlthaff.2012.0350

[31] Krissovich M (1998) The financial side of continence promotion. Geriatr Nurs 19: 91–94. 9611507 10.1016/s0197-4572(98)90045-3

[32] Sirls LT, Richter HE, Litman HJ, Kenton K, Lemack GE, et al (2013) The Effect of Urodynamic Testing on Clinical Diagnosis, Treatment Plan and Outcomes in Women Undergoing Stress Urinary Incontinence Surgery. The Journal of urology 189: 204–209. 22982425 10.1016/j.juro.2012.09.050 PMC4363108

[33] Gemmill R, Wells A (2010) Promotion of urinary continence worldwide. Urol Nurs 30: 336–340. 21261193

[34] NICE (2013) Interim methods guide for developing service guidance. Available: http://publications.nice.org.uk/interim-methods-guide-for-developing-service-guidance-pmg8. Accessed 2014 Jun 7. 28230952

[35] O’Donnell M, Viktrup L, Hunskaar S (2007) The role of general practitioners in the initial management of women with urinary incontinence in France, Germany, Spain and the UK. Eur J Gen Pract 13: 20–26. 17366290 10.1080/14017430601049381

[36] McFall S, Yerkes AM, Bernard M, LeRud T (1997) Evaluation and treatment of urinary incontinence. Report of a physician survey. Arch Fam Med 6: 114–119. 9075444 10.1001/archfami.6.2.114

[37] Albers-Heitner P, Berghmans B, Nieman F, Lagro-Janssen T, Winkens R (2008) Adherence to professional guidelines for patients with urinary incontinence by general practitioners: a cross-sectional study. J Eval Clin Pract 14: 807–811. 18462277 10.1111/j.1365-2753.2007.00925.x

[38] Swanson JG, Skelly J, Hutchison B, Kaczorowski J (2002) Urinary incontinence in Canada. National survey of family physicians’ knowledge, attitudes, and practices. Can Fam Physician 48: 86–92. 11852616 PMC2213918

[39] Du Moulin MF, Hamers JP, Ambergen AW, Halfens RJ (2009) Urinary incontinence in older adults receiving home care diagnosis and strategies. Scand J Caring Sci 23: 222–230. 19645801 10.1111/j.1471-6712.2008.00610.x

[40] Borrie MJ, Bawden M, Speechley M, Kloseck M (2002) Interventions led by nurse continence advisers in the management of urinary incontinence: a randomized controlled trial. CMAJ 166: 1267–1273. 12041843 PMC111077

[41] Moore KH, O’Sullivan RJ, Simons A, Prashar S, Anderson P, et al (2003) Randomised controlled trial of nurse continence advisor therapy compared with standard urogynaecology regimen for conservative incontinence treatment: efficacy, costs and two year follow up. BJOG 110: 649–657. 12842055

[42] Farrell SA, Scott TA, Farrell KA, Irving L, Foren J, et al (2009) Two models for delivery of women’s continence care: the step-wise continence team versus the traditional medical model. J Obstet Gynaecol Can 31: 247–253. 19416571 10.1016/S1701-2163(16)34123-8

[43] Oliver R, Thakar R, Sultan AH, Phillimore A (2009) Urogynecology triage clinic: a model of healthcare delivery. Int Urogynecol J Pelvic Floor Dysfunct 20: 913–917. 19475330 10.1007/s00192-009-0878-x

[44] Matharu GS, Assassa RP, Williams KS, Donaldson MK, Matthews RJ, et al (2004) Continence nurse treatment of women’s urinary symptoms. Br J Nurs 13: 140–143. 14997075 10.12968/bjon.2004.13.3.12110

[45] Reuben DB, Ganz DA, Roth CP, McCreath HE, Ramirez KD, et al (2013) Effect of nurse practitioner comanagement on the care of geriatric conditions. J Am Geriatr Soc 61: 857–867. 23772723 10.1111/jgs.12268 PMC3694740

[46] Albers-Heitner P, Winkens R, Berghmans B, Joore M, Nieman F, et al (2013) Consumer satisfaction among patients and their general practitioners about involving nurse specialists in primary care for patients with urinary incontinence. Scand J Caring Sci 27: 253–259. 22651242 10.1111/j.1471-6712.2012.01023.x

[47] Shaw C, Williams KS, Assassa RP (2000) Patients’ views of a new nurse-led continence service. J Clin Nurs 9: 574–582. 11261138 10.1046/j.1365-2702.2000.00414.x

[48] Laurant M, Reeves D, Hermens R, Braspenning J, Grol R, et al. (2005) Substitution of doctors by nurses in primary care. Cochrane Database Syst Rev: CD001271. 10.1002/14651858.CD001271.pub2 15846614

[49] Albers-Heitner CP, Joore MA, Winkens RA, Lagro-Janssen AL, Severens JL, et al (2012) Cost-effectiveness of involving nurse specialists for adult patients with urinary incontinence in primary care compared to care-as-usual: an economic evaluation alongside a pragmatic randomized controlled trial. Neurourol Urodyn 31: 526–534. 22275126 10.1002/nau.21204

[50] Wagg A, Lowe D, Peel P, Potter J (2009) Do self-reported ‘integrated’ continence services provide high-quality continence care? Age Ageing 38: 730–733. 19793925 10.1093/ageing/afp177

[51] Katon WJ, Lin EH, Von Korff M, Ciechanowski P, Ludman EJ, et al (2010) Collaborative care for patients with depression and chronic illnesses. N Engl J Med 363: 2611–2620. 21190455 10.1056/NEJMoa1003955 PMC3312811

[52] Rice KL, Dewan N, Bloomfield HE, Grill J, Schult TM, et al (2010) Disease management program for chronic obstructive pulmonary disease: a randomized controlled trial. Am J Respir Crit Care Med 182: 890–896. 20075385 10.1164/rccm.200910-1579OC

[53] Lugtenberg M, Burgers JS, Clancy C, Westert GP, Schneider EC (2011) Current guidelines have limited applicability to patients with comorbid conditions: a systematic analysis of evidence-based guidelines. PLoS One 6: e25987. 22028802 10.1371/journal.pone.0025987 PMC3197602

[54] Georgiou EX, Domoney C, Marsh S, Stafford M (2011) Streamlining outpatient urogynaecology: a novel approach. J Obstet Gynaecol 31: 156–163. 21281034 10.3109/01443615.2010.541569

[55] Abrams P, Andersson KE, Artibani W, Birder L, Bliss D, et al. (2013) Fifth International Consultation on Incontinence Recommendations of the International Scientific Committee: Evaluation and treatment of urinary incontinence, pelvic organ prolapse, and fecal incontinence. Incontinence 5th Edition: 1895–1956.

[56] Stothers L, Wilkie D, Lieblich P, Wilson P (2008) Developing a continence care centre using an urban/academic model of continence care. Can J Urol 15: 4084–4090. 18570714

[57] Harari D, Rogers J, Eustice S, Colley W (2013) Continence Care Services England 2013. Survey Report. All Party Parliamentary Group for Continence Care. Available: http://www.appgcontinence.org.uk/pdfs/Continence%20Care%20Services%20England%20Report%202013.pdf. Accessed 2014 Jun 7.

[58] Paterson J, Dunn S, Kowanko I, van Loon A, Stein I, et al (2003) Selection of continence products: perspectives of people who have incontinence and their carers. Disabil Rehabil 25: 955–963. 12851083 10.1080/096382809210142211

[59] Deglise C, Suggs LS, Odermatt P (2012) SMS for disease control in developing countries: a systematic review of mobile health applications. J Telemed Telecare 18: 273–281. 22826375 10.1258/jtt.2012.110810

[60] Stephen K, Rodger G, Cumming G (2012) Attitudes to apps for continence. International Congress on Telehealth and Telecare. London, UK.

[61] de Bruin M (2013) A new ehealth service for women with urinary incontinence: an online diagnostic expert program combined with a consult at home by a continence nurse. International Continence Society 2013.

[62] Wicks P, Stamford J, Grootenhuis MA, Haverman L, Ahmed S (2013) Innovations in e-health. Qual Life Res. 23(1): 195–203. 10.1007/s11136-013-0458-x PMC3929022 23852096

[63] Munneke M, Nijkrake MJ, Keus SH, Kwakkel G, Berendse HW, et al (2010) Efficacy of community-based physiotherapy networks for patients with Parkinson’s disease: a cluster-randomised trial. Lancet Neurol 9: 46–54. 19959398 10.1016/S1474-4422(09)70327-8

[64] Hsiao CJ, Marsteller JA, Simon AE (2013) Electronic Medical Record Features and Seven Quality of Care Measures in Physician Offices. Am J Med Qual. 29(1): 44–52. 10.1177/1062860613483870 23610232

[65] Albers-Heitner P (2011) WHO CARES? Studying various aspects of involving nurse specialists in primary care for urinary incontinence. University of Maastricht 2011: ISBN 978 94 6159 090 9.

[66] Newman D (2006) The roles of the continence nurse specialists. In Cardozo L, Staskin D (Eds.): Textbook of Female Urology and Urogynecology, 2nd Edition. 91–98 p.

[67] Albers-Heitner P, Berghmans B, Joore M, Lagro-Janssen T, Severens J, et al (2008) The effects of involving a nurse practitioner in primary care for adult patients with urinary incontinence: the PromoCon study (Promoting Continence). BMC Health Serv Res 8: 84. 18412964 10.1186/1472-6963-8-84 PMC2386786

[68] Shaw C, Atwell C, Wood F, Brittain K, Williams K (2007) A qualitative study of the assessment and treatment of incontinence in primary care. Fam Pract 24: 461–467. 17670805 10.1093/fampra/cmm041

[69] Viktrup L, Moller LA (2004) The handling of urinary incontinence in Danish general practices after distribution of guidelines and voiding diary reimbursement: an observational study. BMC Fam Pract 5: 13. 15225353 10.1186/1471-2296-5-13 PMC459219

[70] Ploeg J, Skelly J, Rowan M, Edwards N, Davies B, et al (2010) The role of nursing best practice champions in diffusing practice guidelines: a mixed methods study. Worldviews Evid Based Nurs 7: 238–251. 20880009 10.1111/j.1741-6787.2010.00202.x

[71] Berg M, Ikkersheim D, Schellevis J, Edwards N, Britnell M (2012) Contracting value: Shifting paradigms. KPMG International. http://www.kpmg.com/Global/en/IssuesAndInsights/ArticlesPublications/contracting-value/Documents/contracting-value-v7.pdf (acccessed 6.7.2014).

[72] Kaplan RS, Porter ME (2011) How to solve the cost crisis in health care. Harv Bus Rev 89: 46–52, 54, 56–61 passim. 21939127

[73] Porter ME (2009) A strategy for health care reform–toward a value-based system. N Engl J Med 361: 109–112. 19494209 10.1056/NEJMp0904131

[74] Roberts M, Hsiao W, Berman P, Reich M (2008) Getting Health Reform Right: A Guide to Improving Performance and Equity. Oxford University Press. Oxford UK. ISBN: 9780195371505.

