# Supplementary material for: Developing an Internationally-Applicable Service Specification for Continence Care: Systematic Review, Evidence Synthesis and Expert Consensus
Source: PLoS One. 2014 Aug 14;9(8):e104129. doi: 10.1371/journal.pone.0104129 (PMC4133406; doi:10.1371/journal.pone.0104129)
Supplement: File S1 — Optimum service specification complete. (PDF) [file pone.0104129.s002.pdf]

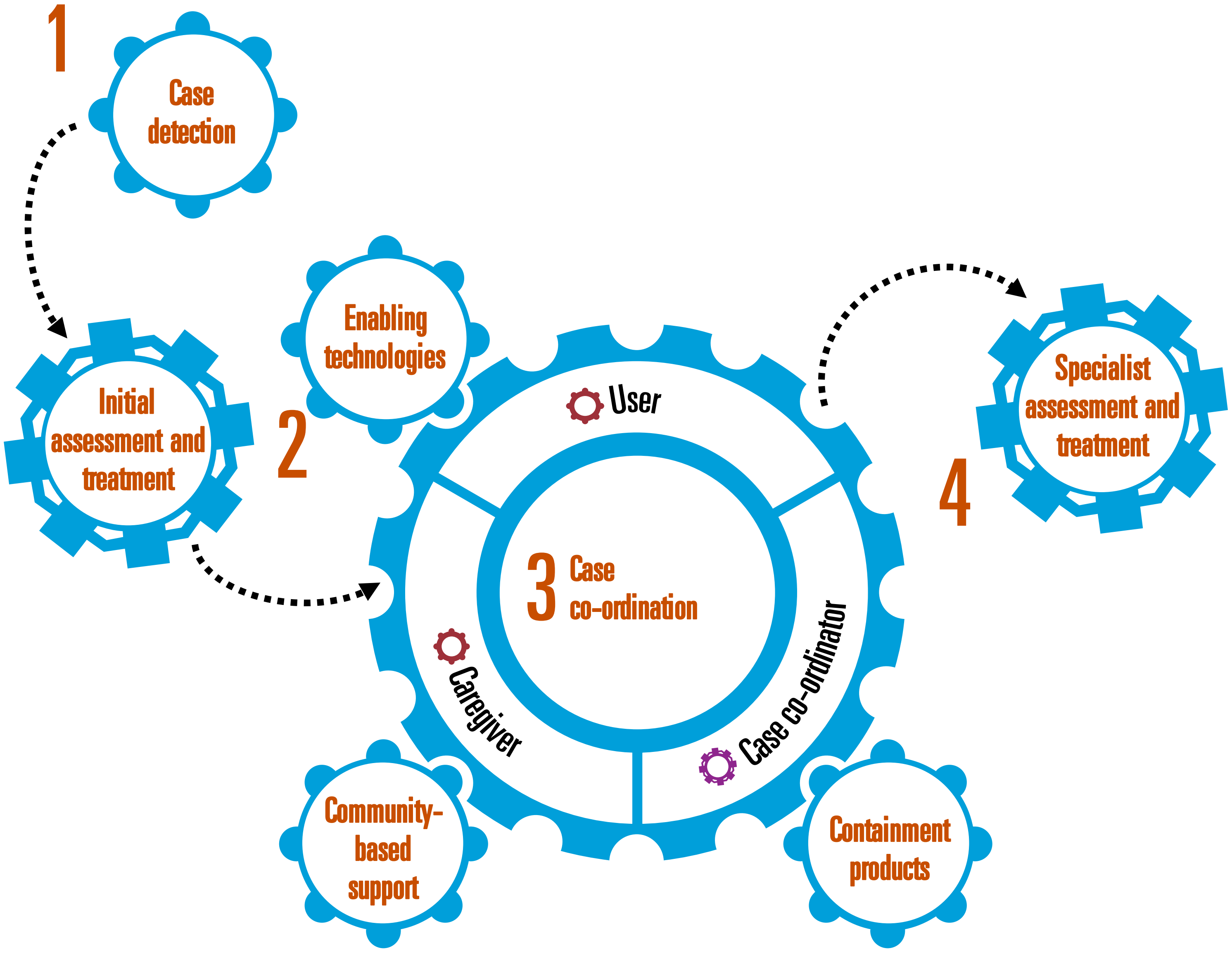

Optimum Continence Service Specification

Expert panel statement

This expert panel was convened to consider how we can deliver continence care in a way that can appropriately address the level of suffering and inconvenience experienced by people with incontinence across the world.

It was ambitious to identify a service specification that can be used worldwide: our approach has been to keep to accredited NICE guidance on the creation of service specifications.

We have placed a priority on achievement of best value for the patient and best value for the commissioner and/or payer of services. This means procuring the optimum healthcare benefit for the cost that the patient and commissioner/payer are willing to take on. This could imply the need for investing in services in the present, to save costs in the future.

We intend for this service specification to provide an evidence-based blueprint for payers, providers and clinical professionals on how best to procure, organise and deliver a continence care service. At the very least, this document should provide a useful reference point for discussion and help to promote the issue of incontinence around the world.

Adrian Wagg

Diane Newman

Paul van Houten

Kai Leichsenring

Acknowledgements

KPMG for research and writing assistance in the development and production of this guide

Initiated and Supported by

SCA

Contents

**1**	**Executive summary**	**5**
**2**	**Introduction**	**7**
2.1	Introduction and context	7
2.2	Our approach	7
**3**	**Impact of the problem**	**11**
3.1	Definition and prevalence	11
3.2	Health economic impact	13
3.3	Current problems in service delivery	14
**4**	**Organisation of continence services**	**16**
4.1	No single optimum model for delivery	16
4.2	Integrated continence care	16
4.3	Components of a continence care service	17
**5**	**Real world applications**	**28**
5.1	Local applications of a continence care service	28
5.2	Patient profiles	39
**6**	**Measuring quality and service performance**	**44**
6.1	Adding value: a focus on outcomes	44
6.2	How to use outcome indicators	44
6.3	Performance measures	44
**7**	**Implementation considerations**	**46**
**8**	**Continence nurse specialist profiles**	**48**
**9**	**Glossary**	**52**
**10**	**References**	**55**
**Appendix 1**	**Members of the expert panel**	**60**
**Appendix 2**	**Clinical guidelines by country**	**61**

1 Executive summary

Global demographic, lifestyle and clinical trends suggest the incidence of those suffering from incontinence (both urinary and faecal) is likely to continue to rise sharply in the coming years. In many countries of the world, patient and caregiver expectations of the availability and quality of continence care continue to increase. In parallel, the increased need to constrain health and social care spending has forced payers to reconsider the overall structure and extent of provision in health and social care systems. Yet, at the organisational level, there is limited evidence to provide guidance to payers and providers about how care might best be configured to deliver established, guideline-compliant, high-quality patient care at either the same, or lower, overall cost.

Against this backdrop an expert panel from a wide range of disciplines and geographies was convened. Its aim was to define, as far as was possible, an 'optimum' service specification for continence care for community-dwelling adults based on best available evidence. Furthermore, given incontinence's global prevalence, the panel aimed to design a specification that was able to take account of local variations in practice, resource and culture. With that in mind, the panel collected and considered evidence from a range of sources including published peer-reviewed journal articles, 'grey' literature and interviews with key opinion leaders, patient groups and payers around the world.

Key findings:

Our key findings in researching this service specification include:

- incontinence is a healthcare issue with significant impact on the lives of both people suffering from incontinence and their caregivers, with serious implications for local health economies in terms of provision of health and social care;
- continence care is not currently a priority for health system administrators; the recent trend is for a reduction in the resources made available to provide continence care;
- where there is evidence, the best-performing healthcare services are frequently locally-derived and led by motivated individuals with specific interests and skills;
- basic continence care is often not delivered well by generalist healthcare professionals either in the identification of cases or in the adherence to evidence-based recommendations;
- there is a lack of healthcare professionals trained in continence care, both in terms of generalists with basic training and specialists with more comprehensive training;
- initial assessment and treatment is optimally enacted by a dedicated local nurse-led continence service, situated either in the community or adjacent to a specialist clinic;
- the interaction of health and social care needs in people with continence problems is often overlooked, with significant negative implications for the overall well-being of both sufferer and caregiver;
- the use of containment products is often inconsistent and insensitive to the holistic needs of patients and caregivers;
- there is a lack of emphasis on self-management, which should be used to empower sufferers and reduce costs in the longer term. In particular, advancements in technology have not been used optimally to enhance standard care.

Key recommendations:

Using consensus methodology, the expert group concluded that continence services need to be redefined to improve the identification, assessment and treatment of incontinence. In this document therefore, we have presented the essential core components of an effective continence service.

Our key recommendations on how best to deliver these core components are:

- ensure ease of access by the establishment of robust referral pathways from detection of incontinence through to appropriate assessment and treatment;
- shift the responsibility of basic continence care away from primary care physicians to continence nurse specialists in primary care, where available;

  Where continence nurse specialists are unavailable, train existing healthcare professionals such as primary care-based nurse practitioners, community nurses, physician's assistants, or, in developing countries, local community healthcare workers, to provide evidence-based continence care;
- where possible, use a case co-ordinator to ensure collaborative working, especially to help delay or prevent admission of patients to permanent care settings; given the general trend to more integrated clinical pathways, in particular concerning patients with multiple morbidities, it is necessary to strike a balance between specialisation and holistic case management approaches;
- promote use of self-management tools and techniques; provision of information on the use of containment products; use of enabling technologies; an emphasis on shared decision-making between healthcare provider and patient/caregiver; and educational campaigns on the nature of the illness and treatment strategies;
- specialists should be well integrated with other parts of the care pathway. They play a key role in quality governance, training and the dissemination of best practice;
- use a comprehensive assessment of user-, product-, and usage-related factors to assess the needs of patients and caregivers with regards to containment products. This process should be standardised, valid and easily reproducible. The final decision regarding choice of product should remain with the end-user: the patient and/or the caregiver;
- the use of technology should be integral to the delivery of continence care. Technology should enable self-care and connect patients, caregivers and enable providers to monitor progress and troubleshoot problems;
- *for payers*: in order to provide the highest quality continence care, ensure care standards are incentivised. This can be achieved through stipulating the achievement of targets on clinical outcomes rather than operational measures alone, careful use of quality-related financial incentives, and an emphasis on clinical governance;
- establish accredited programmes of training for 1) nurses wanting to become continence nurse specialists, and 2) other health or social care professionals such as social workers wishing to improve their competence in delivering continence care.

2 Introduction

2.1 Introduction and context

2.1.1 Introduction

Incontinence, whether urinary or faecal, is an under-treated problem that imposes a considerable burden on the quality of life of patients and their caregivers. Whilst incontinence is often a treatable, manageable condition for which national and international treatment guidelines exist, the quality of continence care both at a global level and within national and local boundaries is variable.

There are currently no service specifications for continence services that have been designed for use internationally. This is a serious limitation as most healthcare systems are under severe financial pressure and may not be able to afford the time or resources necessary to design a care service specification, even though such an activity may improve patient outcomes and be, at the very least, cost-neutral. This expert panel has undertaken the ambitious task of detailing a service specification for global use that can be developed further to take into account specific local circumstances.

2.1.2 Scope

The objective of this document is to create a service specification that can be used by any organisation that commissions or pays for the provision of continence care.

Areas of continence care that are not covered include:

- paediatric care;
- care for people in long-term or continuing care settings e.g. residential/nursing homes;
- primary prevention.

2.2 Our approach

2.2.1 Methods

This service specification has been drawn up by an expert panel. Its composition is shown in Appendix 1. The approach comprised three phases:

1. Evidence gathering

The evidence gathering phase included:

- systematic literature search of databases;
- review and analysis of the grey literature on continence care and health policy;
- interviews with representatives from a variety of different backgrounds based in a selection of geographies. These interviewees included: payers, policy experts and influencers, academic researchers with an interest in incontinence, patients, patient group representatives, caregivers, physiotherapists, nurses, urologists, gynaecologists and primary care practitioners.

2. Synthesis of evidence

The synthesis phase involved:

- sharing and consideration of the collected evidence base amongst the expert panel;
- drafting of the service specification.

3. Validation

The validation phase involved:

- review of the draft by the expert panel;
- modification of the document by the expert panel - where there was disagreement regarding evidence for or against a particular service component, there was further discussion to reach consensus. If there was still disagreement, the issue went to a vote.

2.2.2 Underlying principles

The expert panel followed these principles when designing the approach to the service specification:

1. Optimum use of resources

- best use of available local resources;
- no duplication of provision;
- use of technology as an enabler;
- a conservative 'stepped' approach.

2. Equity of access and treatment

- optimising access;
- improvement in patient awareness and reduction of stigma.

3. Patient-centred care

- locally-delivered care;
- care provision by appropriately trained individuals;
- integrated service provision;
- use of multidisciplinary teams where feasible;
- avoiding unnecessary plurality of caregivers within the same discipline;
- promotion of self-management and patient education.

4. Upholding professional standards

- where possible, clinical care should be delivered according to evidence-based guidelines on continence care. Examples include the recommendations of the Fifth International Consultation on Incontinence (2013) [1] and the European Association of Urology guidelines on Urinary Incontinence (2013) [2]. Many other national or regional guidelines exist and should be referred to as appropriate for the region in consideration (see Appendix 2).
- healthcare professionals should be encouraged to use their clinical judgement avoiding undue influence of financial targets and incentives – when making decisions on clinical management and referral to other healthcare professionals.

5. Quality governance

- services should be led and assured by an interdisciplinary group consisting of specialist clinicians, continence nurse specialists, primary care physicians, physiotherapists, pharmacists, healthcare managers and patient representatives.

2.2.3 Modular specification

To achieve an internationally applicable continence care service specification, the approach that has been taken is, by necessity, modular, and sensitive to variables between different healthcare localities such as:

- population demographics and patient characteristics;
- cultural differences e.g. in healthcare seeking and disease recognition;
- levels of geographical healthcare access;
- levels of financial healthcare access;
- maturity and development of existing continence care provision;
- extent to which services are integrated;
- economic and regulatory levers available to influence healthcare provision;
- application of technology in the delivery of care.

Provision of the highest quality integrated continence services at reasonable cost may require fundamental changes in the healthcare economy that go beyond the scope of this document. These will involve a combination of changes to resource allocation, payment systems, regulatory codes and professional practices.

However we intend that an organisation commissioning or paying for a continence service in any part of the world can use this document to pick out those service components that they can reasonably expect to procure for their population. We have outlined some examples of how these service components could vary according to local needs and circumstances in section 4.

As part of our data-gathering, we have examined the current state of continence care in four countries (see section 5.1): the United States, the Netherlands, United Kingdom and India. We paid particular attention to the specific contexts of each country in line with the variables listed above, and demonstrated how our service specification could be applied in practice in each country. We hope that we have provided practical insight into how our specification could be applied across a diverse group of healthcare systems.

3 Impact of the problem

3.1 Definition and prevalence

3.1.1 Definitions

We have used the International Continence Society's definition of incontinence as 'involuntary loss of urine or faecal material' [1].

Urinary incontinence (UI) describes the involuntary leakage of urine and the majority of causes can be divided into three types: urgency incontinence, stress incontinence and mixed urinary incontinence:

- Urgency incontinence describes incontinence immediately preceded by the feeling of urgency.
- Stress incontinence describes incontinence on effort, exertion or on sneezing or coughing.
- Mixed urinary incontinence describes incontinence associate both with urgency and effort/exertion/sneezing or coughing.

Faecal incontinence (FI) is defined as the involuntary loss of flatus, liquid or solid stool that is a social or hygienic problem. Often the problem is multi-factorial. The term 'anal incontinence' is used, often interchangeably and the definition is the same [3]. For consistency, we have used FI here. Underlying types of faecal incontinence include:

- passive: the involuntary leakage or soiling of faeces without forewarning;
- flatus incontinence: the inability to control flatus;
- urge faecal incontinence: an inability to defer defaecation once the urge is perceived, for long enough to reach a toilet;
- soiling after defaecation may also occur, often related to impaired internal or external sphincter function.

3.1.2 Prevalence

UI in women

- The prevalence of UI in community-dwelling middle-aged and older women is estimated to be between 30 and 60% depending upon the underlying definition, the population and the setting in which the study occurred [4-8].
- In women over 70, daily UI ranges from 5% to 15% [3], Isolated stress incontinence accounts for approximately half of all incontinence, with most studies reporting 10-39% prevalence. With few exceptions, mixed incontinence is found to be next most common, with most studies reporting 7.5-25% prevalence. Isolated urgency incontinence is uncommon, with 1-7% prevalence, and where recorded at all, other causes of incontinence occur with approximately 0.5-1% prevalence.

UI in men

- A study in 2009 on UI in community-dwelling men showed pooled overall prevalence rates from 5% to 32%, with prevalence increasing with age [3].
- Owing to the differences in anatomy and the pathophysiology of UI in men versus women, there is a predominance of urgency UI in men (40-80%) then mixed forms of UI (10-30%), followed by stress UI (<10%).

FI

- FI (which includes involuntary leakage of flatus) occurs at all ages in both genders: 1-20% of adults are affected with FI, depending on the definition [3]. An estimated 0.5-1% of adults suffer an effect on their quality of life [9].

- Faecal and urinary incontinence commonly occur together, particularly in older, frail people with multiple impairments.

With the ageing of the world's population, particularly in industrialised countries, the overall prevalence of incontinence is increasing and often coexists with other chronic medical conditions [3].

3.1.3 Health-related implications

There are a number of associated conditions resulting from incontinence including:

- *Urinary Tract Infection (UTIs):* There is a direct association with acute UTIs with studies demonstrating both that UI can lead to UTIs and UTIs can result in UI [3]. Considering the prevalence of urinary incontinence, prevention and treatment of infection can have a significant impact on the use of antibiotic medications and therefore on the development of bacterial resistance. This is particularly the case in older people given the high prevalence of asymptomatic bacteriuria.
- *Depression:* Similarly, the emotional strain of suffering from UI can lead to depression while depression may exacerbate UI symptoms [3].
- *Falls:* There is an association between urinary incontinence, urinary urgency, nocturia and falls in older people. This association has been demonstrated in many epidemiological studies but underlying causes are unclear and likely to be multifactorial [10, 11]. There is evidence that there are gait changes in response to urgency and a possible association with impaired diverted attention [12]. Acute illness and frailty in older people may also play a role.
- *Pressure Ulcers:* Extended periods of exposure to moisture make UI and FI patients more prone to developing pressure ulcers [13, 14].

3.1.4 Impact on quality of life

Impact on the sufferer

Urinary and faecal incontinence have a major impact on a person's quality of life, For example, individuals with urinary or faecal incontinence may experience:

- embarrassment associated with leakage in social situations;
- reduced employment, leisure opportunities and social exclusion [15];
- significant strain on relationships especially between sufferers and their partners or informal caregivers.

The Canadian National Population Health Survey (1996/97) showed urinary incontinence to have a severe impact on quality of life, rated behind only Alzheimer's and stroke in people aged 12 and older, and highest in younger patients (<45) [16]. The level of quality of life impairment for an individual is an important factor in whether he/she seeks treatment for incontinence (see section 3.3).

Impact on the caregiver

Incontinence is often regarded as an intolerable burden for caregivers of disabled and/or elderly sufferers with multiple care needs [17]. It is one of the developments that may no longer make informal care at home a viable option, and indeed is one of the most common reasons for a person moving to long-term institutional care [3, 18]. There are a number of factors involved that lead to this difficult situation for the caregiver [19]:

- physical toll of replacing pads and/or taking the sufferer to the toilet regularly [20];
- psychological strain due to the full-time nature of the role [21] and restriction in leisure opportunities and social interactions [22];
- changes in the nature of relationship e.g. daughter/son or wife/husband to intimate nursing role [20];

- feelings of guilt causing the caregiver to 'hang on' to responsibility for caring past the point where they are truly managing the care load [17, 20].

3.2 Health economic impact

Incontinence presents a significant health and economic burden comparable with major global diseases such as arthritis and osteoporosis [23]. There are:

- direct costs related to health and social care – these may be taken on by the sufferer or the public payer/health insurer [24, 25];
- indirect costs e.g. cost of productivity of incontinence for sufferers and their caregivers [25];
- intangible costs i.e. the economic value of suffering of both sufferers and caregivers [25].

Most health economic studies on the subject exclude indirect and intangible costs because they are difficult to measure. However, although incontinence is rarely life-threatening, the chronic nature of the problem combined with the considerable effect on personal function and quality of life mean there is likely to be a large overall cost.

The health economic impact of incontinence is projected to increase substantially in the coming years with the increasing prevalence associated with ageing populations of developed countries around the world [25].

3.2.1 Direct and indirect costs

Direct costs

These costs may be paid either by the public payer/health insurer or directly out-of-pocket by the patient or a combination of the two. These include [25]:

- specialist and primary care visits;
- diagnostic services;
- treatments such as behavioural therapies, medication and surgical treatments;
- treatment of the consequences of incontinence e.g. UTIs, falls, sores (note that incontinence could be one of many factors leading to these 'consequences') [25];
- community care;
- residential aged care;
- cost of containment products and other assistive devices.

In 2008-2009, the estimated direct costs of incontinence care in Australia totalled (US) $1.1 billion [17] or approximately $51 per person and (US) $3.3 billion in Canada or $97 per person [21, 26-29]. In 2000, the total direct healthcare costs for urinary incontinence alone in the United States were estimated to be $13.7 billion for community-dwelling adults or approximately $51 per person [30, 31].

Indirect Costs

Indirect costs due to productivity loss from sufferers and caregivers and impact on quality of life are less easy to measure but are thought to be more significant than direct costs. An Australian study in 2011 estimated the productivity costs of incontinence for sufferers to be (US) $30.5 billion and opportunity costs for caregivers to be $2.4 billion [27, 32].The indirect costs of incontinence are more than 19 times greater than the direct healthcare costs in this example, and this is before intangible costs are taken into account.

3.2.2 Intangible Costs

The same Australian study mentioned above has estimated the intangible costs or 'burden of disease' to be equivalent to (US) $21.6 billion annually for community-dwelling individuals in Australia. To arrive at this figure, the disease burden was estimated in disability-associated life years (DALYs), and this number was then converted to a dollar value using an estimation of the Value of a Statistical Life (VSL) over the course of a particular year. The VSL is the monetary value placed by society on an individual anonymous life and has been derived by a number of health economic studies [32].

The total cost for Australia based on direct, indirect and intangible costs per year was estimated to be (US) $59.6 billion. The impact of incontinence is large and well worth further investment in providing additional or extended services.

3.3 Current problems in service delivery

There are a wide variety of continence care services across the world. These vary according to the inter-country variables mentioned in section 2.2.3.

Common issues that affect access to, and delivery of, continence care services include [33]:

- low levels of healthcare-seeking in people who are affected (see below);
- poor levels of access to appropriate medical care due to a combination of geographical isolation, lack of medical insurance coverage and inadequate care pathways;
- under-recognition by healthcare professionals (particularly physicians) of incontinence as a medical problem;
- treatment patterns that are often not adherent to clinical guidelines;
- inadequate provision of containment products not based on a comprehensive approach to sufferers' and caregivers' needs;
- low priority of continence care on the health policy agenda.

Low levels of healthcare seeking are a particular issue: fewer than half of people with incontinence spontaneously report the problem during a healthcare visit [34]. Several barriers need to be overcome for people to seek medical help, including:

- cultural taboos around incontinence [35];
- gender-specific issues e.g. females wary of having a full vaginal examination, or being seen by a male physician [36] [37];
- feelings of embarrassment [35, 38];
- mistaken beliefs regarding the nature of incontinence as a matter of self-control or a inevitable part of ageing [38, 39];
- feelings that the problem is not important enough to trouble the doctor [39];
- a lack of public knowledge around the natural history/causes of incontinence [40];
- a lack of awareness of locally available continence services;
- the recognition by medical staff of the effect of incontinence on the lives of sufferers and caregivers [41];
- the ability of medical staff to identify patients with risk factors for incontinence through active case-finding [9].

The parts of continence care that are most often not carried out well are the detection of cases and the initiation of relatively straightforward interventions not requiring a medical specialist. This poor level of basic continence care delivery could be traced back to the relatively little continence-related

content in higher education training across the major healthcare professional disciplines [42]: indeed, this includes basic nursing and midwifery curricula [43].

While some studies have concentrated on the cost-effectiveness of services in isolation [44], there is evidence in some cases to show that the routine trial of conservative treatments before surgical interventions is likely to be the most cost-effective treatment strategy [45-47].

It is important to recognise the downstream indirect and intangible costs of incontinence. Successful delivery of services at a community-based primary care level will likely pay for themselves through the beneficial impact on the independence of sufferers, and the improved well-being and productivity of both sufferers and their caregivers [32].

Additionally, there are low levels of integration of continence services resulting in duplication of provision, concentration of services in specialist centres and relatively low provision of community-based care [48]. Provision is often dictated by imbalances in the level of reimbursement versus the true cost of providing treatment. For example, in the United States, fee-for-service reimbursement has incentivised specialist provision of non-specialist activities across many common medical conditions [49]. In the case of continence care provision, this structural feature of the healthcare system results in a bias towards surgical intervention over more conservative treatment strategies [50, 51], as well as overuse of expensive and often unnecessary investigations such as urodynamic testing [52].

In low income countries, incontinence is not usually a priority, with only basic levels of practice performed by community care providers, if anyone at all. Many of the problems in continence care in these countries are related to the immaturity of the wider healthcare system. Addressing the continence care needs while these countries develop their healthcare infrastructure will likely require innovative solutions to make the most of limited resources. In section 5.1, we describe both the current service provision in continence care and how our service specification could be applied in a selection of higher-income countries and a single lower-income country.

4 Organisation of continence services

Health and service promotion is of vital importance given the barriers to providing effective treatment.

Therefore, in order for a continence service to reach and treat affected people successfully, a concurrent public health effort is needed to run with any improvement in service delivery. This requires the problem to be highlighted in the public domain and for its profile to be raised amongst policymakers and healthcare administrators.

4.1 No single optimum model for delivery

We do not believe that one single delivery model will be appropriate for all healthcare systems. The reasons for this are that:

- the varying demographic, economic, and cultural characteristics (set out in section 2.2.3) that will affect the nature of what can be defined as an optimum service;
- in many cases, the best-performing healthcare services are locally-derived and led by motivated individuals with specific interests and skills;
- to be cost-effective, any changes in service delivery will make best use of existing local structures;
- healthcare professionals have the ability to perform a variety of different roles in different domains if trained to do so.

4.2 Integrated continence care

We recommend an integrated approach to continence care. There are a number of definitions of integration:

- collaboration and communication between providers of the different stages of care e.g. primary and secondary care;
- a multi-disciplinary approach to address the care needs of a patient;
- working across social and healthcare boundaries in a coordinated fashion to help relieve the burden on the patient, the caregiver and the healthcare system;
- care centred around the patient, so that patients do not 'fall between the gaps' between various healthcare professionals, formal and informal caregivers.

Continence care warrants multi-professional interventions from health and social care as well as support by informal caregivers, in particular, for patients with multiple healthcare issues. Coordination, linkage and integration of different stakeholders is therefore key to ensure effective and efficient assessment and treatment, and to maximise autonomous coping strategies [53]. Working effectively in these ways can have significant benefits on the quality of services [48]. How to best achieve truly integrated care is a matter of considerable debate that has implications for whole health and social care systems and is out of the scope of this document. Nevertheless, some of the greatest challenges include:

- providing co-ordinated care for patients who have multiple healthcare issues and who are being looked after by a plurality of providers [54];
- overcoming funding and organisational boundaries between health and social care [54];
- distortions in the provision of healthcare due to adverse incentives e.g. 'fee for service' payment systems causing a bias towards surgical treatments where behavioural interventions could be just as effective (see section 3.3) [50, 51].

Communication between providers is important for patient-centred care: looking after frail elderly through an ‘integrated continuum of care’ from admission to hospital via the emergency department and subsequent discharge back to the person’s own home can halt a decline in independence [55]. In section 0, we explain how to provide patient-centred care for incontinence through a case co-ordinator model.

Misaligned financial incentives can cause unwanted distortions in the healthcare system. Pure fee-for-service payment structures, for example, can lead to the over-utilisation of healthcare resources resulting in unnecessary investigations and inappropriate (and often invasive) treatments [49]. If payers are able to align incentives for providers of health and social care to work together to achieve tangible patient-related outcomes (see section 7) whilst appropriately reimbursing the cost of delivering care [56], this may help prevent some of the imbalances detected currently in the provision of continence care [57, 58].

4.3 Components of a continence care service

Taking into account the varying ability of different countries to provide a comprehensive service, we have broken down the elements of the comprehensive service into modular components allowing payers and commissioners to select component parts which may be more practical or feasible to adopt within their particular healthcare environment:

- case detection;
- initial assessment and treatment;
- case co-ordination;
- caregiver support;
- community-based support;
- specialist assessment and treatment;
- use of containment products;
- use of technology.

Figure 1: Components of a continence care service

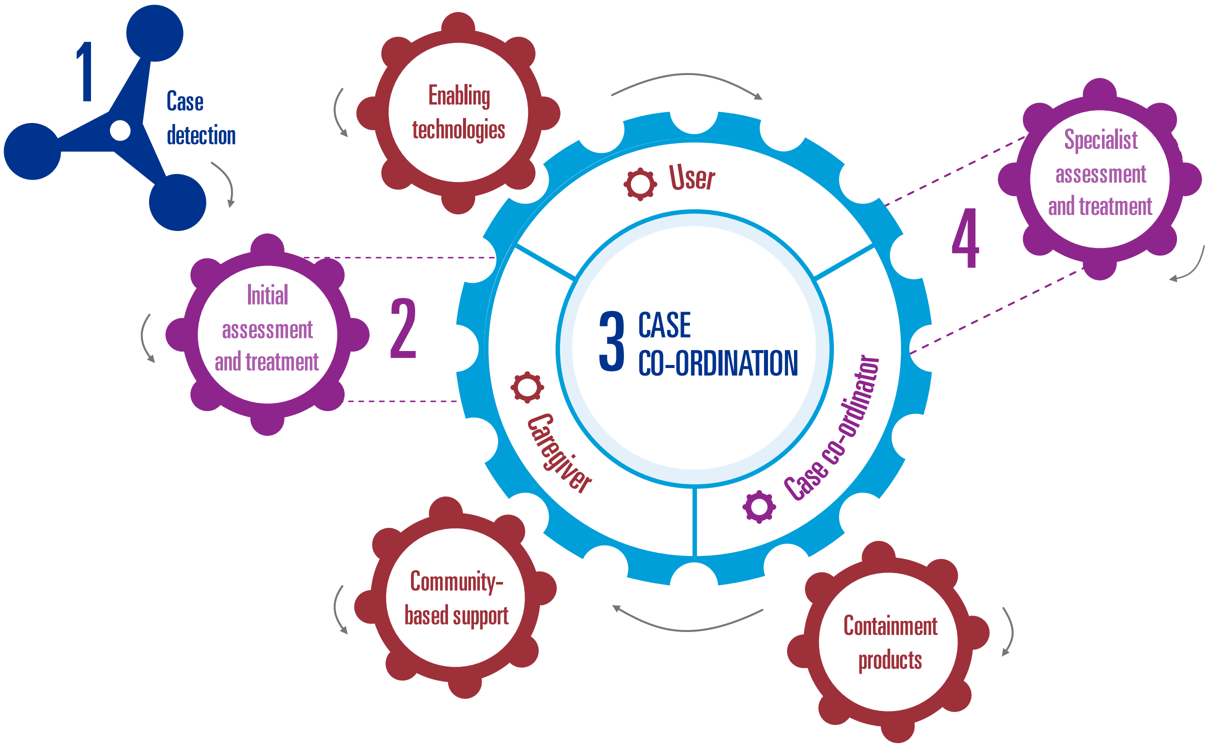

Figure 1 is a simplified representation of the different components of a comprehensive continence care service. Each of these parts may be delivered or organised by one or more trained healthcare or (in the case of community-based support) social care professionals. In each case we will express a preference for the healthcare training/background that would be most suitable according to the evidence-base, but also provide examples of alternative options in order to be mindful of local circumstances and resource constraints.

In developing the concepts behind each service component we have assumed that healthcare professionals will adhere to the international evidence-based guidelines contained in the Fifth International Consultation on Incontinence (2013) [1] and others mentioned in section 2.2.2.

4.3.1 Case detection

Case detection refers to the first point of contact at which a sufferer reveals their incontinence-related problems to a healthcare professional. Cases of incontinence may become known to a healthcare professional in a variety of circumstances. For example, incontinence may be detected as a result of:

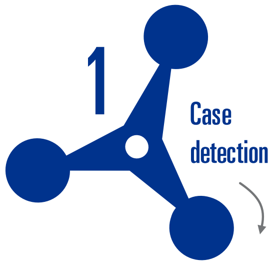

- patient-initiated clinical consultations with a healthcare professional for the specific purpose of addressing incontinence symptoms;
- being mentioned 'in passing' by a patient, relative or caregiver as a problem during a consultation with a healthcare professional;
- general history-taking by a healthcare professional e.g. a gynaecologist asking specifically about incontinence when assessing a patient for a different gynaecological issue;
- general health checks;
- incidental discovery by community nursing or home care staff;
- incidental discovery during a hospital admission;

- a systematic screening programme for incontinence.

The healthcare provider or professional who detects incontinence could be:

- a patient's main primary care contact e.g. primary care professional (e.g. physician, nurse practitioner), community nurse, community health worker (if limited access to primary care);
- an open-access continence service;
- a specialist healthcare professional with an interest in continence e.g. care of the elderly physician, urologist, continence nurse practitioner, gynaecologist, colorectal surgeon;
- a community nursing, home care, outreach services or other social care provider;
- a community pharmacist;
- any other healthcare professional e.g. a physician or nurse working in an alternative setting.

In many current services, the route to continence care detection may affect the quality of subsequent continence care received because different healthcare professionals vary in their:

- confidence and ability to undertake the *initial assessment and treatment* of incontinence [59, 60];
- access to nurses trained to deliver continence care;
- access to specialist doctors such as urologists, gynaecologists and colorectal surgeons, and specialist physiotherapists;
- awareness of dedicated continence services;
- awareness of best practice in the use of continence aids and products.

Each route to detection represents a possible entry point into care. Depending on skills and experience, those healthcare professionals who detect cases then either perform the assessment themselves, or refer on for *initial assessment and treatment*.

Robust referral pathways linking all possible routes of case detection to appropriate assessment and treatment are important to ensure patients receive timely and effective care. A poor initial experience of healthcare-seeking may discourage patients, while reinforcing personal and cultural beliefs that denigrate the status of incontinence as a genuine medical problem. Appropriate referral will depend on adherence to structured referral guidelines/instruments to ensure that the appropriate information is captured, and also to prompt the referring clinician to try various approaches first or order tests to support the referral.

Given the satisfaction of sufferers with the care provided by continence nurses (see section 4.3.2) and the gender-specific issues with seeking care (see section 3.3), it may well be more effective for nurse-led continence services to be open access, allowing patients to bypass the main primary care contact, if preferred.

In healthcare systems where there is a capability to take a population-level overview, such as with the insurer, primary care population or area health authority, there may be the opportunity to look at discrepancies between observed and expected rates of incontinence. A well-developed predictive analytics function can provide the potential to detect more elusive cases, and to intervene early in these patients. By identifying those patients who are most at risk of developing incontinence, this may provide the basis for redesigning the service to target these groups.

KEY RECOMMENDATION
Ensure ease of access by the establishment of robust referral pathways from detection of incontinence through to appropriate assessment and treatment.

4.3.2 Initial assessment and treatment

Initial assessment and treatment refers to an initial visit, clinical assessment, and treatment for a patient with incontinence [1]. This component demands particular attention as its conduct is often non-adherent to established clinical guidelines and could provide much health and economic benefit relative to cost. *Initial assessment and treatment includes:*

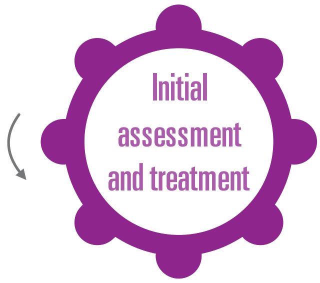

- clarification of patient goals and expectations;
- clinical history and examination;
- simple tests as indicated by established guidelines;
- initial interventions;
- referral to specialists;
- advice on and prescription of containment products.

Initial assessment and treatment is optimally enacted by a dedicated local nurse-led continence service, situated in the community or (in some cases) adjacent to a specialist clinic – and the case detector should be able to refer to a dedicated nurse-led continence service without being responsible for the *initial assessment and treatment* of a patient with incontinence.

We recommend this model because:

- nurses with the requisite training are capable of managing and treating incontinence more effectively than primary care physicians [44, 61-63];
- nurses are able to triage and manage independently a significant proportion of the patients who are routinely referred to specialist care [64, 65];
- many primary care physicians are unaware of clinical guidelines in the management of incontinence [59], or find them difficult to adhere to for a variety of reasons [60];
- there is evidence that patients appreciate the good communication skills and holistic care provided by nurses [66, 67], while patient satisfaction is higher with primary care nurse-led services in general when substituting for primary care physicians, with no differences in outcomes [68].

While there is no conclusive evidence that the direct costs of providing a nurse-led service are more or less (nurses may be lower cost but physicians may be more productive in terms of time spent per patient [44, 62]), the marginal extra cost of providing a nurse-led service compared with a physician-led service could be justified when one considers the relatively large indirect and intangible costs of incontinence if sufferers are left under-treated (see section 3.2).

The majority of evidence for the effectiveness of nurse specialists in continence care originates from the United Kingdom, the United States, the Netherlands, Canada, Australia and Sweden (urotherapist model). We can therefore recommend this model in countries where nurses have the funding, support and training programmes required to substitute in for physicians.

Where there is a paucity of continence nurse specialists, it will be necessary to train existing professionals, especially those who currently see people with incontinence, and those who are well placed to deliver continence care. Healthcare professionals in general are capable of learning a variety of skills, often outside of their usual domain. For example, in the Netherlands, primary care-based general practitioners' assistants (called Praktijk Ondersteuners Huisartsenzorg, or POHs) have taken on an increasingly significant role in the management of UI in some regions [69]. In rural Bangladesh, research is currently being undertaken to investigate the role of village 'paramedics' or other trained healthcare workers to carry out basic continence care (data not yet published).

Where there are no professionals effectively managing incontinence, we recommend a focus on training and educating primary care physicians, primary care-based nurse practitioners, village

‘paramedics’ or other primary care/community-based healthcare professionals to enhance competency to perform the role [59, 60, 70, 71].

KEY RECOMMENDATION
Shift the responsibility of basic continence care away from primary care physicians to continence nurse specialists in primary care, where available. Where continence nurse specialists are unavailable, train existing healthcare professionals such as primary care-based nurse practitioners, community nurses, physician’s assistants, or, in developing countries, local community healthcare workers, to provide evidence-based continence care.

4.3.3 Case co-ordination

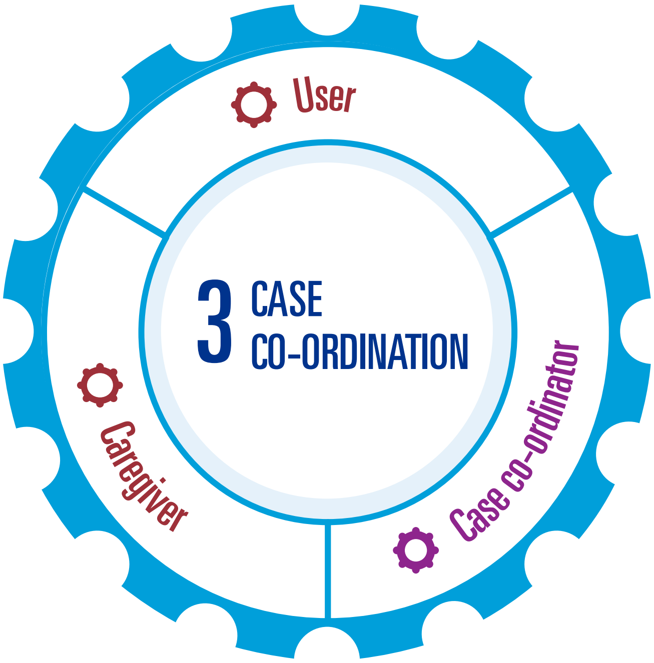

Case co-ordination describes the supervision of all aspects of care of a patient. *Case co-ordination* is important for providing 'patient-centred' care and ensuring patients do not 'fall through the gaps' between providers [72]. A single point of contact for patients who may be unsure about who is responsible for their care can ensure the smooth and timely delivery of:

- initial management and investigations;
- specialist care;
- information and advice for patients and caregivers on managing the condition;
- information and advice for patients and caregivers on usability of containment products;
- related social care interventions e.g. increased frequency of formal caregiver visits (where available).

The role of case co-ordinator

We recommend the deployment of a case-co-ordinator, where available, to provide a single point of contact for patients who can co-ordinate the multiple agencies involved in providing continence care. Integrated care models have demonstrated positive outcomes in other chronic diseases. For example, in patients with diabetes and depression, a patient-centred collaborative care model run by experienced nurses gave better diabetic control and improvement in depression symptoms [73]. A simple disease management programme for chronic obstructive pulmonary disease (COPD) that involved the use of a COPD case manager significantly reduced emergency department visits and hospital admissions [74].

The absence of a central individual who is purely focused on continence care may result in un-coordinated care with potential for either a lack of communication between different providers, duplication of provision or the responsibility of co-ordinating care remaining with the primary care contact, who may not place a priority on continence care.

Healthcare professionals responsible for *initial assessment and management* will usually be best placed to perform the role of *case co-ordination* in patients with incontinence. Nurses with specific training in incontinence are particularly suitable for this role: patients with lower urinary tract symptoms have been shown to express greater satisfaction with nurse-led telephone follow-up consultations to clinic visits, with advantages including more consistent follow-up with the same healthcare professional, greater convenience and cost savings [75].

When the patient is suffering from a complex primary illness, it is important to avoid duplication of care [76]. For example, in the case of a neurological patient with multiple sclerosis, continence care may be one of a number of clinical problems. Similarly, elderly patients will often have a complex array of conditions with associated health and social care needs that require a wider lens than that provided by a continence case co-ordinator, i.e. they may be accompanied by a generic case or care manager. In these circumstances, the continence case co-ordinator will have to adapt to take a more facilitative and advisory role, filling in the gaps in continence care where they exist. The role would then be one oriented more around:

- providing input on appropriate health and social care interventions to other specialist nurses e.g. multiple sclerosis or diabetes nurses or generic case or care managers who already have a more holistic view of a patient's specific needs;
- acting as a gate-keeper/triage service to each of the different components of continence care to provide assurance on appropriateness of referrals.

In order to incentivise integrated continence care for the population covered by a payer, we recommend (see section 6.2) tying payments to a patient experience outcome indicator relating to patient-centred or integrated care, together with outcome indicators relating to clinical and quality of life improvements for patients *and* caregivers [57, 77, 78] (see section 7).

KEY RECOMMENDATION
Where possible, use a case co-ordinator to ensure collaborative working, especially to help delay or prevent admission of patients to permanent care settings; given the general trend to more integrated clinical pathways, in particular concerning patients with multiple morbidities, it is necessary to strike a balance between specialisation and holistic case management approaches.

*Please see section 8 for example profiles of nurses with specific training in incontinence.

Enabling self-care

Modern medicine increasingly values patient autonomy in decision-making in preference to the traditional model of paternalistic medicine [79]. This has been enhanced by societal trends such as consumerism and the increased availability of health-related information via the internet [80, 81].

Patients suffering from incontinence likely wish to be at least well-informed [82] and may prefer to have a more active role in treatment decision-making [83] compared with other patients. This presents an opportunity, particularly where current services lack the resources and manpower to deliver conservative treatment strategies.

Regardless of the healthcare system, we recommend taking measures to help empower the patient including:

- active promotion and sharing of self-management tools and techniques [84, 85];
- provision of information and advice on containment products (see section 4.4.6);
- promotion of innovative uses of technology (see section 4.3.7);
- placing an emphasis on shared decision-making between clinician and patient (and caregiver where appropriate);
- use of educational campaigns particularly around symptoms, likely investigations and treatment choices.

KEY RECOMMENDATION
Promote use of self-management tools and techniques; provision of information on the use of containment products; use of enabling technologies; an emphasis on shared decision-making between healthcare provider and patient/caregiver; and educational campaigns on the nature of the illness and treatment strategies.

In rural areas, specialists should also undertake 'outreach' programmes to cover sufferers who are unable to travel far because of economic constraints, personal full-time commitments, and due to fear of suffering an episode of leakage.

Efficacy of self-care is strongly dependent on patient motivation – particularly in the case of lifestyle and behavioural interventions. This underlines the importance of using tailored treatment strategies that are aligned to individual goals and expectations [86].

Caregiver support

There are a number of measures that a case co-ordinator can take to reduce the burden of care for caregivers of patients with incontinence including [20]:

- distribution of information on best practice in caring for incontinence;
- advice on and access to appropriate containment products;
- education and training programmes for caregivers;
- provision of flexible respite services at the local level.

Depending on the nature of the patient's coverage under their healthcare plan, some, none or all of these measures may be applicable to achieve the best value care for the patient and caregiver. If the plan has no coverage of social care aspects, the first two are still applicable to any effective continence service. The latter may be applicable where the payer is also responsible for social care e.g. a government-funded healthcare system.

4.3.4 Community-based support

Taking into account the overall impact of incontinence described in section 3, it is vital that providers of *community-based support* can step in to fill the gaps in ability to self-care and where patient and/or informal caregiver is restricted. This will involve helping with tasks such as:

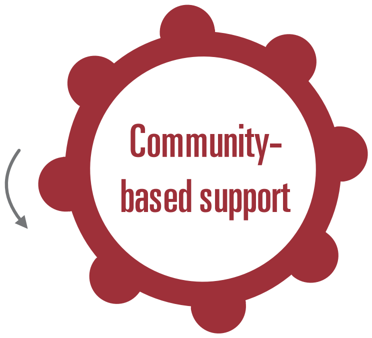

- helping the patient to the toilet;
- changing clothes, pads, linen;
- helping with laundry for soiled clothes, linen;
- communicating with healthcare professionals when the patient's condition deteriorates.

The provision of formal caregivers is an important element of sustaining disabled or frail patients' quality of life and will help patients avoid or delay admissions to long-term care.

Professional social care may be provided by public, private for-profit and non-profit organisations. Coordination, joint care-planning and communication between the case-coordinator, payers, providers, users and informal caregivers will help to avoid duplications, care gaps and avoidable hospital admissions.

4.3.5 Specialist assessment and treatment

The *specialist assessment and treatment* component refers to the treatment of incontinence by medical specialists such as urologists, gynaecologists, colorectal surgeons and care of the elderly physicians, or other healthcare professionals with specialist training such as physiotherapists.

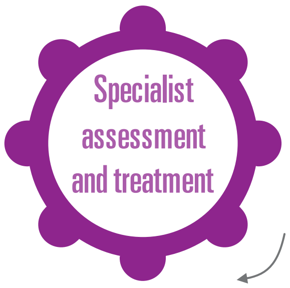

Specialist assessment and treatment includes the following elements:

- specialist clinical assessment;
- specialist investigations;
- specialist treatments.

Specialists in continence care should be focused on: those with severe symptoms or who are unresponsive to more conservative treatment strategies; complex patients; and those with a clear indication for more invasive treatments as per the clinical guidelines [1].

Care for incontinence associated with, or secondary to, other illnesses should still be organised by these elements. There will be variations according to the specific tests and treatments required. For further detail see section 0 on how these elements will apply to different profiles of patients.

While providers of *specialist assessment and treatment* are recommended to be separate from those providing *initial assessment and treatment* it is important that there is integration with other components of the service through the case co-ordinator. Specialists have an important role in facilitating the sharing of best practice and training as well as in quality governance. The integration of an academic centre with a community-based service has proven to be challenging in the past with barriers to overcome regarding funding sources and existing healthcare infrastructure. Nevertheless, this model should be feasible [87].

> ***KEY RECOMMENDATION***
> *Specialists should be well integrated with other parts of the care pathway. They play a key role in quality governance, training and the dissemination of best practice.*

4.3.6 Use of containment products

Containment products are an important part of the management of incontinence. Containment products should be provided either during the treatment process or when cure is not possible, through a strategy of 'contained incontinence'.

In many countries, patients are provided with containment products free of charge but they are often left wanting by provision that is neither sensitive to clinical need [88], nor wider care needs of patients. The international standard for the evaluation of containment products (ISO 15621:2011) recommends consideration of a number of factors separated into three categories:

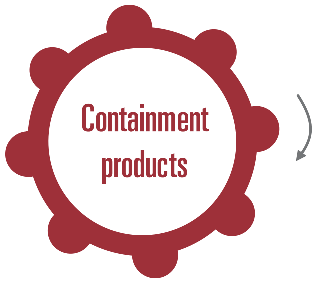

- user-related factors: quality of life, independence or assistance, nature of incontinence, end-user characteristics, activities, individual needs, handling products;
- product-related factors: freedom from leakage, freedom from odour leakage, skin health, comfort and fit, discretion;
- usage-related factors: ergonomics, needs of caregivers, information supplied, disposal facilities, laundry facilities, sustainability and environment, product safety, cost.

We recommend a comprehensive approach taking the above factors into account when prescribing or recommending containment products. We place particular importance on the following criteria:

- nature and severity of incontinence;
- functional and cognitive capabilities of the patient;
- care situation.

Where the continence care service also funds the provision of products, a standardised assessment tool should be used to reduce variation in provision. The needs of each patient can then be reassessed periodically to ensure appropriate consumption of products, avoiding the need for rationing [78, 89].

In order for patients and formal and informal caregivers to make informed decisions regarding containment products, patients should be provided with information on the ranges of products available and factors (see above) to consider when making the choice [90]. This should be supplemented with samples to test the best possible product to suit patient and caregiver needs.

KEY RECOMMENDATION
Use a comprehensive assessment of user, product, and usage-related factors to assess the needs of patients and caregivers with regards to containment products. This process should be standardised, valid and easily reproducible. The final decision regarding choice of product should remain with the end-user: the patient and/or the caregiver.

4.3.7 Use of enabling technologies

Technology can be an increasingly useful enhancement to current modes of continence care in the future. While some population groups are still disadvantaged concerning access and skills to use ICT applications, further developments will depend on patients' acceptance, cognitive abilities and affordability. The following areas will therefore have to be developed in close cooperation with payers, patients and professionals.

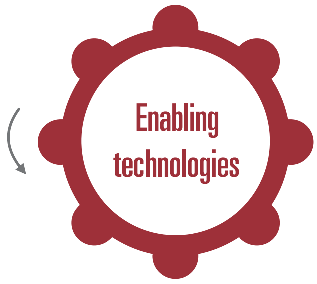

The use of telehealth services may connect healthcare professionals to patients with limited physical access e.g. those who live in rural areas. Potential uses include:

- video consultations;
- remote monitoring, where patients are able to send data regarding their bladder outcomes (e.g. bladder diary) to a central station for assessment by the healthcare worker for discussion, feedback and further treatment planning. One example may be the use of a sensor inserted into a containment product: the sensor can monitor incontinent events and transmit the information wirelessly.

Telehealth services could fill the gaps where resources and manpower are lacking. The use of mobile SMS text messaging for prevention, surveillance, self-management and compliance in developing countries is well established [91] and there is no obvious reason why such a medium could not work well in developed healthcare systems.

Barriers to healthcare seeking such as embarrassment, not wanting to 'bother the doctor' and cultural stigma are less of an in issue online and this setting may be a viable alternative to face-to-face care in many cases. There are a number of other ways the internet can be used, including:

- internet-based treatments that include e-mail support and cognitive behavioural exercises [92]. Mobile applications that aim to deliver the same treatments are currently under investigation [93];
- self-assessment tools [94];

- facilitated patient networks [95];
- facilitated professional networks [96].

These tools also provide a role in continence promotion as patients and healthcare professionals communicate about their experiences of receiving and providing care respectively. An example of a professional network from outside continence care is the Netherlands' *ParkinsonNet*: an ICT-enabled community-based network of physiotherapists that is able to provide higher quality, lower cost care compared to usual care [96]. Well-developed patient networks may be a valuable source of data on treatment effectiveness and side effects.

Lastly, advances in the sophistication of electronic medical records could potentially enhance case detection and evidence-based management and treatment [97]. Enhancement of case-detection could take the form of automatic notifications of patients at high risk or through the active analysis of health record data to help target services at high-risk groups.

KEY RECOMMENDATION
The use of technology should be integral to the delivery of continence care. Technology should enable self-care and connect patients, caregivers and enable providers to monitor progress and troubleshoot problems.

Figure 2: Summary – continence care service specification

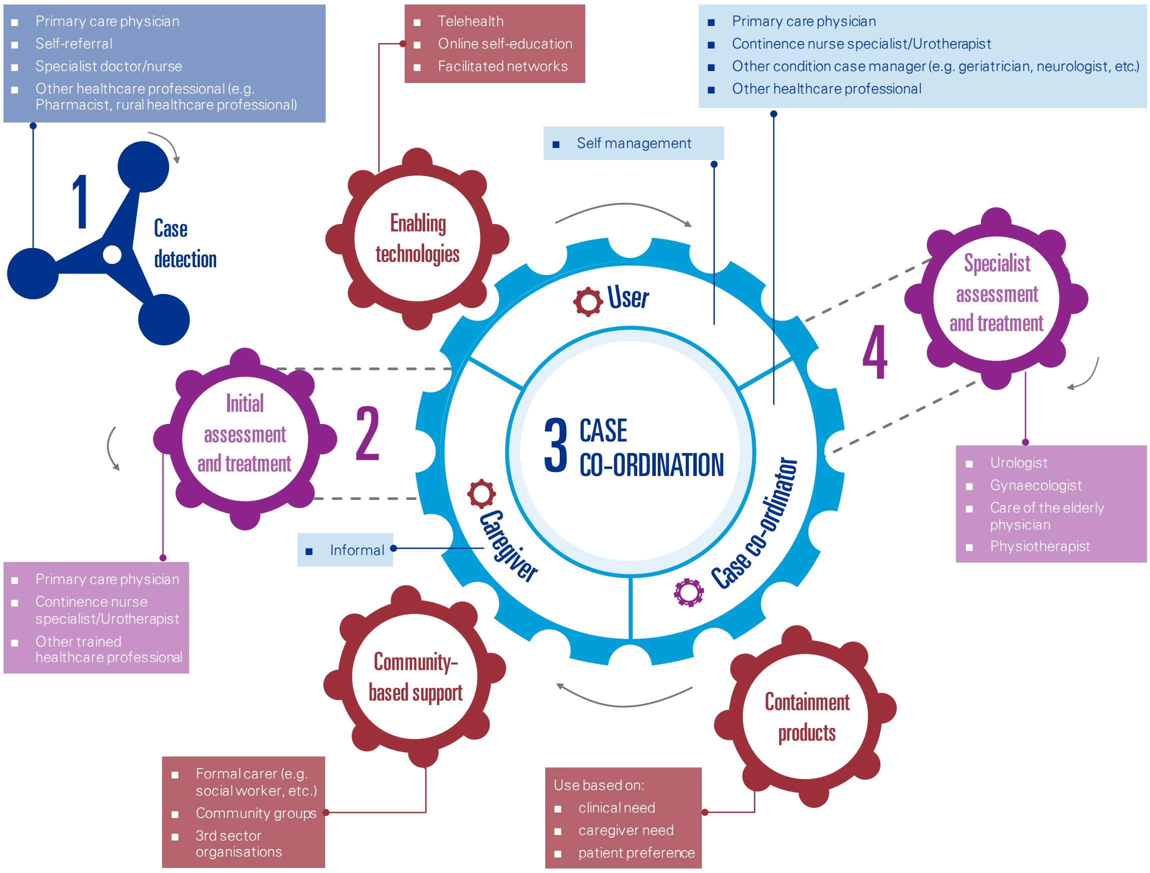

5 Real world applications

5.1 Local applications of a continence care service

As part of our evidence gathering, we evaluated current continence care delivery in four countries: The United States, the Netherlands, United Kingdom and India. Configurations of services are decided by a number of factors including (but not exclusive to):

- payment incentives;
- availability of primary care physicians/continence nurse specialists/specialist doctors;
- state and private healthcare funding coverage;
- access to social care.

Below we outline the current and optimum configurations of continence care in each healthcare system, based on information gathered through our interviews with representatives from those countries, and the recommendations we have made.

5.1.1 Current and optimum configurations of continence care services

United States (current)

Continence care in the United States is extremely variable – reflecting the range of healthcare eco-systems across different regions, states and cities. However, in most cases the healthcare system is driven by fee-for-service reimbursement even in a not-for-profit setting. Financial incentives drive the provision of services. ‘Profitable’ services - with favourable pricing versus the cost of provision – encourage a greater range of choice and competition over less remunerative services. With a few exceptions, incentives are not aligned to the long-term or overall health status of the patient. In the area of continence care, this manifests itself in a preponderance of specialists providing continence care: from more conservative treatment strategies to more invasive interventions such as surgery resulting in a greater overall cost to the healthcare economy.

Coverage of continence care depends on the plans taken up by individual patients. Treatment may be covered by insurance or public payers such as Medicare/Medicaid but containment products are generally purchased out-of pocket. Containment products are rarely recommended by physicians or other healthcare providers, as individuals will use their own judgement to select individual products and often do not present to healthcare providers.

Primary care physicians and advanced practice providers (APPs) including nurse practitioners and physician assistants, typically provide basic first-line conservative treatment (e.g. dietary modifications). These APPs are available in the primary care setting to meet the growing shortfall in the number of primary care physicians. Usually, comprehensive conservative and medical treatments are provided by APPs who have been informally trained as advanced practice continence nurses or pelvic floor nurse specialists working in the outpatient setting, which may be a specialty practice (e.g. urology, geriatrics, women’s health services). Some may have continence certification from a nursing organization (e.g. WOCN, SUNA).

There is also increasing use of capitated payment systems that set a fixed reimbursement rate to cover a period of care for an individual. Despite this, prevention remains an immature concept as the public and clinicians are not focused on preventing incontinence and for many it may not even be viewed as an issue. The majority of patients ‘self care’ i.e. they live with incontinence without seeking healthcare. The continence care pathway is usually not well integrated and the pathway is neither clearly defined nor standardised. Patients will generally be either referred by their primary care physician or self-refer to access specialist services, and many are not multi-disciplinary.

United States (optimum)

Significant improvement in the state of continence care in the United States would require fundamental changes in the healthcare economy to encourage investment in continence services in such a way that reduces risk and costs of providing continence care. This would necessarily involve greater integration of health and social care, more multi-disciplinary working and clearly defined pathways between primary, secondary and tertiary care providers. With the increasing prominence of the *accountable care organisations (ACOs)* advanced under the recent healthcare reforms, the structure of the healthcare system(s) could be more conducive to making the required improvements.

A concerted public health campaign is required to raise the profile of continence care in the United States. There are a number of patient advocacy groups such as the National Association for Continence, the Simon Foundation for Continence and the International Foundation for Functional Gastrointestinal Disorders seeking to improve patient awareness of services and provide education and advice. Working with such groups will help providers raise awareness of incontinence as a medical problem and encourage healthcare-seeking behaviour. Equally, continence promotion can help raise awareness in primary care physicians who are perfectly placed to take on the role of *case detection.* However, considering the poor track record of primary care to pick up cases of incontinence, open-access services led by nurses with specific training in incontinence (see below) may improve the numbers successfully seeking help for incontinence.

Considering the widespread lack of adherence to evidence-based clinical guidelines in the continence care delivered by primary care physicians, the optimum configuration of services will typically involve the use of APPs who have part of the primary care responsibilities, to provide *initial assessment and treatment* and *case co-ordination*. The ideal would be for these graduate-level practitioners to acquire further skills in continence care. However, there are few dedicated continence care education programmes (basic or graduate-level) to train nurses to the required level. Specialist doctors and physiotherapists should only be involved once conservative treatments have failed or when there is a clear indication for *specialist assessment and treatment*. As there are educational opportunities for APPs or registered nurses to learn the skills of providing a nurse-led continence service, it will be important to focus efforts on training generalist healthcare professionals (RNs, APPs) who will take on responsibility for continence care.

The role of community-based support is important but also challenging to implement. Community health workers, who are currently based mainly in rural areas, may be part of the solution. Because of the lack of available healthcare infrastructure these non-medically trained individuals are beginning to be used in diabetes care. Unfortunately, community healthcare workers are not reimbursable for their role in the care of anyone other than those on public health programmes. There do exist rural health clinics and native American health services that would be potential places to employ community health workers without necessarily requiring healthcare payer funding. These clinics, which are funded by the Federal Government, would employ these individuals and have the freedom to choose how to use them.

The increasing role of Accountable Care Organisations in the U.S. healthcare system should help aid a trend to more integrated care and greater emphasis on more conservative treatments. The Accountable Care Organisation would need to contract with providers in such a way that routes patients with symptoms to a low-cost model that can manage the majority of patients and ensure effective onward referral of more difficult cases. Nurse-led continence services would be well equipped to meet these requirements by:

- co-ordinating care with an informal caregiver, and any other relevant healthcare professional regarding their holistic care needs;
- acting in a facilitatory/advisory role when working with neurologic/care of the elderly/other nurse specialists who have a better understanding of the holistic care needs of the patient;

- providing evidence-based continence care based on a conservative step-wise approach unless there is clear indication for specialist referral;
- making best use of technology to enable self-care and to share information amongst healthcare professionals;
- achieving certain outcome and performance targets (see section 7).

Accountable Care Organisations may also find themselves in the useful position of being able to specify requirements for providers to share their datasets and insights from their analyses. This can potentially be used as a platform to perform predictive analytics - to pick out those patients who are most at risk of developing incontinence, and therefore look at ways to redesign the service to detect more elusive cases, and to intervene early in these patients.

In terms of measuring the performance of insurers and providers the Centers for Medicare and Medicaid Services (CMS) has been rating the performance of plans according to 5 priority areas called 'Stars' – one of these Stars is 'improvement in bladder control'. Using these ratings, plans can have their premiums adjusted according to their performance. This is a potentially useful lever to improve the performance of insurers and, through them, providers of continence services or elderly / gynaecological / urological care.

Netherlands (current)

The Netherlands' 'Bismarckian' model of healthcare is driven by market forces and consumer needs but is significantly more constrained by regulation compared with the United States. Each citizen is required to purchase a minimum package of insurance from the insurer of his/her choice. The minimum package is decided by the Ministry of Health and the coverage of continence care is a mandatory requirement.

The Ministry of Health sets overall health policy but hospital boards may choose to prioritise certain services over others, while physicians are responsible for decision-making at individual patient level. Insurers however have the countervailing power to buy best quality care for their patients. Because of that role, insurers become more involved in what type or amount of care is provided.

Primary care physicians, home care organisation staff and pharmacists are often the detectors of cases of incontinence. *Initial assessment and treatment* is also carried out by primary care physicians while, increasingly, this role is taken on by the *Praktijk Ondersteuner Huisartsenzorg* (or *POH*) substituting for the primary care physician. *Initial assessment and treatment* is not delivered well in Holland. Primary care physicians commonly do not adhere to guidelines: the default intervention is often the prescription of containment products with no trial of evidence-based treatments. Only a small proportion of those requiring specialist medical or physiotherapy assessments are referred on for appropriate escalation of treatment. In some areas, continence nurse specialists may offer *initial assessment and treatment* in the hospital setting. These nurse specialists may take on the role of case co-ordinator.

There are currently three levels of continence nurse specialisation in the Netherlands. Registered nurses with a secondary vocational education (MBO) receive 17 days' specialist continence training over a 12 month period at a course run by the St. Antonius Academy Nieuwegein. Another 17 day continence specialisation course is administered by the Hogeschool van Arnhem en Nijmegen for registered nurses with a higher professional education (HBO). Approximately 500 HBO-level continence nurses have been trained, of which 350 are currently working in the field of continence care, mainly in hospitals with only a small proportion in home care organisations. While some work with pharmacists, they generally do not work with family physicians. This level of continence nurse is not allowed to carry out a physical examination or to prescribe containment products or medicines, although some initiatives have been taken to make it possible for them to prescribe. A small number of continence care nurse practitioners with a Masters degree (5 nurses in the whole of the Netherlands at the time of developing the service specification) have been trained to carry out physical examinations and to prescribe containment products and, in the future, medicines also. They

are educated in somatic care and can follow additional training in the hospital where they work. They cannot be paid directly by an insurance company but would usually be working alongside a urologist or gynaecologist.

Boundaries are often observed between different types of services, because each type of care has its own source of funding. For example, primary care and hospital care are part of the Health Insurance Act; nursing care is part of the Exceptional Medical Expenses Act; and home (social) care is part of the Social Support Act. This causes conflicting incentives and may cause adverse consequences such as:

- Duplication of provision: medical specialists receive financial compensation per specialty (gynaecologists, urologists). Continence care is part of a fee-for-service model, stimulating duplication of provision of care.

  Some hospitals have been able to organise special agreements with insurers which include a price for multi-disciplinary care. This can encourage efficient and patient-oriented care in these hospitals;
- Lack of co-operation between primary care and hospital care: there is no incentive to treat patients in the most cost-effective setting. From the hospital's perspective, referral back to primary care results in less income;
- Lack of coordination with health and social care providers e.g. home care agencies;
- Over-utilisation: many patients receive surgery where effective conservative therapies could have been used as an alternative. (Note: physiotherapy in the hospital setting is not included in the minimum care package, whereas surgery and medicines are). This financial incentive stimulates use of hospital care instead of primary care use. There is also overuse of expensive investigations such as urodynamic testing.

In some regions, hospitals have built a network of connected first-line professionals who have access to guidelines and use shared IT facilities. However, this is not standard. Primary care physicians do not always know which continence care providers are available in their region, while the care provided by hospital and at home do not always connect well. For example, when a sufferer has visited the hospital for the first time, the hospital nurse looking after him/her has a responsibility for the continuity of care, which can include prescribing the right containment products or calling in the 'transfer office' which can arrange care in the home setting - this coordinating role is not always carried out.

Continence care included in the minimum package of care includes all hospital services, physiotherapy and containment products. The insurer pays for these services and materials. Depending on the type of insurance, patients pay co-payments/out-of-pocket costs or deductibles. A number of insurers have a purchasing strategy aimed at controlling the costs of containment products. Elements of this strategy include:

- limiting the number of potential suppliers/distributors with whom they contract, thereby increasing the volume purchased per supplier and their bargaining power;
- reimbursement based on a fixed fee per day instead of a fee per package of incontinence material provided. This leads to an incentive to limit the amount of material used per day, and to avoid the use of unnecessary material. The insurers have defined different categories of patients, with differentiated daily fees.

The provision of containment products has recently changed in the Netherlands. Previously there was more freedom of choice as the GP wrote a prescription for containment products and the patients were then provided samples by the pharmacy or medical device retailer with samples from which to select the type of product that best suited their needs. Now that the insurers want more control they have installed a system with categories of patients where mainly the severity of the leakage decides the daily allowance which leads to more restricted provisions which may not meet the real needs of patients.

As in the United States, more payers are using capitation-based payment systems which may be a useful way to incentivise more cost-effective preventative care. However, insurance contracts with insured individuals currently last a maximum of one year, presenting a barrier to such long-term thinking on the part of insurance companies.

Netherlands (optimum)

We recommend that the focus of continence care be shifted to the primary care setting, where we propose that increased numbers of continence care nurse practitioners be located. Because there are currently so few of these nurse practitioners we suggest that existing HBO-level continence nurses receive further training to allow them to practise in the primary care setting and to prescribe containment products and medicines. These nurses would take on the role of providing *initial assessment and treatment*, while other primary care professionals, community pharmacists and home care agency staff would primarily perform the *case detection* role.

The primary-care based continence care nurse practitioners would collaborate with primary care physicians to perform the *case coordination* function and address the wider needs of patient and caregiver. These nurse practitioners carry out physical examinations and are allowed to prescribe containment products and, in future, medicines. A trained continence nurse's expertise will be useful in advising on and prescribing containment products based not just on absorbency criteria but also taking account of user-, product- and usage-related factors (see section 4.3.6). Their nursing training will help them when they liaise with home care agencies to address care needs arising from other morbidities as well as non-medical care needs. In more complex cases they can consult with continence nurse specialists based in secondary care. There are currently few continence care nurse practitioners, but with 350 continence nurses in the field it is likely that a significant proportion would be interested in extending their training to include continence related physical examination and prescribing.

Considering the payers' interest in procuring high quality care, these organisations should have the capability to specify certain aspects of providing a good continence service as in the case of the United States (see above). By ensuring efficient and effective early assessment and conservative approaches before referral to secondary care cost-effectiveness can be ensured over the whole care pathway, helping to lessen resources spent through the inadequate management of patients.

With regards to the *use of technology*, continence care services in the Netherlands can learn from the success of ParkinsonNet: an innovative IT-enabled network for sharing of best practice amongst healthcare professionals. Other potential uses of technology are recommended and set out in section 4.3.7.

United Kingdom (current)

The UK has an essentially socialised healthcare system currently operating under the single payer 'Beveridge' model and delivered by the National Health Service (NHS). However, the payer function is mostly split over 200 local organisations called *clinical commissioning groups* (CCGs) each led by representative primary care physicians and managers. These CCGs pay *providers* on behalf of the local population to deliver healthcare services. CCGs will usually pay providers for continence care through a *block* contract i.e. a flat payment to cover delivery of services over an agreed period of time with no adjustments for levels of activity. Because care is provided through a block contract and is usually oversubscribed, there is a disincentive to 'over-promote' services even where there is unmet need, as these services would quickly become overwhelmed - thus a form of healthcare rationing is built into the service.

In some areas, commissioners make use of a quality-related financial lever called a *CQUIN (Commissioning for Quality and Innovation)* target. By achieving a CQUIN target providers can receive a payment as a reward for high-quality care e.g. percentage of patients with stress incontinence offered pelvic floor exercise training. CQUINs are locally agreed between commissioner and provider – although there are a small number of nationally-mandated CQUIN targets of which none are

continence-related. In the Continence Care Survey 2013, 27% of respondents said there were locally-agreed CQUINs (40% did not know). Primary care physicians are also influenced by financial incentives. To optimise their income, primary care physicians must ensure that they achieve a number of targets in their clinical practice across over 100 outcomes indicators on the *Quality and Outcomes Framework*. Continence care is not included in this framework and consequently it finds itself nearer the bottom of the list of priorities of a primary care physician. Funding for continence care remains static across the NHS.

Despite Department of Health guidance specifying providers to deliver integrated services, consecutive National Continence Care Audits (2005, 2006 and 2010) indicated that in many areas this does not occur. There are some good examples of multi-disciplinary care pathways which look after the care of the patient more holistically, but this is not the norm. The Continence Care Survey in 2013 suggested that the situation has not improved although there is some evidence of increasing sophistication of services in some areas including more multi-disciplinary team working, development of clinical pathways and improved awareness of, and access to, services. England appears to have greater barriers to integration than Wales and Scotland, where there is no commissioner – provider split. Nurses in Wales report feeling very much like 'playing for the same team' when working across organisational boundaries, while the purchaser-provider split in England appears to encourage territorialism.

Levels of detection of incontinence are low. Primary care physicians have been shown to underestimate the level of the problem: many do not ask about lower urinary tract symptoms or consider them an inevitable element of ageing, and many are unaware of locally available services. As explained above, continence care is often given a low priority compared with other disease areas. Due to a combination of under-detection in primary care and poor awareness of services amongst healthcare professionals and sufferers, those with incontinence often struggle on in silence. Some are able to train themselves to perform pelvic floor exercises, particularly in the post-natal period. The promotion of self-care for incontinence could be improved. Patient advocacy organisations such as the Bladder and Bowel Foundation could help fill the gap.

Patients can undergo *initial assessment and treatment* with their primary care physicians and be referred on to a trained 'continence nurse advisor' or physiotherapist. Successful nurse-led services receive very little input from physicians during the initial phase of treatment. Nurse continence advisors are masters-level educated with some deeper knowledge of the use of specialist investigations and treatment including indications for upward referral. In some services, patients may be referred to a specialist centre but be assessed, treated and discharged without seeing a doctor.

Technology has the potential to play a key role in enabling the delivery of integrated services but the lack of development in this area is currently a barrier. Patients moving between different healthcare settings e.g. community nursing care to hospital care to primary care will have their documentation held on multiple IT systems, none of which interfaces with each other. Use of technology to monitor patients remotely or to help reinforce behavioural interventions is patchy at best.

Care products are, to a certain extent, provided by continence care services (i.e. they are not out-of-pocket). According to the 2013 Continence Care Survey, 15% of continence care services have a waiting list (up from 7% in 2007). Up to half have experienced an increase in the number of patients requiring products while the budget for pads has remained roughly constant. 82% of survey respondents said that they provide products based on clinical need. However, most services have a maximum allowance for the number of pads supplied per 24 hours. According to the survey this allowance varies significantly between 3 products - 6% (of services), 4 products - 68%, 5 products - 23%, and 8 products - 3%.

United Kingdom (optimum)

In the UK, there is a need for patients to be seen at the right time by the right professional. There also needs to be better multi-disciplinary working to ensure patient-centred care that maintains patient (and caregiver) quality of life and places a priority on enabling self-care.

There should be less emphasis on care by medical practitioners and more involvement of nurse specialists delivering conservative treatment strategies as part of the *initial assessment and treatment* component. This is especially important in order to reduce the number of referrals to specialists. As part of the Leicestershire MRC Incontinence Study, nurses were trained to lead a continence service and provide conservative treatments: the outcomes have been shown to be good in the short and medium term, with patients more appreciative of some aspects of care. In view of these all-round skills UK continence nurse specialists would seem particularly well suited to performing the role of *case co-ordinator.*

Nurse specialism is a well-established concept now in the UK, not least in specialties such as elderly care and neurology (e.g. the multiple sclerosis nurse specialist). It will be important to avoid duplication of care, and continence nurse specialists will need to reinforce the primacy of the role of their counterparts who may have a wider perspective on a patient's care needs, and who therefore should be the key interface between the patient, caregiver, social care and medical specialist (e.g. neurologist).

In those areas where there is no dedicated nurse-led continence service, primary care physicians need in the meantime to be better trained to deal with continence problems including *initial assessment and treatment*, patient education and appropriate referrals to specialist doctors and/or physiotherapists for *specialist assessment and treatment*. One alternative to this may be an augmentation of the role of district (community) nurses, who already work in primary care, to include continence care.

There also needs to be greater awareness of local services. The saturation of local services may help to drive the case for further investment in continence care, which will in turn increase availability of services and reduce the level of unmet need. This can potentially be achieved through more co-ordinated working with patient advocacy organisations and 'third sector' organisations dedicated to incontinence, women's health issues or care for the elderly.

CQUINs (see current service configuration) could be agreed locally between commissioners and providers to set targets for the quality of care according to specified outcomes including reduction in admissions related to incontinence and the reduction in the prevalence of in-dwelling catheters. While it is unlikely that continence care will be included in the Quality and Outcomes Framework in the near future, clearly linking the achievement of continence-related quality outcomes to payment could raise the profile of continence care amongst primary care physicians in the UK. Patient-reported outcome measures (PROMs) and patient-reported experience measures (PREMs) are likely to become increasingly important in the measurement of service quality in the UK: performance incentives could be usefully linked to these measures.

The clinical audit tool published with the most recent UK National Institute for Health and Clinical Excellence (NICE) guidelines for urinary incontinence for women [98] could be usefully deployed to monitor clinical practice against the guidelines.

India (current)

Compared with the other healthcare systems featured, India's health system is highly fragmented, lacking regulation and organisation. As a result, the needs of people with incontinence are neither well recognised nor well met. A combination of generally poor access to healthcare across the population together with limited availability of healthcare professionals able to deliver continence care means sufferers often have to travel far (usually to the nearest big city) to receive the care they need, and when they do, patients usually have to pay for a high proportion of their care out-of-pocket.

In general, healthcare services are governed by the National Health Policy (2002), with a government contribution of 0.9% of the GDP. The rest of the 4.2% of GDP healthcare spend comes from the private sector, mostly out-of-pocket. The policy is largely focused on communicable diseases and healthcare services for those living below the poverty line, both of which are the responsibility of the government. There are some select programmes for the elderly, women and children covered by

different initiatives run by the Ministry of Health and Family Welfare (Health initiatives)., Commission for Women & Children (Women and Child development initiatives) and Ministry of Labour & Employment (social health insurance initiatives) Sufferers with incontinence are largely expected to 'seek their own care'. Treatment and management decisions are based on a combination of clinical factors and consideration of affordability to patients.

The private sector drives healthcare service delivery and is estimated to cater to about 80% of the population by 2020. Secondary and tertiary care, and to an extent primary care is largely based in urban areas. Rural India largely depends on alternative therapy practitioners or doctors belonging to other streams of medicine (homeopathy, *unani*, *siddha*, *ayurveda*) to provide primary care. A large section of urban India also uses the services of these doctors due to issues related to affordability and access.

The National Rural Health Mission (NRHM) was created to integrate various siloed programmes under a single umbrella, thereby improving collaboration across initiatives, and improving community involvement for the rural population. The implementation of the program has been patchy, due to over-ambitious goals and a lack of political will, although the government has envisioned a nationwide National Urban Health Program to cater for the urban poor. Most of these initiatives are directed towards the more vulnerable sections of society including the poor, the elderly, women and children. These initiatives focus on current national health priorities namely infant mortality, maternal mortality, vector borne disease, tuberculosis etc. However, urinary incontinence is not considered a health priority and there are no specific programmes for urinary incontinence currently operating in India, although there are NGOs that have been working in this area.

The public sector network is structured to provide primary care at first level of contact, with increasing levels of specialised care, moving up the referral chain. Unfortunately, the lack of access and availability of resources at the primary care level have resulted in a strain on higher levels within the referral chain. Current estimates suggest that only about 20% use healthcare services in the public sector. Public sector services are provided at,

1. *Primary Healthcare centres* (*PHCs*, rural) or *Urban Health Posts* (*UHPs*, urban), typically for a population base of about 30,000 - 50,000. PHCs are manned by generalist doctors along with nurses and other staff.

2. *Community Health Centres* (*CHCs*) provide services at sub-district level including 5 core specialities, gynaecology, surgery, general medicine, paediatrics and orthopaedics (option). They are typically 30- bed facilities which cater to a population of up to 500,000.

3. *District hospitals* are larger facilities about 100-bed units which cater to the entire district. They have a wider range of specialists positioned here.

4. *Teaching hospitals* (usually government run) are 500- to 800-bed facilities. They house practically all specialties (including urology and gynaecology) which may be relevant for the local population. These units are usually oversubscribed with patients.

Private health insurers would typically cover 70% of costs incurred for surgical and hospitalised care. Currently, there is no coverage for investigations, consultations, medications and products purchased as part of outpatient consultations, although efforts are underway to pilot test some form of ambulatory care coverage by private and social insurance programmes.

Care is not well integrated, and in most cases, patients do not know where to seek it. Most patients arriving in government teaching hospitals providing tertiary care are usually complicated or deteriorating cases. These patients are also often the poorest and least well-informed.. In the absence of an integrated pathway and lack of specialists (estimates suggest a ratio of 1:500,000 urologists to patients) deserving patients do not receive appropriate specialist medical care. At the same time, most specialists end up treating the full spectrum of presentations from simple cases requiring more conservative interventions to more complex cases requiring specialist opinion and possibly surgery.

The level of awareness of continence issues is poor, as sufferers generally do not consider incontinence to be a medical problem. Female and older sufferers continue to suffer for a long time and tend to only seek medical help when the situation has deteriorated or becomes intolerable. If they do then seek medical help, they may not know where to go: well-informed sufferers and those with financial means are more likely to have a medical consultation. For those frail elderly sufferers not seeking help, there is often a transfer of the burden onto caregivers, usually family members. There are no specifically trained caregivers but a family may have a general domestic assistant who can assist in preventing bed sores and urinary tract infections in bed-ridden patients.

Patients purchase containment products out-of-pocket, and are provided guidance by physicians on which product will best suit individual patients' needs. A crucial concern for patients is price, which depends on prevailing market trends and government regulations. Only products used during hospitalisation are covered by providers, and in most cases these consumables have a high co-payment. In rural India, clothing is typically used in place of containment products.

India (optimum)

Owing to the current limited provision of continence care in India and 'bare bones' healthcare infrastructure across large swathes of the country, the combination of challenges is significantly different compared with the other country examples.

There is a need to train a new cadre of staff to improve access. Targeted training in continence care will be necessary for a variety of healthcare professionals. Owing to the lack of qualified nurses and the lack of precedent for nurses to provide care in substitution of doctors, the nurse specialist model is not viable. In India, nurses work under the supervision of the doctor and have limited scope for more autonomous roles. Training MBBS (degree-educated) doctors to provide basic treatment will improve adherence to evidence-based clinical guidelines and will also be helpful in bringing continence care to the forefront of the minds of physicians. However, even an increased emphasis on training existing healthcare professionals will be extremely challenging given the competing priorities on physicians' time in India's underdeveloped healthcare system.

An alternative is to train willing community healthcare workers (i.e. healthcare workers with no formal qualifications) in the basic knowledge and practice of continence care. These trained community healthcare workers will be expected to provide the knowledge and insight required to help patients to self-care, without the benefit of scarcely available tests and investigations. To some extent they will provide the *initial assessment and treatment* component. These trained community healthcare workers will also be expected to identify those patients who would benefit from *specialist assessment and treatment* which, for the majority of the Indian population that lives in rural areas, would usually require patients to travel to urban areas. It may be that the most effective way to train local community healthcare workers will be via the creation of satellite clinics run by teaching hospitals. Periodic exposure of these workers to specialists may help to disseminate basic continence care best practice amongst peers in neighbouring areas. *Case co-ordination* may not be a realistic component in India, but healthcare professionals providing continence care need to communicate effectively with patients and caregivers alike if the latter are to receive the best quality of treatment.

A further option is to make use of the large numbers of alternative medicine practitioners. These practitioners constitute an existing infrastructure with good access to patients, and they could be trained to perform case detection and a basic initial assessment and treatment.

Awareness amongst sufferers of the presence of specialised services is important: there needs to be a push to raise societal awareness of incontinence as a medical problem and to remove the stigma associated with this condition in India. This potentially requires a sustained promotional campaign in order to raise the issue with the public, patients, healthcare professionals and health system administrators. One of the biggest challenges will be to spread the message to people in non-urban areas who are not easily reached.

Affordability could also be enhanced by providing national insurance cover. Public hospitals can help improve outcomes by increasing access in rural areas. However, most doctors prefer to be in urban localities. The provision of containment products is a key issue, as this represents a significant cost to patients when purchased. It is particularly important to provide effective containment products to those who use their own clothes for this purpose. These patients may be particularly socially restricted with profound effects on quality of life.

The use of technology is an untapped avenue of opportunity for augmenting continence care. While most Indians will not have access to the internet nor to smart mobile telephones, many will have access to simpler mobile technology. As mentioned in section 4.3.7 , prevention, surveillance, self-management and compliance is well established in developing countries, particularly in parts of Africa.

Summary

By aligning incentives, investing in the number of continence nurse specialists, instituting training for other appropriate healthcare workers where there are no trained continence nurses available, but while remaining realistic about local resource constraints and healthcare systems, we believe the *optimum* future service configurations may resemble the Table 1 below:

Table 1: Optimum service configuration for the US, the Netherlands, the UK and India

Component	United States	Netherlands	United Kingdom	India
**Case detection**	Primary care physician/ Specialist doctor/Continence nurse specialist	Continence care nurse Practitioner	Primary care physician/ Community nurse/ Specialist doctor	Trained community healthcare worker
**Initial assessment and treatment**	Continence nurse/Advanced practice continence nurse	Continence care nurse Practitioner	Continence nurse specialist	Trained community healthcare worker
**Case co-ordination**	Continence nurse/Rehabilitation or Geriatric nurse	Continence care nurse practitioner/ Neurological or Care of the elderly specialist nurse	Continence nurse specialist/ Neurological or Care of the elderly specialist nurse	May not be possible
**Caregiver support**	Continence nurse specialist/ Neurological or Care of the elderly specialist nurse	Continence care nurse practitioner/ Neurological or Care of the elderly specialist nurse	Continence nurse specialist/ Neurological or Care of the elderly specialist nurse	May not be possible
**Community-based Support**	Community social worker/ Third sector	Home care organisation/ Third sector	Community social worker/ Third sector	Community social worker/Third sector
**Specialist assessment and treatment**	Specialist physician/ Advanced practice continence nurse/Physiotherapist (treatment only)	Specialist doctor/ Physiotherapist	Specialist doctor/ Physiotherapist	Specialist doctor/ Physiotherapist
**Use of containment products**	Continence nurse/Advanced practice continence nurse	Continence care nurse Practitioner	Continence nurse specialist	Trained community healthcare worker
**Use of technology**	Telehealth, Online educational tool, Patient/Professional networks	Telehealth, Online educational tool, Patient/Professional networks	Telehealth, Online educational tool, Patient/Professional networks	Telehealth

5.2 Patient profiles

For the purposes of testing the service specification from the perspective of the patient, the expert panel identified four different profiles of patients requiring care for incontinence:

- stress and urgency;
- faecal incontinence;
- neurological;
- elderly/cognitively impaired.

Each profile of patients has its own specific health and social care considerations, including specific clinical guidelines [1, 14]. The patient profiles also vary in their typical requirements for containment products. Below we set out which aspects of the service specification will apply to each profile of patients and show how the service would ensure completeness of care.

5.2.1 Stress and urgency

This profile of patients consists of two subgroups, *stress and urgency.* The *stress* group includes especially those younger and middle-aged women affected by stress incontinence, often during pregnancy and after childbirth. The *urgency* group refers to patients with urgency incontinence and mixed urinary incontinence. This includes especially those middle-aged women affected by bladder and/or pelvic floor problems and middle-aged and older men with urgency incontinence. These patients tend to suffer from higher volume leakage episodes [3].

Stress and urgency cases should be detected by the main primary care contact. usually the primary care physician, where they should be referred on to a dedicated nurse-led continence service if available (see section 4.3) for *initial assessment and treatment* and *case co-ordination*. It should also be possible for this profile of patients to self-refer to a dedicated nurse-led continence service.

A subgroup of patients will require immediate referral to specialist services – according to established guidelines [1]. More straightforward cases will undergo initial assessment and treatments including lifestyle/behavioural interventions e.g. dietary advice, bladder training, pelvic floor exercises. The case co-ordinator will ensure treatment is timely and appropriate throughout for the patient’s specific care needs, prescription of containment products and be available to answer any queries regarding their care [1].

If, following a course of treatment, symptoms do not improve to the satisfaction of the patient, he/she will be referred on to the relevant specialist in alignment with established guidelines [1].

Figure 3: Typical set of service components applicable to a woman with *stress or urgency* incontinence

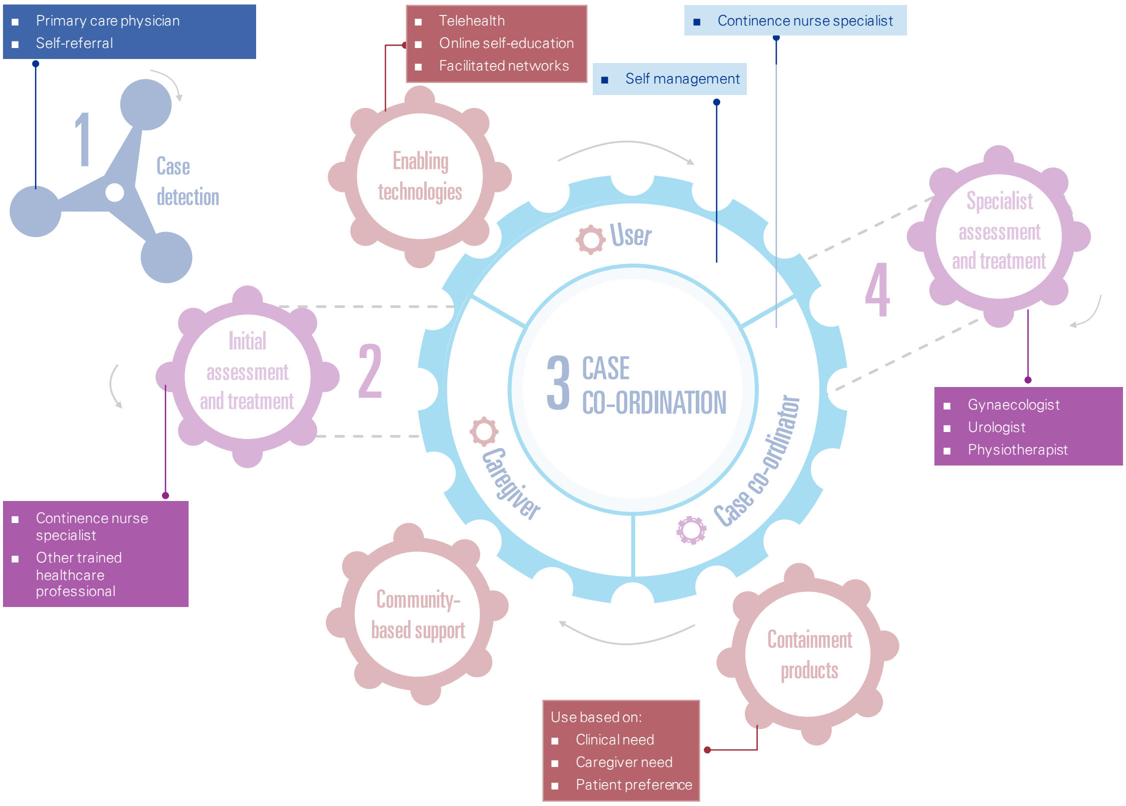

5.2.2 Faecal incontinence

This profile of patients includes men and women with faecal incontinence and covers all ages.

Faecally incontinent patients, like those with urinary incontinence will usually be detected by the main primary care contact e.g. primary care physician who will refer on to a dedicated nurse-led continence service for *initial assessment and treatment* and *case co-ordination*. Also like *stress and urgency* patients, faecally incontinent patients should have the option of self-referral to a dedicated nurse-led continence service. As per accepted clinical guidelines, patients with 'red flag' symptoms must be referred on to a specialist doctor for immediate investigation [1, 9]. *Initial assessment and treatment* can otherwise be carried out by the dedicated nurse-led continence service. More straightforward cases will undergo initial treatments. The case co-ordinator will ensure management and treatment is timely and appropriate throughout the patient's care journey and be available to answer any queries regarding their care [1, 3].

If, following a course of treatment, symptoms do not improve to the satisfaction of the patient, he/she will be referred on to the relevant specialist. Likewise, patients with a clear indication for surgical evaluation will be transferred into the care of a relevant specialist able to deliver the range of investigations and treatments as specified in the relevant guidelines for specialist management [1].

Figure 4: Typical set of service components applicable to a male *faecal incontinence* patient

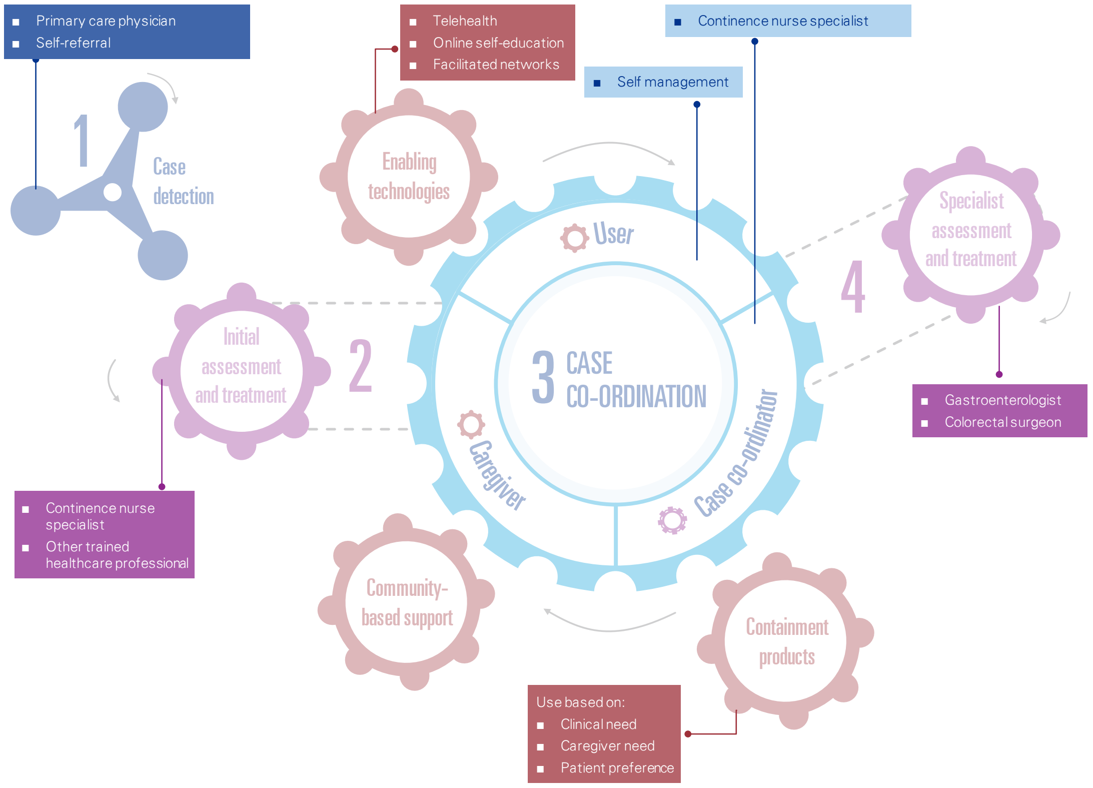

5.2.3 Neurological

The *neurological* group includes patients with a variety of neurological conditions such as multiple sclerosis, motor neurone disease and different forms of paralysis [3]. They can have urgency or sphincter dysfunction with/without faecal incontinence [3].

In most cases, referral to continence care will be activated by their neurology specialists or specialist neurology nurses e.g. multiple sclerosis nurse specialist. These patients will usually need joint management by the (continence) specialist physician and nurse specialist. Patients often need evaluation to differentiate neurogenic bladder from non-neurological pathologies. Most patients with neurogenic urinary incontinence require specialist assessment with urodynamic studies and/or renal tract imaging. Treatment is usually conservative for both urinary and faecal incontinence but may involve surgery for some patients.

Figure 5: Typical set of service components applicable to a patient with multiple sclerosis

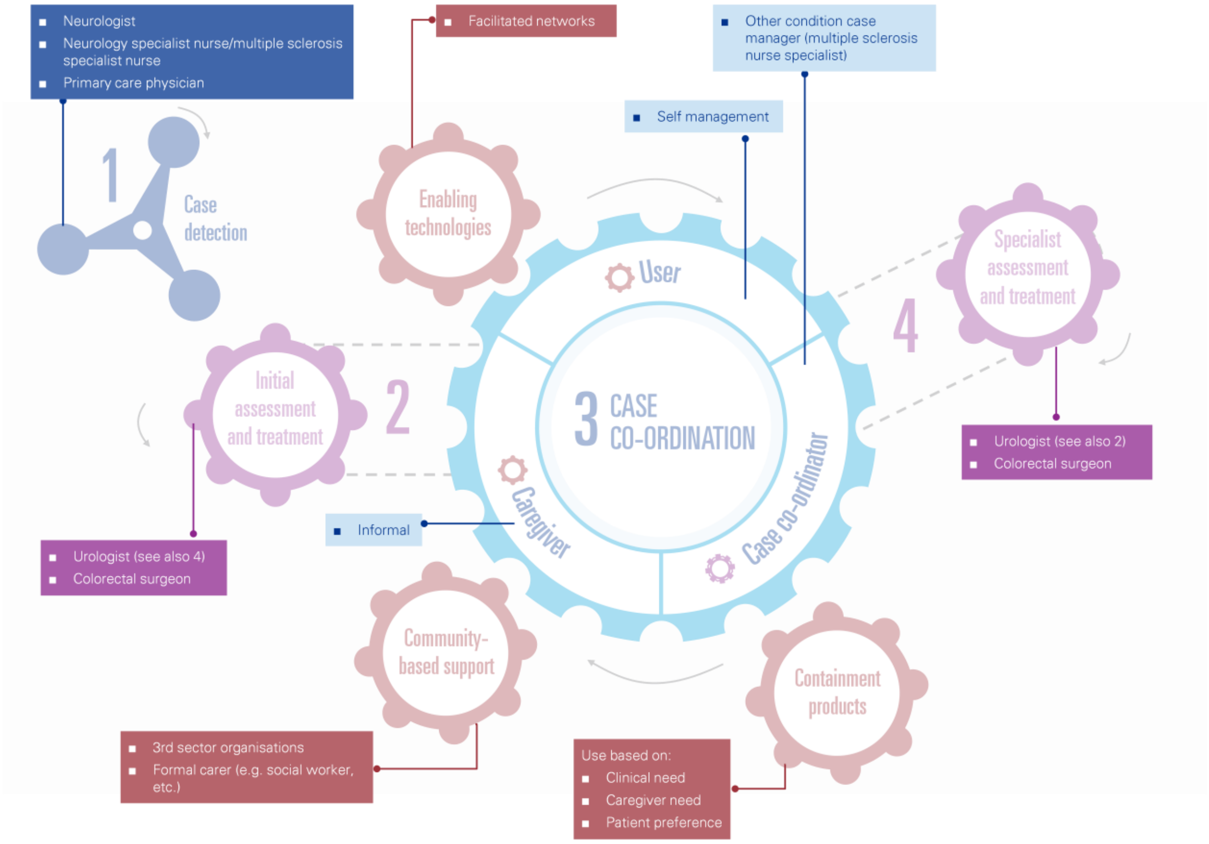

5.2.4 Elderly/cognitively impaired

Elderly/cognitively impaired patients are characterised by functional impairment and/or cognitive problems. Healthy elderly with full, active lives with few care needs should be managed in the same way as *stress and urgency* patients or *faecal incontinence* patients. UI and FI in frail older people are each normally a result of the interaction of multiple concurrent risk factors, including age-related physiological changes, multi-morbidity, and polypharmacy [14]. Co-morbid illnesses in the elderly include dementia, Parkinson's disease, diabetes or the effects of complex medication regimens. *Elderly/cognitively impaired* patients may be affected by urinary or faecal incontinence and often both [3].

These patients will typically be identified by a primary care physician or a specialist elderly care physician/ nurse. *Case co-ordination* in this group is usually best undertaken by a specialist elderly care nurse or other healthcare professional who is experienced in managing the frequently complex mix of health and social care needs. Dedicated nurse-led continence services would play a facilitatory role – providing advice and information for the case co-coordinator on usability of containment products in certain care situations – and will also provide the *initial assessment and treatment*.

Elderly/cognitively impaired require tailored management and treatment plans according to the effect on patient/caregiver QoL, the presence of co-morbid illnesses, patient/caregiver expectations and goals, remaining life expectancy, the likelihood of concordance and ability to tolerate treatment [14]. The basis of management for many patients will be containment or management of 'contained incontinence'. In this state, patients can remain dry with the judicious use of toilet assistance, containment products, behavioural interventions and/or medications [14]. More complex cases or patients who fail initial treatment may require referral for specialist assessment from a surgical specialist, care of the elderly physician or physiotherapist [1].

An important aspect for *elderly/cognitively impaired* patients is that non-clinical care needs may be mostly met by informal caregivers. These caregivers must be supported in a variety of ways to ensure they are able to cope with the burden of caring for a frail older person with incontinence: These include distribution of information and advice on the nature of the disease and the usability of containment products as well as timely respite care. Some patients with untreatable incontinence superimposed on multiple health and social care needs will be given more intensive formal home care or they or their family/caregiver might decide on a move into a residential care facility. Adequate care planning and the involvement of all relevant stakeholders in related decisions and 'handovers', e.g. from home care to a nursing home, has to be ensured as changes of living environment may have a detrimental impact on this group.

Figure 6: Typical set of service components applicable to an *elderly/cognitively impaired* patient

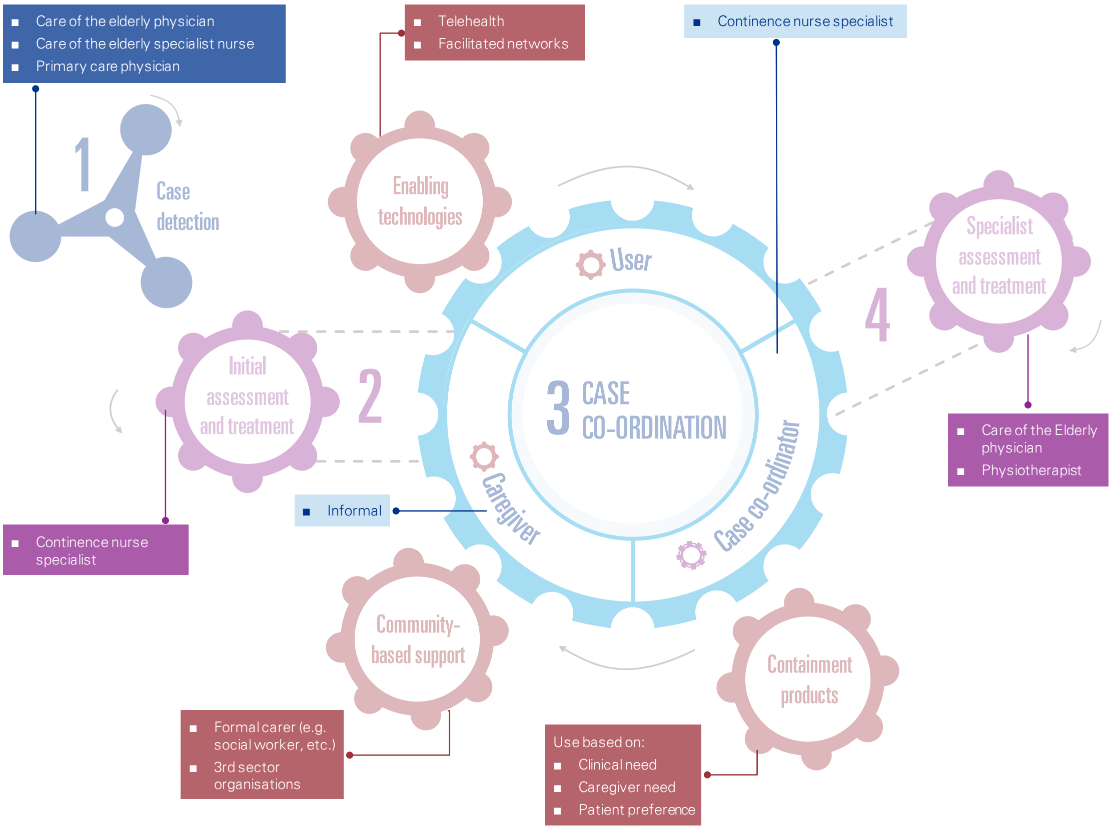

6 Measuring quality and service performance

6.1 Adding value: a focus on outcomes

It is important to decide which of the healthcare benefits ought to be considered 'required outcomes' from the service provided as these can be used to judge the effectiveness of a service. Carefully-picked outcome indicators can be used as powerful levers to focus the attention of providers on providing the highest quality care. There are several areas of outcome indicators that we would recommend to focus on in order to measure the quality of care:

- symptom relief: e.g. episodes of leakage, nocturia, urgency [99];
- clinical safety e.g. reduction in rate of complications such as falls, UTIs, skin damage;
- reduction in effect of incontinence on quality of life for patients and caregivers e.g. improvement in EQ-5D score, re-employment rates, re-ablement [99];
- patient experience of care e.g. patient rating of communications, treatment outcomes versus expectations/goals, positive emotions and relationships with staff [100];
- appropriate use of containment products: e.g. freedom of choice, provision based on holistic assessment of patient and caregiver needs, use of a standardised assessment tool, availability of individual patient-level product testing/sampling.

We recommend regular systematic clinical audits to ensure the adherence of clinical care to evidence-based guidelines.

6.2 How to use outcome indicators

There are a number of ways to use outcome indicators to promote the highest quality care. One of the most important is to increase the transparency of these indicators so that patients and payers can see how each service is performing. This is usually a powerful motivator for healthcare providers looking to ensure that they are providing the best possible care for patients. We recommend sharing outcomes data in the public domain and reporting of results to internal and external stakeholders including patients, staff, payers, associated services and health system administrators. All stakeholders should be enabled to use results to reflect upon interventions and measures for continual improvements.

Another way to use outcome indicators is to link financial incentives to the achievement of certain targets e.g. 85% of patients are satisfied with care received, or less than 2% of local hospital admissions associated with incontinence. However, as long as evidence-based outcome indicators in incontinence are rare, it is important not to overburden providers with financial targets as this can distract the attention of healthcare professionals from the task of providing comprehensive evidence-based care on a patient-by-patient basis. It may be appropriate therefore to use financial incentives in a targeted way to encourage pathway compliance rather than using them as an on-going system of monitoring.

6.3 Performance measures

Performance measures are useful to help understand whether a service is functioning well. Certain changes or trends in these indicators may indicate a service that is under strain or uncontrolled. Data need to be discussed and analysed regularly by team members to develop, if appropriate, adequate steering activities to counteract negative trends.

Examples of performance measures include:

- proportion of at-risk patients screened;
- ratio of patients per trained staff member;
- number of patients seen, treated and discharged by the *appropriate* professional at each stage of pathway;
- waiting times for access/treatment;
- total costs of care per patient per year;
- rates of appropriate referrals for specialist investigations e.g. urodynamics, flowmetry studies.

KEY RECOMMENDATION
For payers: in order to provide the highest quality continence care, ensure care standards are incentivised. This can be achieved through stipulating the achievement of targets on certain outcome and operational measures, careful use of quality-related financial incentives, and an emphasis on clinical governance.

7 Implementation considerations

Providing a truly integrated service for continence care will likely require development in a range of areas across a healthcare system. There will be implications locally, regionally and nationally for:

- *Training and education of current healthcare professionals:* for nurse specialists to play a more prominent role in continence care will require a substantial programme of training of existing nurses even in systems which have relatively high numbers of continence nurse specialists. In particular we recommend the establishment of a certificate in continence care nursing to provide assurance on quality of practice of new and existing continence nurse specialists [33]. In section 8, we share some examples of the roles and expected level of training of nurses currently delivering continence care in the US, the UK and the Netherlands.

  Such a training programme will require substantial investment on the part of those organisations responsible for professional training and will likely take several years to bring continence nurse specialist numbers to the required level. A significant part of training will necessarily involve 'on-the-job' experience, and this will require educational institutions working effectively together with provider organisations.

  The most significant barrier to implementing this model may be found in those healthcare systems in which it is not usual for nurses to take on additional clinical responsibilities such as making the initial assessment, and starting treatment, especially without supervision. In these systems it may be necessary to focus only on educating and training the appropriate medical staff (see below).

  In the short-term, in almost all healthcare systems, there will need to be a focus on the training of existing healthcare professionals including generalist and specialist physicians, specialist physiotherapists, generalist nurses and existing continence care specialists (see above) [33]. We also recommend empowering existing 'nursing champions' to help diffuse best practice within existing generalist nurses [43, 101].

  > ***KEY RECOMMENDATION***
  > *Establish accredited programmes of training for 1) nurses wanting to become continence nurse specialists, and 2) other health or social care professionals such as social workers wishing to improve their competence in delivering continence care.*

- *Workforce planning:* to train sufficient numbers of additional nurses or other appropriate healthcare professionals to specialise in continence care in the medium to long term.
- *Continence care promotion:* to ensure sufficient healthcare seeking behaviour to produce the health and economic benefits that come from providing a high quality continence care service. Continence care promotion requires a combination of a heightened profile of incontinence as a medical problem, reduction in the stigma of incontinence, and raised awareness of available services. Services need to work together with public health teams and continence advocacy groups to ensure potential benefits of continence care are realised [33].
- *Patient and caregiver involvement:* development of services should involve greater participation of patients and caregivers: to be more sensitive to patient/caregiver needs, capabilities and preferences.
- *Payer involvement:* to fund and actively procure the highest quality services for their patients according to our suggested service specification. Payers should use the levers available to design contracts in a way that encourages providers to meet the continence care needs of the population. Depending on the healthcare system there will be many different means to achieve this – including financial risk transfer [102], pricing more aligned to the real cost of delivering services [56], use of quality/outcome-related payment incentives [78], operational performance targets [78], and regulatory levers.

- *Working across health and social care boundaries:* case co-ordination in continence care requires collaboration between providers of health and social care, particularly in those with multiple care needs such as elderly and disabled patients Such collaborative working will be easier in socialised systems where health and social care providers are funded from the same source, but can be extremely challenging in more fragmented systems. In such situations, health and social care payers may collaborate to contract with specialist 'integrator' organisations which can usefully act as 'middlemen' [102], co-ordinating multiple providers while taking on the risk and administrative burden from payers.
- *Procurement of containment products:* to ensure availability of the correct products and to uphold the principle of patient choice we recommend segmenting patients into the patient profile categories in section 5.2 when procuring products. For more unusual requests and needs it may be necessary to use a system of 'top-up' patient co-payments to supplement the basic package provided by the service. This solution should ensure the best possible solution in terms of effectiveness, efficiency and satisfaction for patient and caregiver.
- *Data collection:* to ensure outcomes data and performance measures are reliably recorded for the purposes of monitoring and evaluating continence services.

8 Continence nurse specialist profiles

The term *continence nurse specialist* refers to a variety of different roles and associated levels of education in nurses specialising in continence throughout the world [103]. In countries such as the United Kingdom, New Zealand and Australia, two of the most used models are the *nurse continence advisor* (or *NCA*) and the *continence nurse advisor* (or *CNA*). Both models involve generalist nurses with specialised training in incontinence. Further training at master's or doctorate level could lead to the designation of *continence nurse practitioner* (*CNP*). In section 4, we recommend a continence nurse specialist be responsible for certain elements of continence care: she/he could be either generalist nurses with specialist training in continence or nurses with more advanced training.

Table 2 outlines a variety of profiles for continence nurse specialists in the US, the UK and the Netherlands.

Table 2: Example current profiles of continence nurses

Country	Designation	Education / Licensure	Incontinence Care Delivered
**United States [100]**<br>**(WOCN Board of Directors Position Statement, 2009)**	Continence Nurse	WOCN certification<br><br>Eligibility<br>RN<br>Baccalaureate degree<br>Completion of accredited WOC program<br>Completion of graduate level program with WOC coursework<br>Had 50 contact hours and 1500 clinical hours with continence patients within past 5 years	■ Performs a focused assessment<br>■ Obtains a relevant history<br>■ Performs bedside testing of bladder filling and sensation (bedside cystometrogram)<br>■ Measures post void residual urine by catheterization or bladder scan<br>■ Synthesizes data related to incontinence to identify individuals at risk, reversible causes, types of urinary incontinence, and common bowel dysfunctions that contribute to faecal and/or urinary incontinence<br>■ Makes an appropriate nursing diagnosis of urge, stress, mixed, and/or functional urinary incontinence<br>■ Uses/recommends appropriate management strategies including the following interventions:<br>■ Educates and counsels patients, families, and staff regarding<br>✓ behavioural therapies such as toileting programs,<br>✓ urge suppression, and pelvic muscle exercises;<br>✓ bowel training or stimulated defecation programs;<br>✓ intermittent self catheterization;<br>✓ care of indwelling urethral and suprapubic catheters;<br>✓ incontinence products;<br>✓ measures to clean, protect, and moisturize the perineal skin;<br>✓ treatments for incontinence-related skin breakdown; and<br>✓ fluid and dietary modifications.<br>■ Monitors therapeutic effects of medication therapy<br>■ Provides pelvic floor rehabilitation and re-education via electrical muscle stimulation and biofeedback in some settings<br>■ Evaluates outcomes of interventions and

			reports to the primary care provider as appropriate<br>■ Makes appropriate referrals for recurrent urinary tract infections, haematuria, pelvic organ prolapse, urinary retention, and pelvic pain syndromes
	Advanced Practice Continence Nurse	Master's level education<br><br>Eligibility<br>RN licensure<br>Completion of graduate level program as NP or CNS<br>Graduate of accredited WOCN program<br>Holds CWOCN	■ Monitors overall quality of care to identify needs for improvement<br>■ Performs a comprehensive physical assessment that may include a pelvic examination for masses, prolapse, and urethral hypermobility; a digital rectal examination of the prostate and a neurologic assessment<br>■ Synthesizes data<br>■ Uses/recommends appropriate management strategies including the following interventions:<br>– Interprets diagnostic studies such as urodynamic studies and studies of bowel motility and elimination<br>– Prescribes pharmacologic treatment for common conditions of the urinary tract and bowel such as urinary tract infection, overactive bladder constipation and diarrhoea<br><br>■ Provides care for common gynaecological condition such as vaginitis and pelvic organ prolapse (i.e. fitting and management of pessaries)<br>■ Performs complex, multichannel urodynamic studies with/or without fluoroscopic imaging<br>■ Performs anorectal manometric studies<br>■ Provides pelvic floor rehabilitation and re-education via electrical muscle stimulation and biofeedback
**United States [103]**<br>**(Adapted from Newman, D.K. (2006). *The roles of the continence nurse specialists.* in Cardozo, L., Staskin, D. (Eds) *Textbook of Female Urology and Urogynecology.* 2nd Ed. Isis Medical Media, LTD. United Kingdom: 91-8)**	Advanced Practice Nurse<br>(APN)<br><br>Nurse Practitioner (NP)<br><br>Clinical Nurse Specialist<br>(CNS)	Master of science in nursing<br>Master of science<br><br>Certification though the American Nurses Credentialing Center<br><br>Meets individual State requirements for advanced practice nurse	■ Identification of UI<br>■ Basic assessment of UI to include comprehensive history, mental, functional and environmental assessment<br>■ Complete physical examination to include assessment of genital prolapse with fitting of pessary<br>■ Baseline urologic assessment<br>■ Complex urodynamics with interpretation of data<br>■ Additional studies such as laboratory<br>■ Identification of patients who need referral to other medical specialists<br>■ Education and counselling of patients/families and caregivers<br>■ Nursing interventions to include:<br>✓ Scheduled toileting programs<br>✓ Bladder retraining<br>✓ Behaviour modification

			✓ Pelvic muscle rehabilitation with biofeedback therapy and interpretation of data<br>✓ Neuromuscular pelvic muscle electrical stimulation<br>✓ Implementation of the use of devices, and products<br>■ Prescribes pharmacological intervention<br>■ Supervision of nonprofessional staff<br>■ Bladder health promotion strategies
**United Kingdom [104] (Adapted from RCN (2006). *Improving continence care for patients. The role of nurses*)**	Nurse specialist	Registered nurse	■ Order diagnostic investigations<br>■ Directly make and receive referrals<br>■ Admit and discharge patients for specified conditions and within agreed protocols<br>■ Manage patient caseloads<br>■ Prescribe medicines and treatments<br>■ Perform minor surgery and outpatient procedures<br>■ Using the latest IT, triage patients to the most appropriate health professional<br>Take the lead in the way local health services are organised and run
	Consultant nurse	Master's level educated	5 core generic elements<br>1. Expertise in the practice of nursing in their specialism – for example, within surgery, the medical ward or as a nurse practitioner in the community.<br>2. A central responsibility in the development of practice that embraces education and helps to create an environment to help staff to reach their full potential.<br>3. Proficiency in research that makes a tangible difference to patient care. Their role should ensure that research findings are implemented that will lead to improvements for patients.<br>4. The ability to provide expert advice on nursing practice, education and research at every level within an organisation.<br>5. The aptitude to exhibit real leadership, involving and motivating staff to support new ways of working that focus on quality patient care.
**Netherlands [69] (adapted from Albers-Heitner P. (2011). *Who Cares? Studying various aspects of involving nurse specialists in primary care for urinary incontinence*)**	Continence nurse specialist	BIG- registered nurse with a Secondary Vocational Qualification (MBO)<br>17 days training at the Antoniusacademie Nieuwegein over a 12 month period<br><br>BIG-registered nurse with a Higher Professional Education (HBO)<br><br>17 days training at the	■ Standardised history taking<br>■ UI diagnosis<br>■ Treatment plan<br>■ Information, education and advice on:<br>✓ Anatomy and function of bladder and pelvic floor muscles<br>✓ Good toilet behaviour<br>✓ Influence of lifestyle, mobility, co-morbidity and cognition on UI<br>■ Bladder diary and home pad test evaluation.<br>■ Bladder training.<br>■ Assessment and training of pelvic floor muscle function (not permitted to examine

		Hogeschool van Arnhem en Nijmegen over a 12 month period	actual PFM).<br>■ Advice on containment products.<br>■ Consult with GP on cases unresponsive to treatment.
	Continence care nurse practitioner	BIG-registered nurse practitioner with a Masters qualification<br>Training in somatic care and prescribing, and can follow additional job-based training under urologist / gynaecologist supervision.	■ As for continence nurse specialists, but in addition:<br>✓ Physical examination<br>✓ Prescription of containment products and, in future, medicines

9 Glossary

**accountable care organisation**	(US) healthcare organization characterized by a payment and care delivery model that seeks to tie provider reimbursements to quality metrics and reductions in the total cost of care for an assigned population of patients
**advocacy group**	organisation promoting patient interests and providing useful education, advice and assistance to patients
**advanced nurse practitioner**	See ‘nurse practitioner’
**caregiver**	individual (usually friend or family member) providing informal care to another person
**clinical guidelines**	recommendations on the appropriate treatment and care of people with specific diseases and conditions
**community healthcare worker**	(especially rural areas of developing countries) individuals with basic or no professional qualification delivering healthcare to their community
**community nurses**	individuals providing nursing care in locations outside of the hospital including the patient’s home
**co-morbid**	relating to or denoting a medical condition that co-occurs with another
**co-payment**	a supplementary payment from an individual with health insurance that is always required regardless of coverage in order to purchase certain elements of care
**Commissioning for Quality and Innovation (CQUIN)**	(England) payment framework enabling payers to reward good practice, by linking a proportion of healthcare providers' income to the achievement of local quality improvement goals
**community nurses**	individuals providing nursing care in locations outside of the hospital or permanent care setting, including the patient’s home
**concordance**	extent to which patients complete the treatments prescribed / recommended
**conservative management**	use of non-invasive treatment strategies e.g. behavioural modifications and pharmaceutical interventions rather than surgery
**containment products**	products that contain urine or faecal matter e.g. absorbent pads, catheters and sheaths etc.

**continence**	ability to control bladder or bowel movements
**continence nurse specialist**	nurses with specialist training in the area of incontinence (see section 8 for more details)
**deductible**	the amount of expenses that must be paid out-of-pocket in any insured period before a health insurer will start contributing to healthcare expenses
**disability-adjusted life year (DALY)**	a measure of overall disease burden expressed as the number of years lost due to ill-health, disability or early death
**enabling technologies**	technologies that can enhance care delivery
**evidence-based**	consistent with the most up-to-date peer-reviewed research
**healthcare professional**	individual qualified provider of healthcare
**institutional care setting**	permanent care setting e.g. residential care home, nursing home
**multi-disciplinary**	describes collaboration of staff from different healthcare professional groups e.g. nursing staff working with physiotherapists
**National Institute for Health and Clinical Excellence (NICE)**	UK public body that develops and publishes guidance on clinical practice
**nurse-led continence service**	continence service delivered predominantly by nursing staff rather than physicians
**nurse practitioner**	a nurse who is qualified to treat certain medical conditions without the direct supervision of a doctor. This may involve taking a medical history, conducting a physical examination, ordering diagnostic tests and prescribing medicines or medical devices. The extent of the role depends on local legislation. In some countries, e.g. the United Kingdom, this role may be referred to as 'Advanced Nurse Practitioner'
**open access continence service**	continence service that can be accessed directly by a patient i.e. not requiring a referral via another healthcare professional
**outcome indicator**	a measure of the outcome of care e.g. objective measure of health status from a test/investigation, patient-recorded well-being, time to recovery
**patient**	person who has sought healthcare for their incontinence

**patient-centred care**	organisation of care driven by the health and social care needs of the patient rather than the structures and/or convenience of healthcare providers
Praktijk Ondersteuner Huisartsenzorg (POH)	(Dutch) General Practitioner's assistant, with the responsibility for certain routine tasks such as regular measurement of patients' diabetes markers
**primary prevention**	prevention of incontinence before the development of symptoms i.e. through identification of known risk factors
**provider**	organisational entity delivering healthcare
**Quality and Outcomes Framework (QOF)**	system of performance management and payment of primary care physicians in the National Health Service (NHS) in England, Scotland, Wales and Northern Ireland
**referral pathway**	established route of referral from one type of care to another e.g. from a provider of initial assessment and treatment to a specialist
**registered nurse (RN)**	a graduate from a nursing program with a license to practice
**self-management**	care or treatment that is led by the patient
**short messaging service (SMS)**	text messaging service component of phone, web, or mobile communication systems
**sufferer**	person with illness e.g. incontinence
**top-up payment**	voluntary payment from an individual with health insurance to purchase more care on top of their existing coverage
**triage**	streaming of patients on accessing a service according to level and immediacy of healthcare need

10 References

1. Abrams, P., et al., *Fifth International Consultation on Incontinence Recommendations of the International Scientific Committee: Evaluation and treatment of urinary incontinence, pelvic organ prolapse, and fecal incontinence*, in *Incontinence*, P. Abrams, et al., Editors. 2013, ICUD: Belgium. p. 1895-1956.
2. Lucas, M., et al. *Guidelines on Urinary Incontinence*. European Association of Urology 2013 [cited 2014 28th Feb]; Available from: http://www.uroweb.org/gls/pdf/16052013Urinary_Incontinence_LR.pdf.
3. Milsom, I., et al., *Epidemiology of Urinary Incontinence (UI) and Lower Urinary Tract Symptoms (LUTS), Pelvic Organ Prolapse (POP) and Anal Incontinence (AI)*, in *Incontinence*, P. Abrams, et al., Editors. 2013, ICUD: Belgium. p. 15-108.
4. Lee, Y.S., et al., *Prevalence of overactive bladder, urinary incontinence, and lower urinary tract symptoms: results of Korean EPIC study.* World J Urol, 2011. 29(2): p. 185-90.
5. Ebbesen, M.H., et al., *Prevalence, incidence and remission of urinary incontinence in women: longitudinal data from the Norwegian HUNT study (EPINCONT).* BMC Urol, 2013. 13(1): p. 27.
6. Stewart, W.F., et al., *Prevalence and burden of overactive bladder in the United States.* World J Urol, 2003. 20(6): p. 327-36.
7. Coyne, K.S., et al., *The prevalence of lower urinary tract symptoms (LUTS) in the USA, the UK and Sweden: results from the Epidemiology of LUTS (EpiLUTS) study.* BJU Int, 2009. 104(3): p. 352-60.
8. Tennstedt, S.L., et al., *Prevalence of and risk factors for urine leakage in a racially and ethnically diverse population of adults: the Boston Area Community Health (BACH) Survey.* Am J Epidemiol, 2008. 167(4): p. 390-9.
9. National Institute for Health and Clinical Excellence. *Faecal incontinence: the management of faecal incontinence in adults. NICE clinical guideline 49*. 2007 [cited 2014 28th Feb]; Available from: http://www.nice.org.uk/nicemedia/live/11012/30548/30548.pdf.
10. Chiarelli, P.E., L.A. Mackenzie, and P.G. Osmotherly, *Urinary incontinence is associated with an increase in falls: a systematic review.* Aust J Physiother, 2009. 55(2): p. 89-95.
11. Hunter, K.F., et al., *Falls risk reduction and treatment of overactive bladder symptoms with antimuscarinic agents: a scoping review.* Neurourol Urodyn, 2011. 30(4): p. 490-4.
12. Booth, J., et al., *The relationship between urinary bladder control and gait in women.* Neurourol Urodyn, 2013. 32(1): p. 43-7.
13. Medical Advisory Secretariat, *Pressure ulcer prevention: an evidence-based analysis.* Ont Health Technol Assess Ser, 2009. 9(2): p. 1-104.
14. Wagg, A., et al., *Incontinence in the Frail Elderly*, in *Incontinence*, P. Abrams, et al., Editors. 2013, ICUD: Belgium. p. 1001-1100.
15. Grimby, A., et al., *The influence of urinary incontinence on the quality of life of elderly women.* Age Ageing, 1993. 22(2): p. 82-9.
16. Schultz, S.E. and J.A. Kopec, *Impact of chronic conditions.* Health Rep, 2003. 14(4): p. 41-53.
17. Gotoh, M., et al., *Impact of urinary incontinence on the psychological burden of family caregivers.* Neurourology and Urodynamics, 2009. 28(6): p. 492-496.
18. Thomas, P., et al., *Reasons of informal caregivers for institutionalizing dementia patients previously living at home: the Pixel study.* Int J Geriatr Psychiatry, 2004. 19(2): p. 127-35.
19. Cassells, C. and E. Watt, *The impact of incontinence on older spousal caregivers.* J Adv Nurs, 2003. 42(6): p. 607-16.
20. van der Veen, R., et al. *Quality of life of carers managing incontinence in Europe*. 2011 [cited 2014 28th Feb]; Available from: http://www.eurocarers.org/userfiles/file/Carers%20managing%20incontinence5%2011.pdf.
21. Appleby, I., G. Whitlam, and N. Wakefield. *Incontinence in Australia*. 2013 [cited 2014 28th Feb]; Available from: https://www.aihw.gov.au/WorkArea/DownloadAsset.aspx?id=60129543602.
22. Noimark, D., N. Steventon, and A. Wagg, *A qualitative study of the impact of caring for a person with urinary incontinence (Abstract) 39th Annual Meeting of the International*

Continence Society San Francisco, USA 29 September – 3 October, 2009. Neurourology and Urodynamics, 2009. 28(7): p. 567-935.
23. Tarricone, R. *Economics of Incontinence*. in *biennial Global Forum for Incontinence*. 2010. Prague.
24. Wilson, L., et al., *Annual direct cost of urinary incontinence.* Obstet Gynecol, 2001. 98(3): p. 398-406.
25. Moore, K., et al., *Economics of Urinary & Faecal Incontinence, and Prolapse*, in *Incontinence*, P. Abrams, et al., Editors. 2013, ICUD: Belgium. p. 1829-1862.
26. The Canadian Continence Foundation. *Impacts of Incontinence in Canada. A briefing document from the Canadian Continence Foundation*. 2009 [cited 2014 28th Feb]; Available from: http://www.canadiancontinence.ca/pdfs/impacts-of-incontinence-in-canada.pdf.
27. X-Rates.com. *Historical Exchange Rates*. 2014 [cited 2014 28th Feb]; Available from: http://www.x-rates.com/historical/
28. Statistics Canada. *Population and Demography Statistics*. 2014 [cited 2014 12 February]; Available from: http://www.statcan.gc.ca.
29. Australian Bureau of Statistics. *Population By Age and Sex, Regions of Australia, 2010*. 2010 [cited 2014 28th Feb]; Available from: http://www.abs.gov.au/ausstats.
30. Hu, T.W., et al., *Costs of urinary incontinence and overactive bladder in the United States: a comparative study.* Urology, 2004. 63(3): p. 461-5.
31. United States Census Bureau. *United States Census 2000*. 2000 [cited 2014 28th Feb]; Available from: http://www.census.gov/census2000/states/us.html.
32. The Continence Foundation of Australia. *The Economic Impact of Incontinence in Australia*. 2011; Available from: http://www.continence.org.au/data/files/Access_economics_report/dae_incontinence_report__19_april_2011.pdf.
33. Newman, D.K., et al., *Continence Promotion, Education & Primary Prevention*, in *Incontinence*, P. Abrams, et al., Editors. 2013, ICUD: Belgium. p. 1787-1828.
34. Landefeld, C.S., et al., *National Institutes of Health state-of-the-science conference statement: prevention of fecal and urinary incontinence in adults.* Ann Intern Med, 2008. 148(6): p. 449-58.
35. Horrocks, S., et al., *What prevents older people from seeking treatment for urinary incontinence? A qualitative exploration of barriers to the use of community continence services.* Fam Pract, 2004. 21(6): p. 689-96.
36. Hemachandra, N.N., L.C. Rajapaksa, and L. Manderson, *A "usual occurrence:" stress incontinence among reproductive aged women in Sri Lanka.* Soc Sci Med, 2009. 69(9): p. 1395-401.
37. Doshani, A., et al., *Culturally sensitive continence care: a qualitative study among South Asian Indian women in Leicester.* Fam Pract, 2007. 24(6): p. 585-93.
38. El-Azab, A.S. and O.M. Shaaban, *Measuring the barriers against seeking consultation for urinary incontinence among Middle Eastern women.* BMC Womens Health, 2010. 10: p. 3.
39. da Silva, L. and M.H. Lopes, *[Urinary incontinence in women: reasons for not seeking treatment].* Rev Esc Enferm USP, 2009. 43(1): p. 72-8.
40. Yuan, H. and B.A. Williams, *Knowledge of urinary incontinence among Chinese community nurses and community-dwelling older people.* Health Soc Care Community, 2010. 18(1): p. 82-90.
41. Rodriguez, L.V., et al., *Discrepancy in patient and physician perception of patient's quality of life related to urinary symptoms.* Urology, 2003. 62(1): p. 49-53.
42. McClurg, D., et al., *A multi-professional UK wide survey of undergraduate continence education.* Neurourol Urodyn, 2013. 32(3): p. 224-9.
43. Paterson, J., *Consultation, Consensus and Commitment to Guidelines for Inclusion of Continence into Undergraduate Nursing and Midwifery Curricula*. 2006, Department of Health and Ageing - Continence Programs: Australia.
44. Williams, K.S., et al., *Clinical and cost-effectiveness of a new nurse-led continence service: a randomised controlled trial.* Br J Gen Pract, 2005. 55(518): p. 696-703.
45. Imamura, M., et al., *Systematic review and economic modelling of the effectiveness and cost-effectiveness of non-surgical treatments for women with stress urinary incontinence.* Health Technol Assess, 2010. 14(40): p. 1-188, iii-iv.

46. Ho, M.T., et al., *Incontinence after childbearing: Long-term analysis of direct costs of conservative and surgical therapy.* The Australian and New Zealand Continence Journal, 2012. 18(1).
47. National Institute for Health and Clinical Excellence. *Urinary incontinence: the management of urinary incontinence in women (Full Guideline)*. 2006 [cited 28th Feb 2014]; Available from: http://www.nice.org.uk/nicemedia/pdf/CG40fullguideline.pdf.
48. Wagg, A., et al., *National audit of continence care for older people: results of a pilot study*.* J Eval Clin Pract, 2005. 11(6): p. 525-32.
49. Gawande, A., *The Cost Conundrum. What a Texas town can teach us about health care*, in *The New Yorker*. 2009.
50. Ginsburg, P.B., *Fee-for-service will remain a feature of major payment reforms, requiring more changes in Medicare physician payment.* Health Aff (Millwood), 2012. 31(9): p. 1977-83.
51. Krissovich, M., *The financial side of continence promotion.* Geriatr Nurs, 1998. 19(2): p. 91-4.
52. Sirls, L.T., et al., *The Effect of Urodynamic Testing on Clinical Diagnosis, Treatment Plan and Outcomes in Women Undergoing Stress Urinary Incontinence Surgery.* The Journal of urology, 2013. 189(1): p. 204-209.
53. Dubuc, N., et al., *Development of integrated care pathways: toward a care management system to meet the needs of frail and disabled community-dwelling older people.* Int J Integr Care, 2013. 13: p. e017.
54. Leichsenring, K., *Developing integrated health and social care services for older persons in Europe.* Int J Integr Care, 2004. 4: p. e10.
55. Eklund, K., et al., *One-year outcome of frailty indicators and activities of daily living following the randomised controlled trial; "Continuum of care for frail older people".* BMC Geriatr, 2013. 13(1): p. 76.
56. Kaplan, R.S. and M.E. Porter, *How to solve the cost crisis in health care.* Harv Bus Rev, 2011. 89(9): p. 46-52, 54, 56-61 passim.
57. Berg, M., et al. *Contracting value: Shifting paradigms*. 2012; Available from: http://www.kpmg.com/Global/en/IssuesAndInsights/ArticlesPublications/contracting-value/Documents/contracting-value-v7.pdf.
58. Porter, M.E., *A strategy for health care reform--toward a value-based system.* N Engl J Med, 2009. 361(2): p. 109-12.
59. McFall, S., et al., *Evaluation and treatment of urinary incontinence. Report of a physician survey.* Arch Fam Med, 1997. 6(2): p. 114-9.
60. Albers-Heitner, P., et al., *Adherence to professional guidelines for patients with urinary incontinence by general practitioners: a cross-sectional study.* J Eval Clin Pract, 2008. 14(5): p. 807-11.
61. Borrie, M.J., et al., *Interventions led by nurse continence advisers in the management of urinary incontinence: a randomized controlled trial.* CMAJ, 2002. 166(10): p. 1267-73.
62. Moore, K.H., et al., *Randomised controlled trial of nurse continence advisor therapy compared with standard urogynaecology regimen for conservative incontinence treatment: efficacy, costs and two year follow up.* BJOG, 2003. 110(7): p. 649-57.
63. Farrell, S.A., et al., *Two models for delivery of women's continence care: the step-wise continence team versus the traditional medical model.* J Obstet Gynaecol Can, 2009. 31(3): p. 247-53.
64. Oliver, R., et al., *Urogynecology triage clinic: a model of healthcare delivery.* Int Urogynecol J Pelvic Floor Dysfunct, 2009. 20(8): p. 913-7.
65. Matharu, G.S., et al., *Continence nurse treatment of women's urinary symptoms.* Br J Nurs, 2004. 13(3): p. 140-3.
66. Albers-Heitner, P., et al., *Consumer satisfaction among patients and their general practitioners about involving nurse specialists in primary care for patients with urinary incontinence.* Scand J Caring Sci, 2013. 27(2): p. 253-9.
67. Shaw, C., K.S. Williams, and R.P. Assassa, *Patients' views of a new nurse-led continence service.* J Clin Nurs, 2000. 9(4): p. 574-82.
68. Laurant, M., et al., *Substitution of doctors by nurses in primary care.* Cochrane Database Syst Rev, 2005(2): p. CD001271.

69. Albers-Heitner, P. *WHO CARES? Studying various aspects of involving nurse specialists in primary care for urinary incontinence*. 2011 [cited 2014 28th Feb]; Available from: http://arno.unimaas.nl/show.cgi?fid=23730.
70. Shaw, C., et al., *A qualitative study of the assessment and treatment of incontinence in primary care.* Fam Pract, 2007. 24(5): p. 461-7.
71. Viktrup, L. and L.A. Moller, *The handling of urinary incontinence in Danish general practices after distribution of guidelines and voiding diary reimbursement: an observational study.* BMC Fam Pract, 2004. 5: p. 13.
72. St John, W. and M. Wallis, *Outcome evaluation of a multi-disciplinary community-based continence service for Australian women.* Women Health, 2004. 40(2): p. 35-52.
73. Katon, W.J., et al., *Collaborative care for patients with depression and chronic illnesses.* N Engl J Med, 2010. 363(27): p. 2611-20.
74. Rice, K.L., et al., *Disease management program for chronic obstructive pulmonary disease: a randomized controlled trial.* Am J Respir Crit Care Med, 2010. 182(7): p. 890-6.
75. Jeffery, S., S.K. Doumouchtsis, and M. Fynes, *Patient satisfaction with nurse-led telephone follow-up in women with lower urinary tract symptoms.* J Telemed Telecare, 2007. 13(7): p. 369-73.
76. Lugtenberg, M., et al., *Current guidelines have limited applicability to patients with comorbid conditions: a systematic analysis of evidence-based guidelines.* PLoS One, 2011. 6(10): p. e25987.
77. Porter, M.E., *What is value in health care?* N Engl J Med, 2010. 363(26): p. 2477-81.
78. UK All Party Parliamentary Group For Continence Care. *Cost-effective Commissioning for Continence Care*. 2011; Available from: http://www.appgcontinence.org.uk/pdfs/CommissioningGuideWEB.pdf.
79. Haynes, R.B., P.J. Devereaux, and G.H. Guyatt, *Physicians' and patients' choices in evidence based practice.* BMJ, 2002. 324(7350): p. 1350.
80. Fox, S. and M. Duggan, *Health Online 2013*, in *Pew Internet Research*. 2013.
81. Gerber, B.S. and A.R. Eiser, *The patient physician relationship in the Internet age: future prospects and the research agenda.* J Med Internet Res, 2001. 3(2): p. E15.
82. Sung, V.W., et al., *Treatment decision-making and information-seeking preferences in women with pelvic floor disorders.* Int Urogynecol J, 2010. 21(9): p. 1071-8.
83. O'Donnell, M. and S. Hunskaar, *Preferences for involvement in treatment decision-making among Norwegian women with urinary incontinence.* Acta Obstet Gynecol Scand, 2007. 86(11): p. 1370-6.
84. Tannenbaum, C., et al., *Effectiveness of a new evidence-based self-management tool for incontinent older women*, in *International Continence Society 2010*. 2010.
85. Diokno, A.C., et al., *Prevention of urinary incontinence by behavioral modification program: a randomized, controlled trial among older women in the community.* J Urol, 2004. 171(3): p. 1165-71.
86. Milne, J.L. and K.N. Moore, *Factors impacting self-care for urinary incontinence.* Urol Nurs, 2006. 26(1): p. 41-51.
87. Stothers, L., et al., *Developing a continence care centre using an urban/academic model of continence care.* Can J Urol, 2008. 15(3): p. 4084-90.
88. Harari, D., et al. *Continence Care Services England 2013. Survey Report*. 2013 [cited 2014 28th Feb]; Available from: http://www.appgcontinence.org.uk/pdfs/Continence%20Care%20Services%20England%20Report%202013.pdf.
89. Desai, N., et al., *Provision of continence pads by the continence services in Great Britain: fair all round?* J Wound Ostomy Continence Nurs, 2008. 35(5): p. 510-4.
90. Paterson, J., et al., *Selection of continence products: perspectives of people who have incontinence and their carers.* Disabil Rehabil, 2003. 25(17): p. 955-63.
91. Deglise, C., L.S. Suggs, and P. Odermatt, *SMS for disease control in developing countries: a systematic review of mobile health applications.* J Telemed Telecare, 2012. 18(5): p. 273-81.
92. Sjostrom, M., et al., *Internet-based treatment of stress urinary incontinence: a randomised controlled study with focus on pelvic floor muscle training.* BJU Int, 2013. 112(3): p. 362-72.
93. Stephen, K., G. Rodger, and G. Cumming *Attitudes to apps for continence*. International Journal of Integrated Care, 2012. 12(Suppl1): e95.

94. de Bruin, M. *A new ehealth service for women with urinary incontinence: an online diagnostic expert program combined with a consult at home by a continence nurse*. 2013 [cited 2014 28th Feb]; Available from: http://www.ics.org/Abstracts/Publish/134/000165.pdf.
95. Wicks, P., et al., *Innovations in e-health.* Qual Life Res, 2013.
96. Munneke, M., et al., *Efficacy of community-based physiotherapy networks for patients with Parkinson's disease: a cluster-randomised trial.* Lancet Neurol, 2010. 9(1): p. 46-54.
97. Hsiao, C.J., J.A. Marsteller, and A.E. Simon, *Electronic Medical Record Features and Seven Quality of Care Measures in Physician Offices.* Am J Med Qual, 2013.
98. National Institute for Health and Clinical Excellence. *Urinary incontinence. The management of urinary incontinence in women*. NICE clinical guideline 171 2013 [cited 2014 28th Feb]; Available from: http://www.nice.org.uk/nicemedia/live/14271/65143/65143.pdf.
99. Kelleher, C., et al., *Patient-Reported Outcome Assessment*, in *Incontinence*, P. Abrams, et al., Editors. 2013, ICUD: Belgium. p. 389-428.
100. Royal College of Physicians, *National audit of continence care (NACC). Pilot audit evaluation report*. 2012.
101. Ploeg, J., et al., *The role of nursing best practice champions in diffusing practice guidelines: a mixed methods study.* Worldviews Evid Based Nurs, 2010. 7(4): p. 238-51.
102. Corrigan, P. and S. Laitner. *The Accountable Lead Provider. Developing a powerful disruptive innovator to create integrated and accountable programmes of care*. RightCare Casebook 2012 [cited 2014 28th Feb]; Available from: http://www.rightcare.nhs.uk/downloads/Rightcare_Casebook_accountable_lead_provider_Aug2012.pdf.
103. Cardozo, L. and D. Staskin, *Textbook of Female Urology and Urogynecology. 2nd Edition*. 2006. p. 91-98.
104. Royal College of Nursing. *Improving continence care for patients. The role of nurses*. 2006 [cited 2014 28th Feb]; Available from: http://www.rcn.org.uk/__data/assets/pdf_file/0003/78555/001952.pdf.

Appendix 1 Members of the expert panel

**Professor Adrian Wagg**	Professor of Healthy Aging, Department of Medicine, University of Alberta, Alberta, Canada
**Diane Newman**	Nurse Practitioner, Adjunct Associate Professor of Urology in Surgery, Co-Director, Penn Center of Continence and Pelvic Health, Division of Urology, Univesity of Pennsylvania, Pennsylvania, United States
**Dr Paul van Houten**	Care of the Elderly physician. expert in continence care, Zonnhuisgroep Amstelland, Elderly Medicine, Amstelveen, the Netherlands
**Kai Leichsenring**	Researcher, European Centre for Social Welfare Policy and Research, Vienna, Austria

Appendix 2 Clinical guidelines by country

Citation	Year	Topic	Professional Organisation	Country	Language
**Lovatsis et al**	2010	Evaluation and treatment of recurrent UI after pelvic floor surgery	Canadian Task Force on Preventive Health Care	Canada	English<br>French
**Aubert et al**	2010	Primary nocturnal enuresis	French Expert Consensus Panel	France	French
**Hermieu et al**	2010	Synthesis of the guidelines for the treatment of non-neurological urinary incontinence in women	L'Association Francaise d'Urologie	France	French
	2010	Recommendations for the treatment of non-neurological urinary incontinence in women	L'Association Francaise d'Urologie	France	French
**Fritel et al**	2009	Diagnosis and management of adult female stress urinary incontinence	French College of Gynaecologists and Obstetricians	France	
**Lucas et al**<br>**Thuroff et al**<br>**Schroder et al**	2013<br>2011<br>2010	EAU Guidelines on Urinary Incontinence	European Association of Urology	International	Spanish
**Drutz et al**	2010	Training for female pelvic medicine and reconstructive pelvic surgery (education)	International Urogynecological Association (IUGA)	International	English
**Ghoniem, 2008**	2008	Evaluation and management of stress urinary incontinence in women	International Urogynecological Assoication (IUGA)	International	English
**Yamaguchi et al**	2009	Clinical guidelines for overactive bladder	Japanese Urological Association Neurogenic Bladder Society	Japan	English
**Nishizawa**	2008	Urinary incontinence in the elderly		Japan	Japanese (English translation)
**Velazquez Sanchez Mdel et al**	2009	Diagnosis and management of urinary incontinence	Consensus panel	Mexico	Spanish
**Garcia-Gonzalez**	2008	Diagnosis and treatment of urinary incontinence in the elderly	Consensus panel	Mexico	Spanish
**Radziszewski et al**	2010	Urinary incontinence and overactive bladder in women	Consensus panel	Poland	Polish

Citation	Year	Topic	Professional Organisation	Country	Language
**Polish Society of Obstetrics & Gynecology**	2009	Prevention urinary incontinence and pelvic organ prolapse in women undergoing hysterectomy	Polish Society of Obstetrics & Gynecology	Poland	Polish
**van Pinxteren**	2008	Urinary incontinence in all patients	Dutch College of General Practitioners	The Netherlands	Dutch
**Scottish Intercollegiate Guidelines Network**	2004	Management of urinary incontinence in primary care	Scottish Intercollegiate Guidelines Network	Scotland	English
**NICE**	2013	Urinary incontinence: the management of urinary incontinence in women	National Institute for Health and Clinical Excellence (NICE)	United Kingdom	English
**NICE**	2010	Lower Urinary Tract Symptoms (LUTS)	National Institute for Health and Clinical Excellence (NICE)	United Kingdom	English
**NICE**	2010	Nocturnal Enuresis, the management of bedwetting in children and young people	National Institute for Health and Clinical Excellence (NICE)	United Kingdom	English
**Fowler et al**	2009	Bladder management in multiple sclerosis	Consensus panel	United Kingdom	English
**NICE**	2007	Faecal incontinence: the management of faecal incontinence in adults	National Institute for Health and Clinical Excellence (NICE)	United Kingdom	English
**Gormley et al**	2012	Diagnosis and Treatment of Overactive Bladder (non-neurogenic) in adults	American Urological Association / Society for Urodynamics and Female Urology	USA	English
**Winters et al**	2012	Adult Urodynamics	American Urological Association / Society for Urodynamics and Female Urology	USA	English
**Hanno et al**	2011	Interstitial cystitis / bladder pain syndrome	American Urological Association	USA	English
**Dimochowski et al**	2010	Surgical management of stress urinary incontinence in women	American Urological Association	USA	English
**ACOG**	2005	Urinary incontinence in women	American College of Obstetrics and Gynecology	USA	English

The information contained herein is of a general nature and is not intended to address the circumstances of any particular individual or entity. Although we endeavour to provide accurate and timely information, there can be no guarantee that such information is accurate as of the date it is received or that it will continue to be accurate in the future. No one should act on such information without appropriate professional advice after a thorough examination of the particular situation.

March 2014
